# Inhibition of N-myristoyltransferase in pluripotent stem cells promotes the naive state in mice and elicits trophectoderm and primitive endoderm markers in humans

**DOI:** 10.1016/j.stemcr.2025.102610

**Published:** 2025-08-28

**Authors:** Junko Yoshida, Hitomi Watanabe, Kaori Yamauchi, Takumi Nishikubo, Ayako Isotani, Satoshi Ohtsuka, Hitoshi Niwa, Yuki Kawamoto, Hidenori Akutsu, Akihiro Umezawa, Hirofumi Suemori, Yasuhiro Takashima, Hideo Matsuda, Gen Kondoh, Junji Takeda, Kyoji Horie

**Affiliations:** 1 Department of Physiology II, Nara Medical University, 840 Shijo-cho, Kashihara, Nara 634-8521, Japan; 2 Department of Genome Biology, Graduate School of Medicine, Osaka University, 2-2 Yamadaoka, Suita, Osaka 565-0871, Japan; 3 Laboratory of Integrative Biological Science, Institute for Life and Medical Sciences, Kyoto University, 53 Shogoin Kawahara-cho, Sakyo-ku, Kyoto 606-8507, Japan; 4 Laboratory of Embryonic Stem Cell Research, Institute for Life and Medical Sciences, Kyoto University, 53 Shogoin Kawahara-cho, Sakyo-ku, Kyoto 606-8507, Japan; 5 Genome Information Research Center, Research Institute for Microbial Diseases, Osaka University, 3-1 Yamadaoka, Suita, Osaka 565-0871, Japan; 6 Division of Biological Science, Graduate School of Science and Technology, Nara Institute of Science and Technology, 8916-5 Takayama-cho, Ikoma, Nara 630-0192, Japan; 7 Pluripotent Stem Cell Studies, RIKEN Center for Developmental Biology, 2-2-3 Minatojima-minamimachi, Chuo-ku, Kobe, Hyogo 650-0047, Japan; 8 Laboratory for Experimental Animals, Kyoto Prefectural University of Medicine, 465 Kajii-cho, Kamigyo-ku, Kyoto 602-8566, Japan; 9 Department of Pluripotent Stem Cell Biology, Institute of Molecular Embryology and Genetics, Kumamoto University, 2-2-1 Honjo, Chuo-ku, Kumamoto 860-0811, Japan; 10 Center for Regenerative Medicine, National Center for Child Health and Development, 2-10-1 Okura, Setagaya-ku, Tokyo 157-8535, Japan; 11 Department of Life Science Frontiers, Center for iPS Cell Research and Application (CiRA), Kyoto University, 53 Shogoin Kawahara-cho, Sakyo-ku, Kyoto 606-8507, Japan; 12 Department of Bioinformatic Engineering, Graduate School of Information Science and Technology, Osaka University, 1-5 Yamadaoka, Suita, Osaka 565-0871, Japan; 13 Laboratory of Immunoglycobiology, Research Institute for Microbial Diseases, Osaka University, 3-1 Yamadaoka, Suita, Osaka 565-0871, Japan

**Keywords:** embryonic stem cells, ESCs, induced pluripotent stem cells, iPSCs, epiblast stem cells, EpiSCs, N-myristoyltransferase, NMT, naive state, primed state, SRC

## Abstract

Naive and primed states represent distinct phases of pluripotency during early embryonic development, both of which can be captured and interconverted *in vitro*. To understand pluripotency regulation, we performed a recessive genetic screen using homozygous mutant mouse embryonic stem cells (mESCs) and identified *N*-myristoyltransferase (NMT) as a novel regulator. Disruption of *Nmt1* in mESCs conferred resistance to differentiation, and NMT suppression in mouse epiblast stem cells (mEpiSCs) promoted the conversion from the primed to the naive state. This effect was independent of proto-oncogene tyrosine-protein kinase Src (SRC), which is a major substrate of NMT and is known to promote mESC differentiation. In contrast, NMT suppression in naive-state human induced pluripotent stem cells (hiPSCs) partially induced naive markers but, more notably, expanded subpopulations expressing trophectoderm and primitive endoderm markers, most of which co-expressed the pluripotency marker *POU5F1*. These results identify NMT as a novel regulator of pluripotency, with distinct roles in mice and humans.

## Highlights

• Nmt1 loss confers differentiation resistance and upregulates naive markers in mESCs
• Nmt suppression in mEpiSCs promotes primed-to-naive state conversion
• Nmt regulates pluripotency independently of its major substrate SRC
• Nmt suppression in naive hiPSCs expands cells co-expressing *POU5F1* and TE/PE markers

## Graphical abstract

**Figure:**
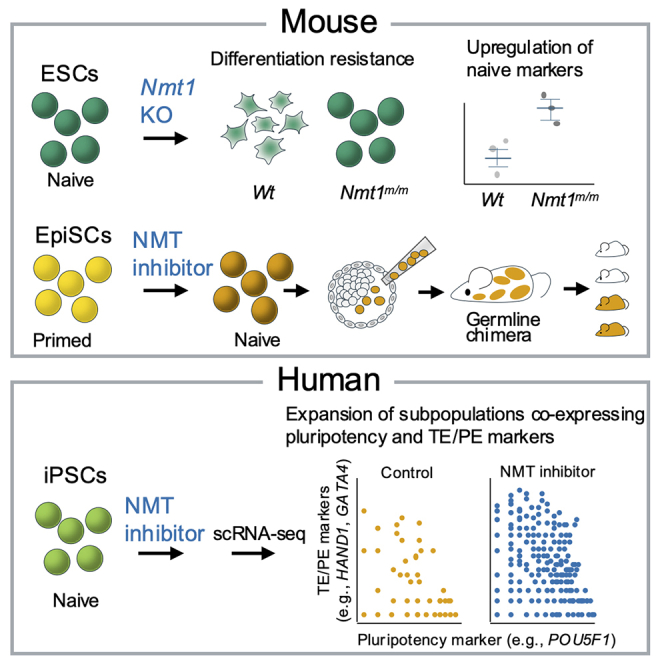

## Teaser

Horie and colleagues identify *N*-myristoyltransferase (NMT) as a key regulator of pluripotency. NMT suppression confers differentiation resistance and promotes naive-state acquisition in mouse stem cells independently of *SRC*. In human iPSCs, it expands subpopulations expressing *trophectoderm* and *primitive endoderm* markers, most of which co-express *POU5F1*, thereby revealing species-specific roles for NMT in controlling pluripotent stem cell identity.

## Introduction

Pluripotency is the ability to differentiate into three primary germ layers and, subsequently, adult tissues. Over recent decades, various approaches have been used to capture the pluripotent state in cell cultures (Rossant and Tam, 2017), revealing distinct pluripotent states in both mice and humans. The most widely studied are the naive and primed states. The naive state corresponds to pre-implantation embryos, from which mouse embryonic stem cells (mESCs) are derived (Evans and Kaufman, 1981), while the primed state reflects post-implantation embryos, from which mouse epiblast stem cells (mEpiSCs) are established (Brons et al., 2007; Tesar et al., 2007). In contrast, human embryonic stem cells (hESCs) were in the primed state under conventional culture conditions, despite being derived from pre-implantation embryos (Thomson et al., 1998). Human induced pluripotent stem cells (hiPSCs) were also in the primed state when established under the conventional hESC culture media (Takahashi et al., 2007; Yu et al., 2007). Naive-state hESCs/hiPSCs have since been established using chemical pathway modulation or transient expression of transcription factors (Takashima et al., 2014; Theunissen et al., 2014). More recently, an intermediate “formative” pluripotent state has also been described in both species (Kalkan et al., 2017; Kinoshita et al., 2021; Smith, 2017). Elucidation of the regulatory mechanisms of these distinct states may advance our understanding of pluripotency and supports regenerative medicine applications.

We previously developed a method to generate homozygous mutant mESCs by converting heterozygosity to homozygosity via transient *Blm* gene inactivation (Horie et al., 2011). Expanding this approach, we established nearly 200 homozygous mutant lines, especially for genes with unknown functions. Through phenotypic screening, we found that *Nmt1*-homozygous mutant mESCs resists differentiation. *N*-myristoyltransferase (NMT) is an enzyme that attaches a myristoyl group to protein N termini (Yuan et al., 2020). Inhibition of NMT activity induced naive-state markers in mESCs and promoted the conversion of primed-state mEpiSCs to the naive state. In contrast, in hiPSCs, NMT inhibition expanded subpopulations expressing trophectoderm and primitive endoderm markers, most of which co-expressed the pluripotency marker *POU5F1*. These findings suggest that NMT is a novel regulator of pluripotency with distinct functions in mouse and human cells.

## Results

### Disruption of *Nmt1* confers differentiation resistance to mESCs and enhances the properties of the naive state

**Figure 1:**
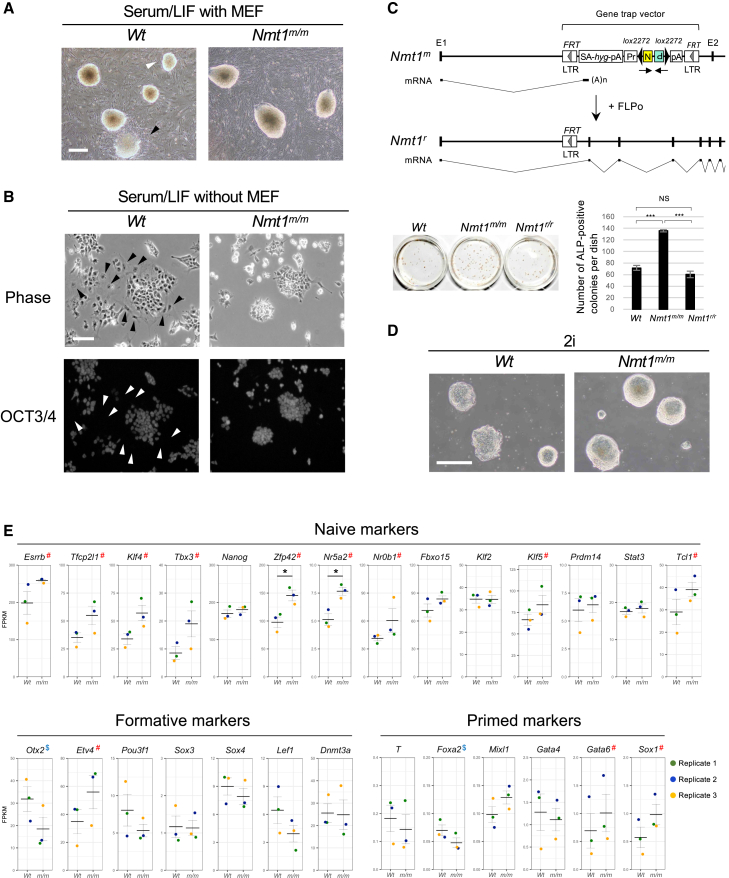
Disruption of *Nmt1* confers differentiation resistance to mESCs and enhances properties of the naive state (A) Morphological differences between wild-type (WT) and *Nmt1*-homozygous mutant (*Nmt1*^*m/m*^) mESC colonies in serum/LIF medium. mESCs were sparsely plated on MEFs to obtain single cell-derived colonies. Flat (black arrowhead) or small-sized (white arrowhead) colonies were observed in *WT* mESCs, whereas *Nmt1*^*m/m*^ mESCs were more homogeneous in shape and size and noticeably dome shaped. Scale bar: 500 μm. (B) OCT3/4 staining of mESCs cultured for 12 days in serum/LIF medium without MEFs. *Nmt1*^*m/m*^ mESCs formed compact colonies with homogeneous OCT3/4 staining, whereas WT mESCs exhibited irregular-shaped colonies with scattered cells. Note that some WT mESCs are enlarged and negative for OCT3/4 (arrowheads), indicating differentiation. Scale bar: 50 μm. (C) Reversion of differentiation resistance by deletion of the gene trap vector. (Top) Generation of the revertant allele (*Nmt1*^*r*^) from the mutant allele (*Nmt1*^*m*^) by Flp/*FRT* recombination. (Bottom) Differentiation resistance in each genotype. Six hundred mESCs were plated on MEFs in serum-containing medium without LIF, and the number of undifferentiated colonies was determined by ALP staining. Data are shown as mean ± SEM (*n* = 3, independent replicates). Tukey-Kramer test; ^∗∗∗^*p* < 0.001; NS, not significant (*p* > 0.05). E, exon; LTR, long terminal repeat; SA, splice acceptor; *hyg*, hygromycin resistance gene; pA, polyadenylation signal; Pr, *Pgk1* promoter; N, neomycin-resistance gene; P, fusion gene of the puromycin-resistance gene and the herpes simplex virus thymidine kinase gene. Arrows below the gene trap vector indicate the orientation of each selection marker. (D) Morphological differences in single cell-derived colonies between WT and *Nmt1*^*m/m*^ mESCs in serum-free 2i medium without LIF and MEFs. Note that *Nmt1*^*m/m*^ mESC colonies are noticeably dome shaped compared to WT mESC colonies. Scale bar: 500 μm. (E) RNA-seq analysis presented as mean ± SEM from three independent experiments in serum/LIF medium. Unpaired Welch’s t test; ^∗^*p* < 0.05. “#” and “$” indicate genes consistently up- or downregulated, respectively, across all three experiments. FPKM, fragments per kilobase per million mapped reads.

As part of our phenotypic screening (Horie et al., 2011), we cultured single cell-derived colonies of each mESC clone on mouse embryonic fibroblasts (MEFs) in serum-containing medium. While wild-type (WT) mESCs occasionally formed flat or small-sized colonies (Figure 1A, left), *Nmt1*-homozygous mutant mESCs formed uniform, dome-shaped colonies (Figure 1A, right), suggesting resistance to differentiation. To test this, we cultured cells in serum-containing medium without MEFs. WT mESCs displayed differentiation-associated features (e.g., enlarged cells and reduced OCT3/4), whereas *Nmt1* mutants formed compact, OCT3/4-positive colonies (Figure 1B), confirming differentiation resistance. This phenotype was reversed by excising the gene trap vector via Flp/*FRT* recombination, resulting in fewer alkaline phosphatase (ALP)-positive colonies (Figure 1C). In serum-free 2i (inhibitors of MAPK/ERK kinase, MEK and glycogen synthase kinase 3, GSK3) conditions without leukemia inhibitory factor (LIF), which is known to stabilize naive state and is called ground state condition (Ying et al., 2008), both WT and *Nmt1*-mutant mESCs formed undifferentiated colonies, but the latter displayed enhanced dome-shaped morphology (Figure 1D), suggesting that NMT1 deficiency promotes the naive state.

To confirm that NMT1 deficiency promotes the naive state in mESCs, we performed RNA sequencing (RNA-seq) on three independent cultures (Figure 1E). We analyzed the expression of naive, formative, and primed markers (Carbognin et al., 2023; Kalkan et al., 2017; Kinoshita et al., 2021; Smith, 2017). *Nmt1* mutants consistently showed elevated expression of naive-state markers; mean fragments per kilobase per million mapped reads (FPKM) values increased for all except *Klf2*, and 9 markers increased in all experiments, though only two reached statistical significance. In contrast, formative and primed markers varied. These findings suggest that NMT1 deficiency specifically promotes the naive state in mESCs.

### Conversion of primed-state mEpiSCs into mESC-like naive-state cells by an NMT inhibitor

**Figure 2:**
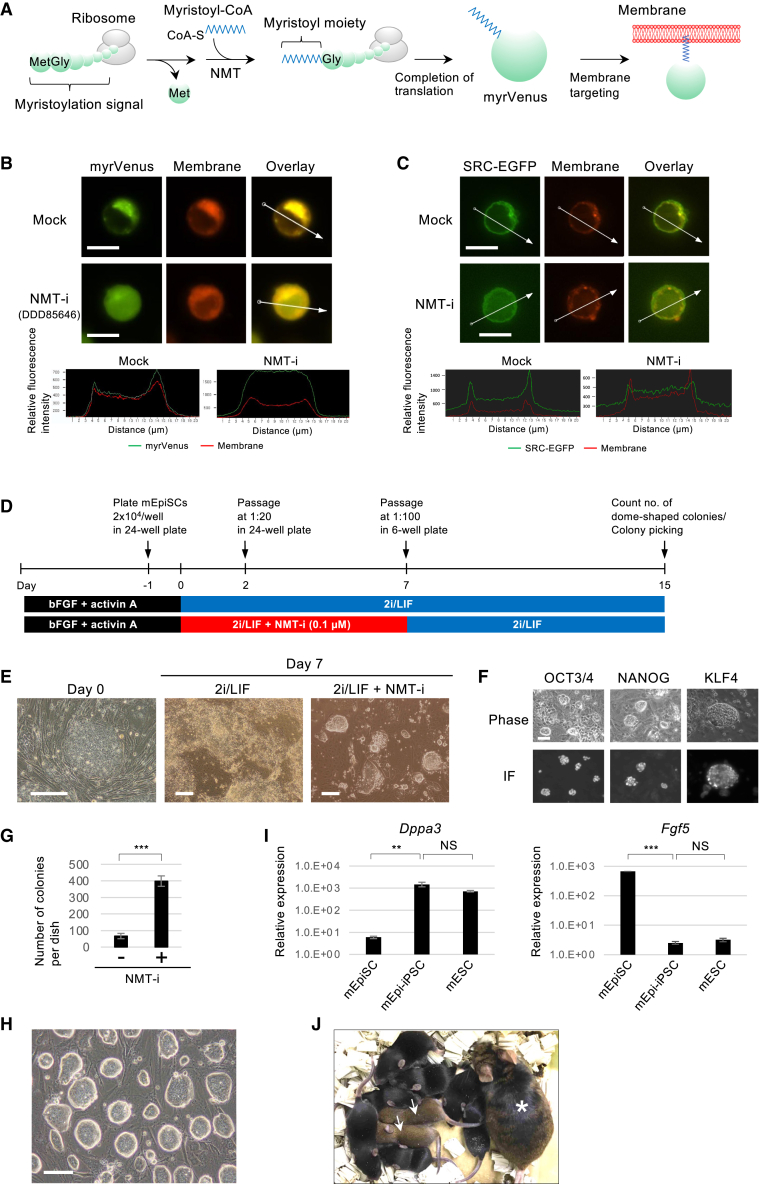
Conversion of primed-state mEpiSCs into mESC-like naive-state cells by the NMT inhibitor (A) Schematic of translation, myristoylation, and membrane targeting of the *N*-myristoylation signal-containing Venus reporter (myrVenus). (B and C) The effect of the NMT inhibitor DDD85646 on subcellular localization of myrVenus (B) and SRC-EGFP (C) in mESCs. Line plots indicate the relative fluorescence intensity of fluorescent proteins and membrane staining along the white arrow shown in the overlaid picture. The plasma membrane localization of fluorescent proteins is decreased in the presence of the NMT inhibitor. Scale bar: 10 μm. NMT-i, NMT inhibitor. (D) Schematic of the protocol for the conversion of primed-state mEpiSCs into the mESC-like naive state. (E) Colony morphology at day 0 and day 7. Dome-shaped mESC-like colonies appeared with the NMT inhibitor. Scale bar: 200 μm. (F) Immunostaining of cells cultured under 2i/LIF + NMT inhibitor. Dome-shaped colonies were positive for the pluripotency markers OCT3/4 and NANOG and the naive state-specific marker KLF4. IF, immunofluorescence. Scale bar: 50 μm. (G) The number of dome-shaped colonies at day 15. Data are shown as mean ± SEM (*n* = 3, independent replicates). Unpaired Student’s t test; ^∗∗∗^*p* < 0.001. (H) mESC-like cells stably maintained under 2i/LIF without the NMT inhibitor. Scale bar: 500 μm. (I) mRNA expression of the naive-state marker *Dppa3* and the primed-state marker *Fgf5*. mEpi-iPSC indicates mESC-like cell induced from mEpiSC. Data are shown as mean ± SEM (*n* = 3, independent replicates). Dunnett’s test; ^∗∗^*p* < 0.01 and ^∗∗∗^*p* < 0.001; NS, not significant (*p* > 0.05). (J) Germline transmission of mEpi-iPSCs. An asterisk indicates a female parent chimera, and arrows denote agouti color-coated offspring.

Given the observed phenotypes, we hypothesized that NMT1 inhibition may facilitate the conversion of primed-state mEpiSCs into mESC-like naive-state cells. To test this possibility, we used an NMT inhibitor DDD85646 (Frearson et al., 2010), originally reported as a lead compound against NMT of *Trypanosoma brucei*. Expression of an *N*-myristoylation signal-containing Venus (myrVenus) reporter (Rhee et al., 2006) (Figure 2A) showed membrane localization that was disrupted by the inhibitor (Figure 2B), indicating effective NMT inhibition. Proto-oncogene tyrosine-protein kinase Src (SRC) undergoes *N*-myristoylation and localizes to cell membranes (Patwardhan and Resh, 2023). To validate DDD85646 functionality, we examined its effect on SRC-EGFP localization. DDD85646 reduced membrane localization of SRC-EGFP (Figure 2C), confirming its inhibition on NMT activity in mammalian cells.

Next, we tested whether the NMT inhibitor facilitates the conversion of the primed-state mEpiSCs derived from post-implantation embryos into the mESC-like naive-state cells (Figure 2D). High levels of cell death and differentiation were observed in mEpiSCs under 2i/LIF, an optimal culture condition for naive-state mESCs (Figure 2E, middle), consistent with prior reports (Brons et al., 2007; Tesar et al., 2007). A combination of 2i/LIF and the NMT inhibitor DDD85646 also induced cell death and differentiation; however, dome-shaped mESC-like colonies appeared after 1 week (Figure 2E, right) and were positive for the pluripotency markers OCT3/4 and NANOG and the naive state-specific marker KLF4 (Figure 2F). Upon replating and continued culture in 2i/LIF, the increase in colony number became evident and quantifiable in the presence of the NMT inhibitor (Figure 2G). Subsequently, mESC-like clones could be established from each colony under 2i/LIF without the NMT inhibitor (Figure 2H). The gene expression pattern of these clones, named mEpi-iPSC clones, resembled naive-state mESCs, with high expression of the naive-state marker *Dppa3* and low expression of the primed-state marker *Fgf5* (Figure 2I). To further demonstrate the conversion to the naive state, we injected each mEpi-iPSC clone into pre-implantation mouse embryos and generated chimeric mice. The efficiency of generating chimeric mice is extremely low when using mEpiSCs (Brons et al., 2007; Tesar et al., 2007); however, six out of seven mEpi-iPSC clones contributed to chimeric mice, and four transmitted to germline (Figures 2J and S1A), confirming that these clones were in the naive state. It should be noted that this germline transmission cannot be considered a direct measurement of the effect of the NMT inhibitor, as the process of selecting individual colonies for clone establishment may inherently favor high-quality clones. Nevertheless, the gene expression profiles observed in the bulk cell culture (Figures 2F and 2I) support a conversion effect by NMT inhibition.

### Validation of the effect of NMT1 deficiency on the primed-to-naive conversion by conditional *Nmt1* knockout

**Figure 3:**
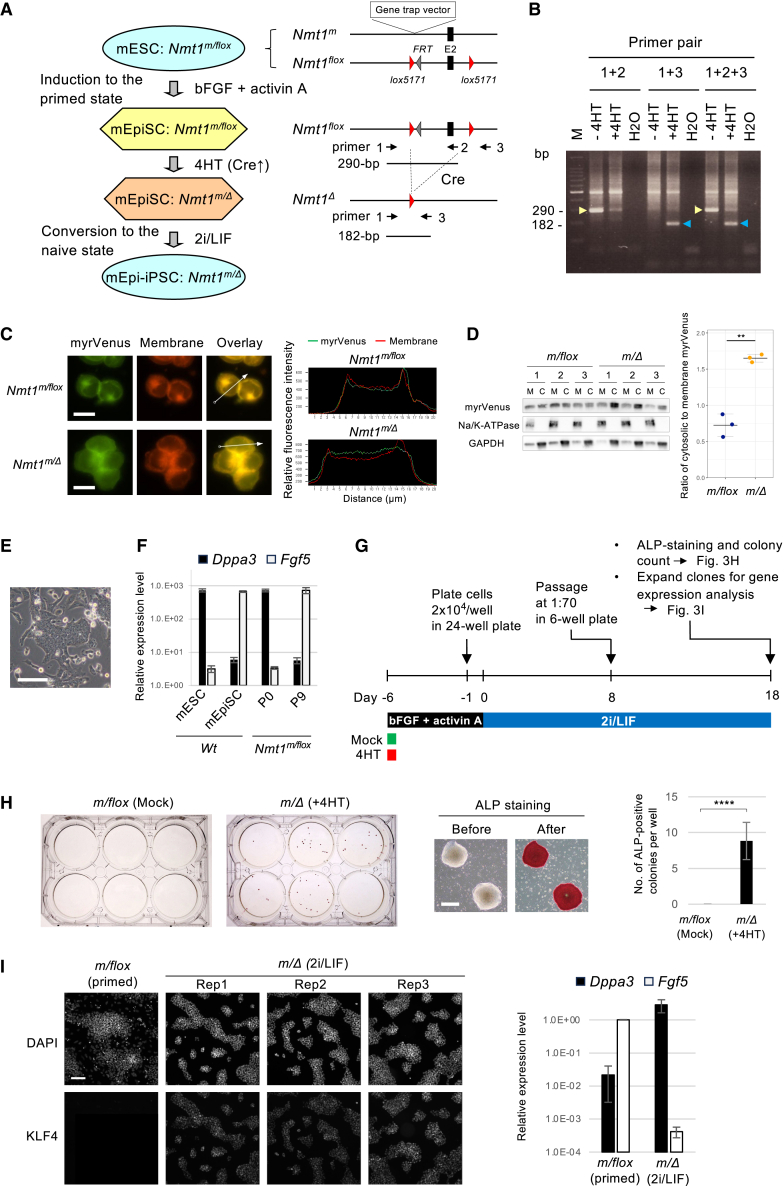
Verification of the effect of NMT1 deficiency by conditional gene knockout (A) Outline of the generation of genetically modified mEpiSC-like cells and conversion to naive-state mEpi-iPSCs by conditional knockout of *Nmt1*. 4HT, 4-hydroxytamoxifen. (B) PCR analysis of the 4HT-induced deletion of the *Nmt1* gene. Primer pairs are depicted in (A). PCR bands derived from the *Nmt1*-floxed allele and the 4HT-induced deleted allele are indicated by yellow and blue arrowheads, respectively. M, 100-bp size marker. (C) Effect of *Nmt1* knockout on subcellular localization of the myrVenus reporter. Line plots indicate the relative fluorescence intensity of myrVenus and membrane staining along the white arrows. Plasma membrane localization is decreased upon knockout. Scale bar: 10 μm. (D) Western blot analysis of the effect of the *Nmt1* knockout on subcellular localization of myrVenus. M, membrane fraction; C, cytosol fraction. Na/K-ATPase and GAPDH were used as reference for the enrichment of the membrane and cytosol fractions, respectively. Data are shown as mean ± SEM (*n* = 3, independent replicates). Unpaired Welch’s t test; ^∗∗^*p* < 0.01. (E) Morphology of mEpiSC-like cells induced from mESCs at passage 9 under bFGF and activin A. Scale bar: 100 μm. (F) Validation of primed-state identity in cells from (E) by RT-qPCR. P0, passage 0; P9, passage 9. Data are shown as mean ± SEM (*n* = 3, independent replicates). (G) Schematic of the protocol for the conversion of primed-state mEpiSC-like cells into the naive-state mEpi-iPSCs. (H) (Left) ALP staining of colonies post-conversion. (Middle) Representative of ALP-positive colonies. Scale bar: 500 μm. (Right) Number of ALP-positive colonies are shown as mean ± SEM (*n* = 3, independent replicates). Unpaired Student’s t test; ^∗∗∗∗^*p* < 0.001. (I) Naive-state confirmation by immunostaining (left) and RT-qPCR (right). '*m/flox* (primed)' indicates *Nmt1*^*m/flox*^ mEpiSC-like cells prior to conversion, while '*m/Δ* (2i/LIF)' refers to Cre-induced NMT1-deficient cells converted to the naive state. Scale bar: 100 μm. RT-qPCR data are shown as mean ± SEM (*n* = 3, independent replicates).

To confirm that the effect of the NMT inhibitor DDD85646 on the primed-to-naive conversion was not the off-target effect but the on-target effect, we genetically inactivated the *Nmt1* gene in the primed state and examined whether this induces a naive state, as outlined in Figure 3A. First, we converted the WT allele of the *Nmt1*-mutant heterozygous mESC line into the floxed allele (Figures 3A and S1B). The parental mESC line of this mutant harbors *ERT2-iCre-ERT2* at the *Rosa26* locus (Casanova et al., 2002; Horie et al., 2011), allowing conditional inactivation of *Nmt1* by 4-hydroxytamoxifen (4HT). Next, we differentiated the floxed mESCs into mEpiSC-like primed-state cells using basic fibroblast growth factor (bFGF) and activin A according to the published protocol (Guo et al., 2009) (Figure 3A). Last, we inactivated *Nmt1* in the primed state using 4HT and cultured under 2i/LIF to examine whether NMT1 deficiency triggers naive conversion.

Deletion of *Nmt1* was confirmed by PCR (Figure 3B) and led to reduced membrane localization of the myrVenus reporter (Figure 3C). This was further validated by fractionation and western blot analysis of membrane- and cytosol-localized myrVenus (Figure 3D). However, residual membrane localization of myrVenus remained (Figures 3C and 3D), suggesting additional mechanisms. There are two *Nmt* genes in mice, *Nmt1* and *Nmt2* (Yang et al., 2005). We speculate that the residual localization is due to NMT2 activity for the following reasons. First, our RNA-seq analysis detected NMT2 expression in both WT and *Nmt1*-homozygous mutant mESCs (Figure S1C). Second, we previously isolated an *Nmt2* mutant mESC clone by gene trap (Horie et al., 2011), registered in GenBank (GenBank: AB735704.1.) and in Exchangeable Gene Trap Clones (EGTC: https://egtc.jp/clones/K19F03), a member of the International Gene Trap Consortium. The gene trap vector includes a splice acceptor-hygromycin resistance cassette to identify insertions into expressed genes (Horie et al., 2011). This indicates that NMT2 is expressed at a level sufficient to confer hygromycin resistance in mESCs.

Continuous culture of mESCs under bFGF and activin A yielded mEpiSC-like flat colonies (Figure 3E). mEpiSC-like features were confirmed by a decrease in the naive-state marker *Dppa3* and the induction of the primed-state marker *Fgf5* (Figure 3F). We then inactivated the *Nmt1* gene with 4HT and cultured the cells under 2i/LIF to induce conversion to the naive state (Figure 3G). ALP-positive dome-shaped colonies appeared upon *Nmt1* inactivation but not with mock treatment (Figure 3H). These colonies were confirmed to be in the naive state by KLF4 immunostaining (Figure 3I, Left) and quantitative reverse-transcription PCR (RT-qPCR) of *Dppa3* and *Fgf5* (Figure 3I, Right). The results were consistent with the observation in the NMT inhibitor (Figure 2), demonstrating that NMT1 deficiency promotes conversion from the primed to the naive state.

### The effect of the NMT inhibitor on the primed-to-naive conversion is not mediated by SRC signaling pathways

Next, we searched for NMT substrates involved in naive pluripotency regulation. Among many known targets (Yuan et al., 2020), we focused on SRC for the following reasons. First, an SRC inhibitor supports the maintenance of the naive state in mESCs and can replace the MEK inhibitor in serum-free 2i/LIF culture (Shimizu et al., 2012). Second, an SRC inhibitor was included in a chemical cocktail for establishing naive human pluripotent stem cells (hPSCs), specifically hESCs and hiPSCs (Theunissen et al., 2014). These findings led us to consider that the effects of the NMT inhibitor observed in our study may be mediated via SRC inhibition.

**Figure 4:**
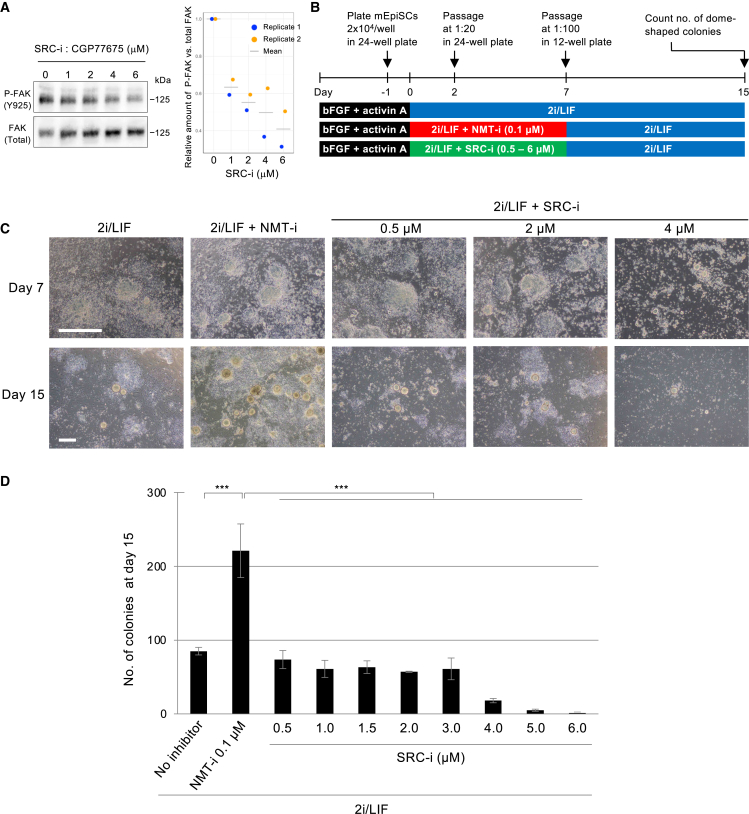
The effect of the NMT inhibitor on the conversion of the primed-to-naive state is not mediated by SRC signaling pathways (A) Western blot analysis demonstrating the dose-dependent inhibitory effect of the SRC inhibitor CGP77675 on FAK phosphorylation at Y925 in mEpiSCs. The immunoblot for replicate 1 is shown on the left, and that for replicate 2 is presented in Figure S1D. SRC-i, SRC inhibitor. (B) Schematic of the protocol for the comparison of the primed-to-naive state conversion efficiency between NMT and SRC inhibitors. (C) A morphological view of the cells during conversion. Scale bar: 500 μm. (D) The number of dome-shaped colonies at day 15. Data are shown as mean ± SEM (*n* = 3, independent replicates). Tukey-Kramer test; ^∗∗∗^*p* < 0.001.

To test this possibility, we treated mEpiSCs with the SRC inhibitor CGP77675 (Missbach et al., 1999; Shimizu et al., 2012) and compared the effect on primed-to-naive conversion with that of the NMT inhibitor DDD85646 (Figure 4). We confirmed the dose-dependent inhibitory effect of CGP77675 on SRC kinase activity by measuring focal adhesion kinase (FAK) phosphorylation at tyrosine 925 (FAK-Y925), a major SRC target (Schlaepfer and Hunter, 1996) (Figures 4A and S1D). Based on the previous report identifying 1.5 μM as an optimal concentration for maintaining the naive state in mESCs (Shimizu et al., 2012), we tested a range of 0.5–6 μM (Figures 4B and 4C). However, no increase in conversion was observed (Figure 4D), and higher doses (≥4 μM) severely suppressed cell growth (Figure 4C). These results indicate that factors other than SRC mediate the effect of the NMT inhibitor on primed-to-naive conversion.

### NMT suppression induces the expression of markers for trophectoderm and primitive endoderm in naive hiPSCs, as identified by bulk RNA-seq

To assess whether the effect of NMT suppression is conserved in humans, we first attempted primed-to-naive conversion of hESCs/hiPSCs using 2i medium with human LIF and the NMT inhibitor. However, cells differentiated gradually and undifferentiated cells were lost (Figure S2), indicating that this condition is insufficient for primed-to-naive conversion.

**Figure 5:**
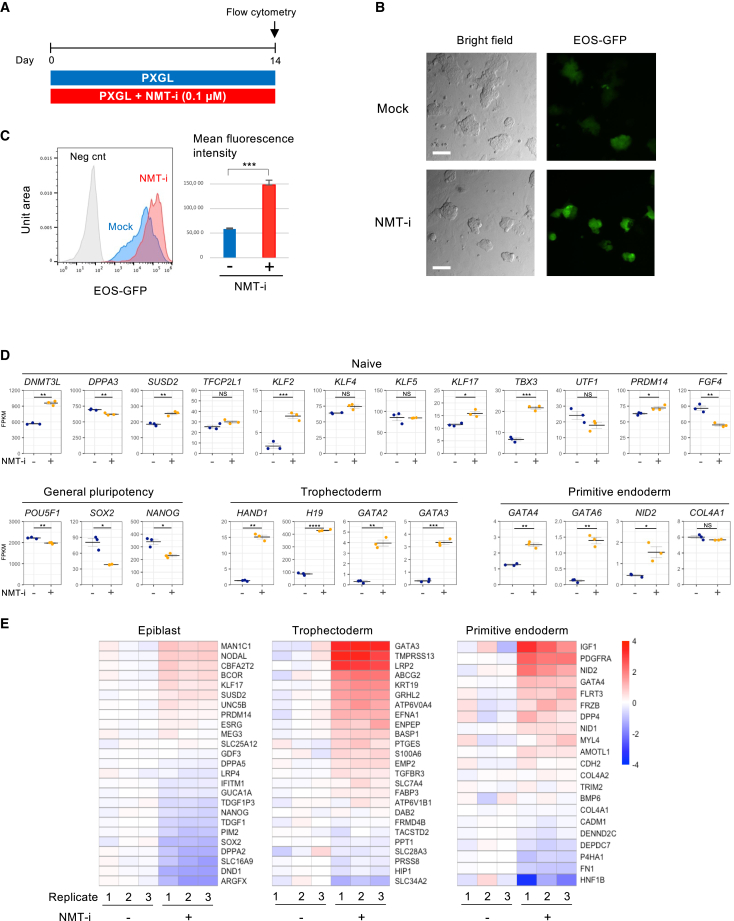
NMT suppression induces the expression of markers for trophectoderm and primitive endoderm in naive hiPSCs, as identified by bulk RNA-seq (A) Schematic of the protocol for assessing the effect of the NMT inhibitor on naive hiPSCs in PXGL medium. (B) Morphology of naive hiPSCs and EOS-GFP reporter expression. Scale bar: 100 μm. (C) Flow cytometric analysis of EOS-GFP expression. Negative control is primed-state hiPSCs. The mean fluorescence intensity of EOS-GFP is shown as mean ± SEM (*n* = 3, independent replicates). Unpaired Student’s t test; ^∗∗∗^*p* < 0.001. (D) Bulk RNA-seq analysis presented as mean ± SEM from three independent cultures. Unpaired Welch’s t test; ^∗^*p* < 0.05, ^∗∗^*p* < 0.01, and ^∗∗∗^*p* < 0.001; NS, not significant (*p* > 0.05). (E) Relative expression levels of the epiblast-, trophectoderm-, and primitive endoderm-specific genes (Petropoulos et al., 2016).

Next, we investigated whether the NMT inhibitor could enhance naive features in already naive hiPSCs using the EOS-GFP reporter (Hotta et al., 2009), originally developed to identify primed-state hiPSCs and later found to be highly induced in the naive state (Takashima et al., 2014). Adipocyte-derived primed-state hiPSCs were converted to a naive state on MEF feeder cells in t2iLGö medium (Guo et al., 2017), and naive hiPSCs were separated from MEFs by the expression of SUSD2, a naive state-specific cell surface marker (Bredenkamp et al., 2019a) (Figures S3A and S3B). Within the SUSD2-positive hiPSCs, we observed EOS-GFP-negative cells (Figure S3C), suggesting incomplete conversion. Growth retardation also occurred with 0.1 μM inhibitor, the concentration used in mouse cell experiments. We therefore utilized PXGL medium (Bredenkamp et al., 2019b), a more recently developed medium optimized for naive hiPSCs. PXGL eliminated the EOS-GFP-negative population and alleviated growth retardation caused by the NMT inhibitor (Figures 5A–5C). We then cultured hiPSCs with or without 0.1 μM NMT inhibitor for 2 weeks, resulting in a 2.5-fold increase in EOS-GFP signal with NMT inhibition (Figure 5C).

To further investigate the effect of the NMT suppression, we performed RNA-seq. In contrast to mESCs, where most naive markers were upregulated (Figure 1E), only partial induction of naive markers was observed in hiPSCs (Figure 5D). However, *KLF2* and *TBX3* showed substantial increases, with 5.6-fold and 3.3-fold upregulation, respectively (Figure 5D). While *KLF2* is expressed at low levels in naive hPSCs (Guo et al., 2016), its forced expression with *NANOG* has been reported to facilitate primed-to-naive conversion (Takashima et al., 2014; Theunissen et al., 2014), suggesting that its upregulation may mediate the effects of the NMT inhibitor. General pluripotency markers, such as *POU5F1* (encoding OCT3/4), *SOX2*, and *NANOG*, remained highly expressed, though *SOX2* and *NANOG* showed modest reductions (Figure 5D).

Unexpectedly, trophectoderm and primitive endoderm markers significantly increased with NMT inhibitor treatment (Figure 5D). These increases were absent in NMT1-deficient mESCs (Figure S1E), suggesting a response specific to hPSCs. To explore this further, we examined top-ranked markers of epiblast, trophectoderm, and primitive endoderm using single-cell RNA sequencing (scRNA-seq) data from human pre-implantation embryos (Petropoulos et al., 2016). This confirmed significant induction of trophectoderm and primitive endoderm markers, with stronger upregulation seen in the trophectoderm lineage (Figure 5E).

### NMT suppression expands hiPSC subpopulations expressing trophectoderm and primitive endoderm markers while co-expressing pluripotency markers, as identified by scRNA-seq

Bulk RNA-seq analysis (Figure 5) could not determine whether the increased expression of trophectoderm and primitive endoderm markers was due to expansion of specific cell populations or a global change across all cells. Recent scRNA-seq studies have shown that naive hiPSCs cultured in 5iLAF medium (Theunissen et al., 2014), a well-established naive-state medium, exhibit cellular heterogeneity, with subsets expressing trophectoderm, primitive endoderm, and eight-cell (8C)-stage markers (Moya-Jódar et al., 2023). The marker upregulation observed in our bulk RNA-seq data (Figure 5) suggests that NMT inhibition may enhance this heterogeneity. To investigate this, we performed scRNA-seq on hiPSCs cultured with or without 0.1 μM NMT inhibitor for 2 weeks.

**Figure 6:**
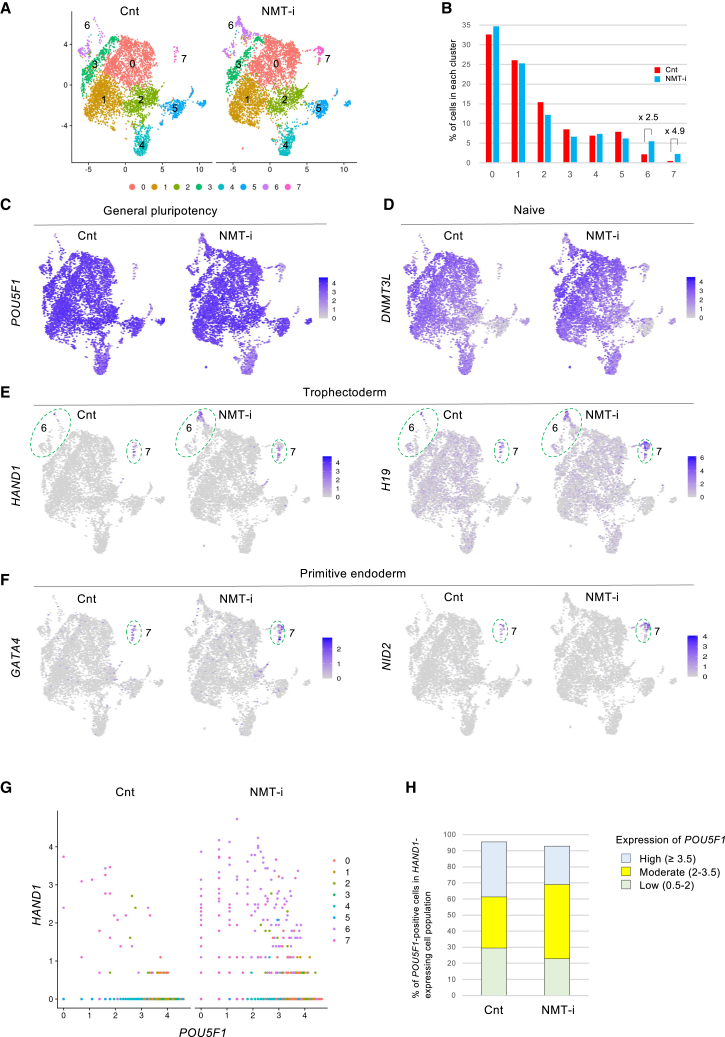
NMT suppression expands hiPSC subpopulations expressing trophectoderm and primitive endoderm markers while co-expressing pluripotency markers, as identified by scRNA-seq (A) Uniform Manifold Approximation and Projection (UMAP) plots of naive hiPSCs in the absence (Cnt, control: 5,360 cells) and presence (NMT-i: 5,506 cells) of the NMT inhibitor. (B) Cluster proportions from (A). (C–F) UMAPs showing expression of developmental markers. Clusters 6 and 7 are circled. (G) Scatterplots of *POU5F1* and *HAND1* co-expression, colored by clusters 0–7 as in (A). (H) Quantification of co-expression in (G), stratified by *POU5F1* levels.

Naive hiPSCs were grouped into clusters 0–7 (Figure 6A), with clusters 6 and 7 expanding in response to NMT inhibition (Figure 6B). Independent of NMT inhibition, general pluripotency markers such as *POU5F1*, *SOX2*, and *NANOG* were broadly expressed, though their levels were relatively low in cluster 7 (Figures 6C and S4A). Similarly, naive-state markers such as *DNMT3L*, *DPPA3*, and *PRDM14* were expressed at lower levels in this cluster (Figures 6D and S4A). In contrast, trophectoderm markers including *HAND1*, *H19*, *GATA2*, and *GATA3* were primarily expressed in cluster 7, with additional expression also noted in cluster 6 (Figures 6E and S4A). Therefore, as previously reported in 5iLAF medium, naive hiPSCs cultured in PXGL medium also contained subpopulations expressing trophectoderm markers. Expression of these markers was further enhanced by NMT inhibition (Figures 6E and S4A), supporting the notion that the increase in trophectoderm markers observed in bulk RNA-seq reflects subpopulation expansion, rather than a global effect. Subpopulations expressing primitive endoderm markers have also been reported in 5iLAF medium (Moya-Jódar et al., 2023). Likewise, primitive endoderm markers, such as *GATA4*, *GATA6*, *NID2*, and *COL4A1*, were expressed in cluster 7, and their expression was enhanced by NMT inhibition (Figures 6F and S4A).

Notably, most cells expressing trophectoderm or primitive endoderm markers also co-expressed the pluripotency marker *POU5F1*, with or without the NMT inhibitor (Figures 6G, 6H, S4B, and S4C). This co-expression resembles transitional states in human embryos (Petropoulos et al., 2016), suggesting that NMT inhibition may enhance developmental plasticity within the naive population. However, when we tested functional outcomes by performing blastoid formation (Kagawa et al., 2022; Yanagida et al., 2021; Yu et al., 2021) and trophectoderm differentiation assays (Guo et al., 2021; Io et al., 2021), no enhancement was observed under NMT inhibition (Figure S5).

A small fraction of naive hiPSCs express markers of 8C-stage human embryos (Mazid et al., 2022; Moya-Jódar et al., 2023; Taubenschmid-Stowers et al., 2022). These cells are referred to as 8C-like cells (8CLCs) and express totipotency-associated genes such as *ZSCAN4*, *DUXA*, and *TPRX1*. *ZSCAN4*-positive cells were found in cluster 6 (Figure S6A) but were distinct from trophectoderm-expressing population (Figure 6E). Subclustering of cluster 6 revealed that the trophectoderm-positive subset (6s1) expanded markedly (19.5-fold) by NMT inhibition, whereas 8CLC-associated subsets (6s2 and 6s3) did not increase (Figures S6B–S6D). Thus, NMT suppression does not induce 8CLCs.

These findings demonstrate that NMT inhibition induces the expression of trophectoderm and primitive endoderm markers in naive hiPSCs while maintaining co-expression of pluripotency markers. In contrast, NMT suppression in mouse cells primarily induces naive-state markers, suggesting intrinsic species-specific differences in the naive state.

## Discussion

In this study, we identified NMT as a novel regulator of the naive state, with distinct functions in mice and humans. NMT catalyzes the attachment of myristate, a 14-carbon fatty acid, to the N-terminal glycine residue of proteins (Yuan et al., 2020). The significance of myristoylation during early development is underscored by the embryonic lethality observed in *Nmt1*-knockout mice (Yang et al., 2005). Myristoylation increases protein hydrophobicity, promoting membrane targeting and clustering of signaling molecules, thereby activating diverse pathways (Yuan et al., 2020). Considering this observation, an attractive model to explain how NMT suppression enhances the naive state in mice is that it shields cells from external differentiation stimuli by reducing the density of signaling molecules at the plasma membrane. This concept resembles the mechanism by which 2i inhibitors stabilize the naive state (Ying et al., 2008). MEK, a key 2i target, acts as a signal transducer in the FGF2-dependent differentiation pathway. Thus, naive cells cultured in 2i are sequestered from a major differentiation stimulus (Ying et al., 2008). Our results indicated that 2i alone was inefficient in converting mouse cells from the primed to the naive state, as reported previously (Guo et al., 2009). In contrast, the addition of the NMT inhibitor enhanced the efficiency of this conversion (Figure 2G). Furthermore, the dome-shaped morphology of the colonies, a characteristic feature of naive cells, became more pronounced with the addition of the NMT inhibitor to 2i medium, indicating a non-overlapping effect between MEK and NMT inhibitors (Figure 1D). These observations suggest that signaling pathways other than the FGF2-MEK axis are targeted by the NMT inhibitor.

SRC, a known NMT substrate, promotes mESC differentiation (Meyn et al., 2005), and its inhibition stabilizes the naive state (Shimizu et al., 2012). Therefore, we initially hypothesized that SRC mediates the conversion of primed-state mEpiSCs into naive-state cells by NMT suppression. However, the SRC inhibitor did not enhance this conversion (Figure 4), suggesting that other Nmt substrates are involved. Advances in proteomics have identified more than 100 *N*-myristoylated proteins in HeLa cells (Thinon et al., 2014). Comparative profiling of naive and primed states may uncover novel pluripotency regulators.

Various roles other than plasma membrane targeting have been reported for myristoylation. A hydrophobic myristoyl moiety can alter protein folding and provide a novel interface for protein-protein interactions (Spassov et al., 2018). The myristoylation of proteasome components controls the shuttling of proteasome complexes between nucleus and cytoplasm, which regulates the degradation of misfolded proteins (Kimura et al., 2016). Several proteins involved in apoptosis are myristoylated following caspase-mediated cleavage, which can enhance or reduce the activity of each protein and influence the balance between cell death and survival (Martin et al., 2008). These or other unknown mechanisms may be involved in the regulation of pluripotency observed in our study.

Transcriptome analyses of naive hiPSCs treated with the NMT inhibitor revealed an upregulation of trophectoderm and primitive endoderm markers (Figures 5D, 5E, and 6), in contrast to the findings in mouse cells (Figure 1). Given that naive hPSCs naturally express these markers, and that we observed co-expression of the pluripotency marker *POU5F1* with these lineage markers in the presence of the NMT inhibitor (Figures 6G, 6H, S4B, and S4C), we hypothesized that this upregulation might reflect increased differentiation plasticity rather than a loss of pluripotency. Nonetheless, blastoid formation and trophectoderm differentiation assays showed no enhancement (Figure S5). Thus, the functional significance of this marker upregulation remains unclear and warrants further investigation.

We consider that there is room to improve the efficacy and specificity of the NMT inhibitor because our conditional *Nmt1*-knockout experiment gave superior results compared to the NMT inhibitor in terms of the induction level of naive cells from primed-state cells (Figures 2G and 3H). Recently, NMT has attracted increasing attention as a therapeutic target in cancers, and new NMT inhibitors are being developed accordingly (Yuan et al., 2020). The NMT inhibitor used in the present study (DDD85646) was originally developed as a lead compound to target NMT of *Trypanosoma brucei* (Frearson et al., 2010) and is therefore unlikely to be an optimal inhibitor for mammalian NMT. The newly developed inhibitors optimized for human NMT may regulate pluripotent stem cells more effectively.

## Methods

Full experimental details are available in the supplemental information.

### Cell line and cell culture

Experiments using hiPSCs, including blastoid formation assay, were approved by the institutional review board of Nara Medical University. The *Nmt1*-mutant mESC clone was obtained previously (Horie et al., 2011). The retroviral gene trap vector was inserted at the first intron of the *Nmt1* gene. The flanking sequence of the insertion site is 5′-ATCCCACGCTGGTCTCATTTGGACA-3'. mESCs, mEpiSCs, hiPSCs, and hESCs were cultured under standard conditions.

### Immunostaining

For immunostaining of OCT3/4, NANOG, and KLF4, cells were fixed in 4% paraformaldehyde in PBS for 10 min, permeabilized with 0.2% Triton X-100 for 10 min, and stained using a standard protocol with the following primary antibodies: anti-OCT3/4 mouse monoclonal antibody (1:300, clone c-10, Cat. Sc-5279, Santa Cruz Biotechnology), anti-NANOG rabbit polyclonal antibody (1:200, Cat. RCAB002P-F, ReproCELL), and anti-KLF4 rabbit monoclonal antibody (1:1,000, Cat. ab214666, Abcam). For SUSD2 staining, cells were incubated with allophycocyanin(APC)-conjugated anti-SUSD2 antibody (1:20, clone W5C5, Cat. 327401, BioLegend) in culture medium for 30 min and analyzed by flow cytometry.

### Generating chimeric mice and assessing germline transmission

All animal experiments were approved by the institutional review boards of Nara Medical University, Osaka University, and Kyoto University, and were performed in accordance with institutional guidelines. mEpi-iPSCs were injected into 8C-stage embryos or blastocysts derived from ICR or BDF1 mice. As the parental mEpiSCs originated from a female 129SV mouse, female chimeric mice with agouti coat color were selected and crossed with male C57BL/6J mice. Germline transmission was assessed based on the presence of agouti-colored progeny.

### Generating the conditional allele at the *Nmt1* locus

We conducted a standard gene targeting to flox the second exon of the WT allele of the *Nmt1*-heterozygous mESC clone. The targeting vector was constructed by a standard protocol using PCR primers listed in Table S1.

### Conversion between primed and naive states

mESCs carrying the floxed *Nmt1* allele were converted into mEpiSC-like primed-state cells using a published protocol (Guo et al., 2009). To induce the naive state, the *Nmt1* allele was deleted via 4HT-induced Cre activation, followed by culture in 2i/LIF medium.

### RT-qPCR

Expression levels of *Dppa3*, *Fgf5*, and *Actb* in mouse cells were quantified by real-time PCR using the LightCycler FastStart DNA Master SYBR Green I kit (Cat. 12239264001, Roche Diagnostics) using primers listed in Table S1. Gene expression in hESCs (KhES-1) was analyzed using the hESC RT^2^ Profiler PCR array (Cat. PAHS-081, QIAGEN).

### Microscopic analysis of myrVenus reporter localization

The expression vector of the myrVenus reporter (Rhee et al., 2006) was constructed as described in the supplemental information. The plasma membrane was stained with CellMask Deep Red plasma membrane stain (Cat. C10046, Thermo Fisher Scientific), and fluorescence signals were evaluated by line-plot analysis using the DeltaVision Elite system (Cytiva).

### Western blot analysis

To examine the effect of the *Nmt1* knockout on the localization of the myrVenus reporter, membrane and cytosol fractions were prepared from mESCs using Minute plasma membrane protein isolation and cell fractionation kit (Cat. SM-005, Invent Biotechnologies, Inc.) and analyzed by standard western blotting. Primary antibodies used were anti-GFP (1:2,500, Cat. 598, MBL International Corp.), anti-Na/K-ATPase (1:1,000, Cat. 3010, Cell Signaling Technology), and anti-GAPDH (1:3,000, Cat. 2118, Cell Signaling Technology). The amounts of myrVenus in each fraction were normalized to GAPDH for the cytosol fraction and Na/K-ATPase for the membrane fraction.

To assess the inhibitory effect of CGP77675 on FAK phosphorylation, western blotting was performed using anti-Phospho-FAK (Tyr925) (1:1,000, Cat. 3284, Cell Signaling Technology) and anti-total FAK (D2R2E) (1:1,000, Cat. 13009, Cell Signaling Technology). Phospho-FAK levels were normalized by total FAK.

### Bulk RNA-seq and scRNA-seq

Bulk RNA-seq libraries were prepared from total RNA using the TruSeq stranded mRNA library prep kit (Cat. 20020595, Illumina). scRNA-Seq libraries were generated using the Chromium Next GEM Single Cell 5′ Library and Gel Bead Kit v.2 (Cat. 1000263, 10× Genomics). Sequencing was performed on an Illumina NovaSeq 6000 platform in paired-end mode (2 × 101 nt). Details of the bioinformatic procedures are provided in the supplemental information. Gene expression levels in mESCs and hiPSCs are presented as FPKM values in Tables S2 and S3, respectively.

### Blastoid formation and trophectoderm differentiation assays

Blastoid formation from naive hiPSCs was performed according to the published protocol on an Elplasia 96-well plate (Cat. 4442, Corning), with modifications detailed in the supplemental information. Trophectoderm differentiation was carried out following the published protocol with or without BMP4.

### Statistical analysis

Student’s t test or Welch’s t test was used for comparisons between two groups. Tukey-Kramer test was applied for multiple comparisons across all groups, and Dunnett’s test for comparisons to a control.

## Resource availability

### Lead contact

Requests for further information and resources should be directed to and will be fulfilled by the lead contact, Kyoji Horie (k-horie@naramed-u.ac.jp).

### Materials availability

The *Nmt1*-homozygous mutant mESC line Nmt1-K1 and its parental mESC line vdR2-4 have been deposited in the Japanese Collection of Reseach Bioresources (JCRB) Cell Bank (https://cellbank.nibn.go.jp/english) under the accession numbers AyuK8A06 and JCRB1658, respectively.

### Data and code availability

RNA-seq data have been deposited in the DNA Data Bank of Japan (DDBJ) BioProject database under accession number PRJDB18034. This paper does not report any original code.

## Acknowledgments

We acknowledge the 10.13039/100006363NGS core facility of the Genome Information Research Center at the Research Institute for Microbial Diseases of 10.13039/501100004206Osaka University for supporting RNA-seq experiments. We thank Dr. Paul Wyatt at the Drug Discovery Unit, School of Life Sciences, 10.13039/100008890University of Dundee, for providing DDD85646 (prepared with the support of 10.13039/100010269Wellcome Trust grant WT 077705). We also thank Dr. Paul Tesar for the mEpiSCs; Dr. Kat Hadjantonakis for the myrVenus reporter vector; Dr. Masaru Okabe for supporting the generation of chimeric mice; and Drs. Daisuke Okuzaki, Miwa Sasai, and Masahiro Yamamoto for their assistance with the RNA-seq experiments. This work was supported by Grants-in-Aid for Scientific Research from the 10.13039/501100001700Ministry of Education, Culture, Sports, Science and Technology of Japan (JP16H04683, JP18K19275, and JP20H03174 for K.H.); 10.13039/501100001695JST 10.13039/501100009023PRESTO (K.H.); 10.13039/100009619AMED (JP20bm0704035 for Y.T.); and the Cooperative Research Program (Joint Usage/Research Center program) of Institute for Life and Medical Sciences, 10.13039/501100005683Kyoto University (K.H.). This work was also supported in part by the research grant from the 10.13039/100007449Takeda Science Foundation (K.H.), 10.13039/100007428Naito Foundation (K.H.), and 10.13039/501100005927Daiichi Sankyo Foundation of Life Science (K.H.).

## Author contributions

Conceptualization, K.H. and J.T.; methodology, J.Y., H.M., and K.H.; investigation, J.Y., H.W., K.Y., T.N., A.I., Y.K., H.A., H.S., Y.T., H.M., G.K., and K.H.; writing – original draft, K.H.; resources, S.O., H.N., and Y.T.; supervision, H.A., A.U., H.S., H.M., J.T., and K.H.; project administration, J.T. and K.H.; funding acquisition, Y.T., H.M., and K.H.

## Declaration of interests

The authors declare no competing interests.

## Declaration of generative AI and AI-assisted technologies in the writing process

During the preparation of this work, the authors used ChatGPT in order to improve language and readability. After using this tool/service, the authors reviewed and edited the content as needed and take full responsibility for the content of the publication.

## Supplemental information

Document S1. Figures S1–S6, Table S1, and supplemental methods

Table S2. RNA-seq analysis of Nmt1-mutant and wild-type mESCs

Table S3. RNA-seq analysis of naive hiPSCs with or without NMT inhibitor

Document S2. Article plus supplemental information
