# Supplementary material for: Inhibition of N-myristoyltransferase in pluripotent stem cells promotes the naive state in mice and elicits trophectoderm and primitive endoderm markers in humans
Source: Stem Cell Reports. 2025 Aug 28;20(9):102610. doi: 10.1016/j.stemcr.2025.102610 (PMC12447342; doi:10.1016/j.stemcr.2025.102610)
Supplement: Document S1. Figures S1–S6, Table S1, and supplemental methods [file mmc1.pdf]

Stem Cell Reports, Volume *20*

Supplemental Information

Inhibition of N-myristoyltransferase in pluripotent stem cells promotes the naive state in mice and elicits trophectoderm and primitive endoderm markers in humans

Junko Yoshida, Hitomi Watanabe, Kaori Yamauchi, Takumi Nishikubo, Ayako Isotani, Satoshi Ohtsuka, Hitoshi Niwa, Yuki Kawamoto, Hidenori Akutsu, Akihiro Umezawa, Hirofumi Suemori, Yasuhiro Takashima, Hideo Matsuda, Gen Kondoh, Junji Takeda, and Kyoji Horie

Inhibition of N-myristoyltransferase in Pluripotent Stem Cells Promotes the Naive State in Mice and Elicits Trophectoderm and Primitive Endoderm Markers in Humans

Junko Yoshida, Hitomi Watanabe, Kaori Yamauchi, Takumi Nishikubo, Ayako Isotani, Satoshi Ohtsuka, Hitoshi Niwa, Yuki Kawamoto, Hidenori Akutsu, Akihiro Umezawa, Hirofumi Suemori, Yasuhiro Takashima, Hideo Matsuda, Gen Kondoh, Junji Takeda, and Kyoji Horie

This Supplementary File includes:

- Supplementary Figures 1-6 and their legends
- Supplementary Table 1. PCR primers used in this study
- Supplemental Experimental Procedures

The following Supplementary Tables are provided in Excel format:

- Supplementary Table 2. RNA-seq analysis of *Nmt1*-mutant and wild-type mESCs, related to Figure 1E
- Supplementary Table 3. RNA-seq analysis of naive hiPSCs with or without NMT inhibitor, related to Figure 5D

Supplementary Figure 1

A

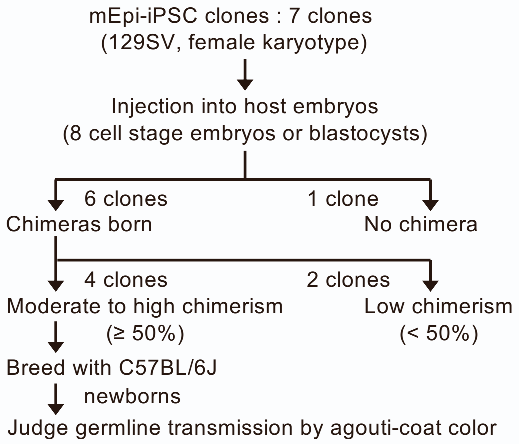

mEpiSC-iPSC clone	Coat color of progeny agouti : black
1	1:9
2	2:7
3	1:10
4	1:9

C

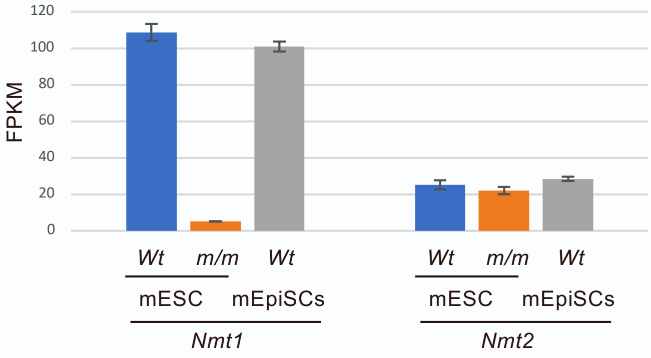

D

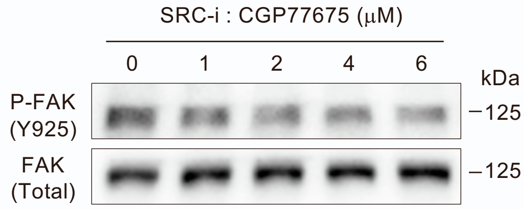

B

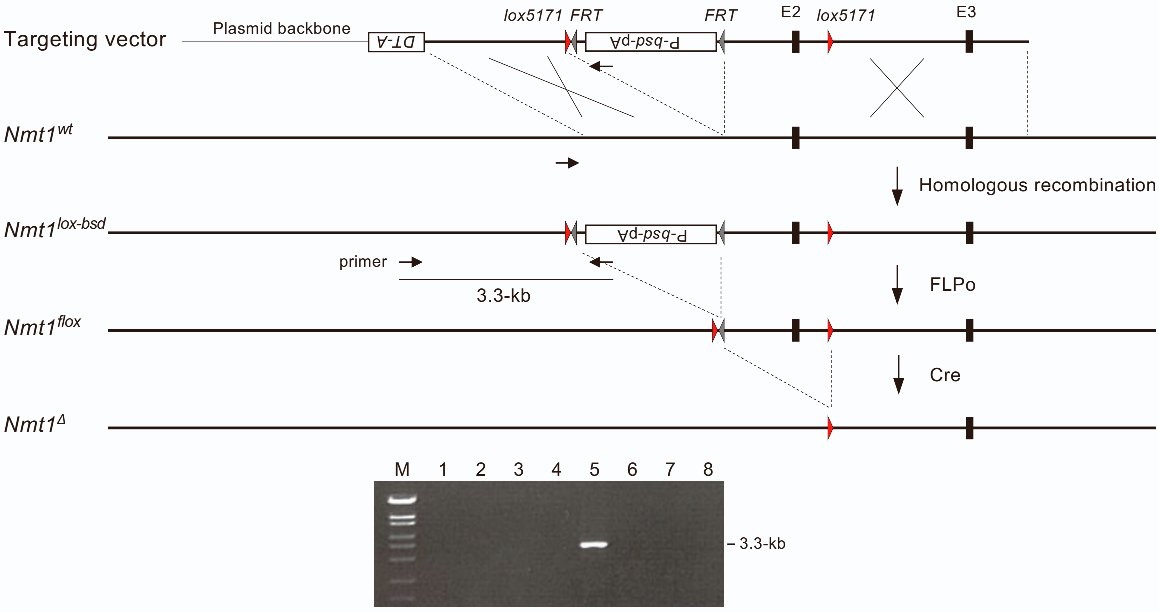

E

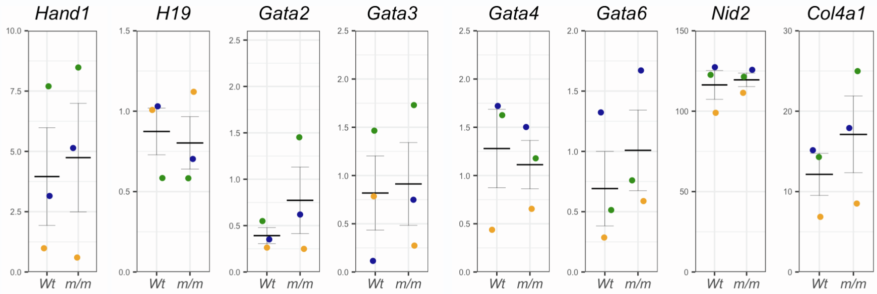

Supplementary Figure 1. Effect of NMT suppression on mouse cells, related to Figures 1-5.

(A) Generation of chimeric mice from mEpi-iPSC clones and evaluation of germline transmission rate, related to Figure 2J.

(B) Generation of the conditional allele at the *Nmt1* locus by gene targeting, related to Figures 3A and 3B. Gene targeting was conducted to the wild-type *Nmt1* allele (*Nmt1*^{wt}) of the heterozygous gene-trapped clone (*Nmt1*$^{m/wt}$). Homologous recombination between the targeting vector and the *Nmt1*^{wt} allele was screened by PCR as shown in the gel image. FLPo-mediated excision of the P-*bsd*-pA cassette was identified by blasticidin S-sensitivity of the single cell-derived clones. Detection of Cre-mediated recombination was performed by 4-hydroxytamoxifen administration as shown in Figures 3A and 3B. *DT-A*, diphtheria toxin A fragment; P, CAG promoter; *bsd,* blasticidin S deaminase gene; pA, bovine growth hormone polyadenylation signal; E, exon; M, size marker (λ/StyI digest).

(C) Expression levels of *Nmt1* and *Nmt2* in mESCs and mEpiSCs as determined by RNA-seq. Data are presented as the mean $\pm$ SEM of FPKM (fragments per kilobase per million mapped reads) from three independent replicates.

(D) Western blot analysis of replicate 2 from Figure 4A, demonstrating the dose-dependent inhibitory effect of the SRC inhibitor on FAK-phosphorylation at Y925 in mEpiSCs. Quantification of band intensities is shown in Figure. 4A.

(E) Effect of *Nmt1* disruption on mouse orthologs corresponding to the human trophectoderm and primitive endoderm marker genes shown in Figure 5D. No significant difference was observed in the expression of mouse markers shown here between *Wt* and *Nmt1*$^{m/m}$ based on statistical analysis. Note that the data for *Gata4* and *Gata6* are the same as those shown in Figure 1E.

Supplementary Figure 2

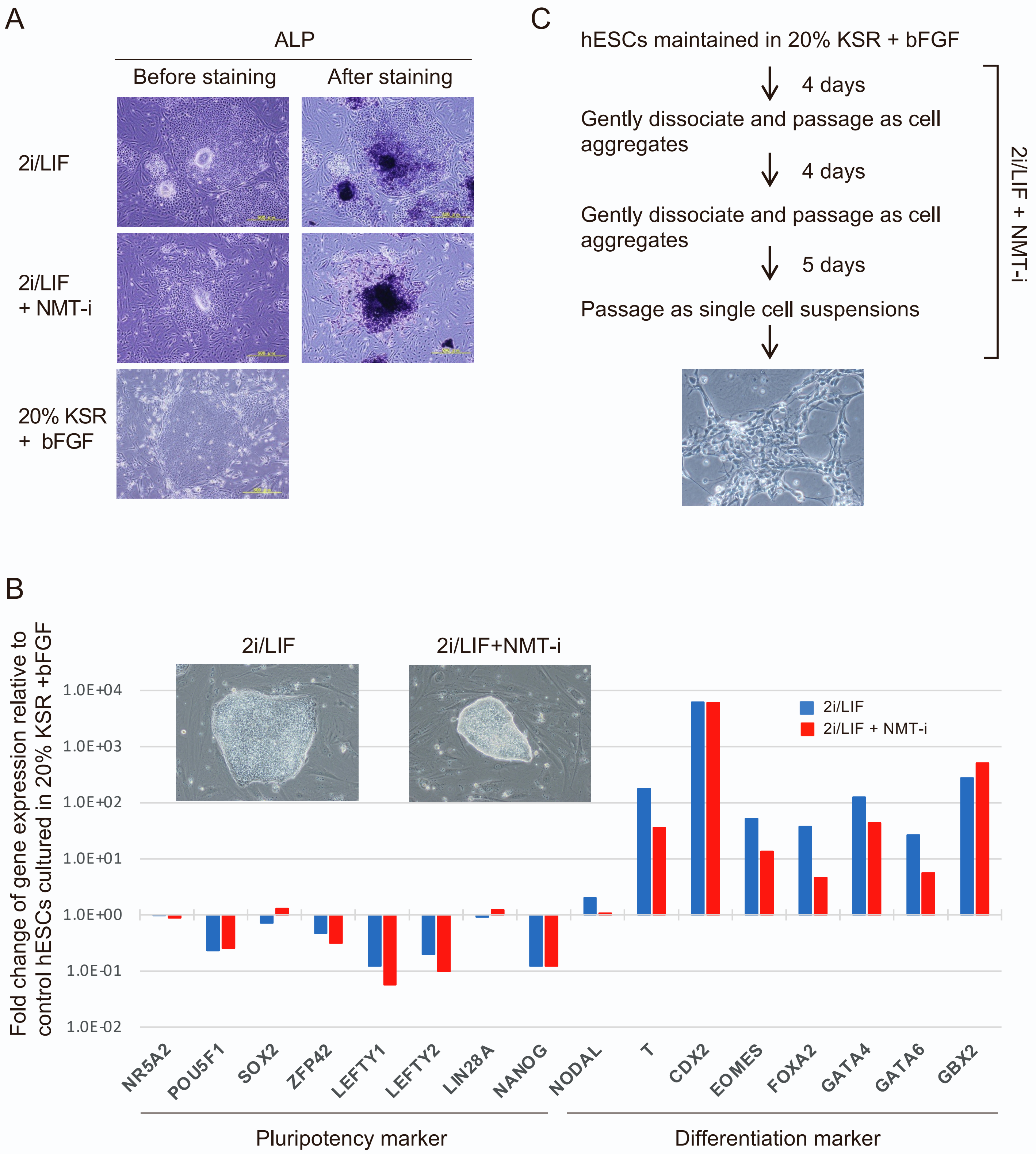

Supplementary Figure 2. Culture condition of 2i/LIF + NMT inhibitor does not support the undifferentiated state of human pluripotent stem cells.
(A) ALP-staining of hiPSCs at passage 2 in 2i/LIF + NMT inhibitor.
(B) Gene expression in hESCs after 4 day-culture in 2i/LIF or 2i/LIF + NMT inhibitor relative to KSR +bFGF.
(C) Morphology of hESCs after long-term culture in 2i/LIF + NMT inhibitor.

Supplementary Figure 3

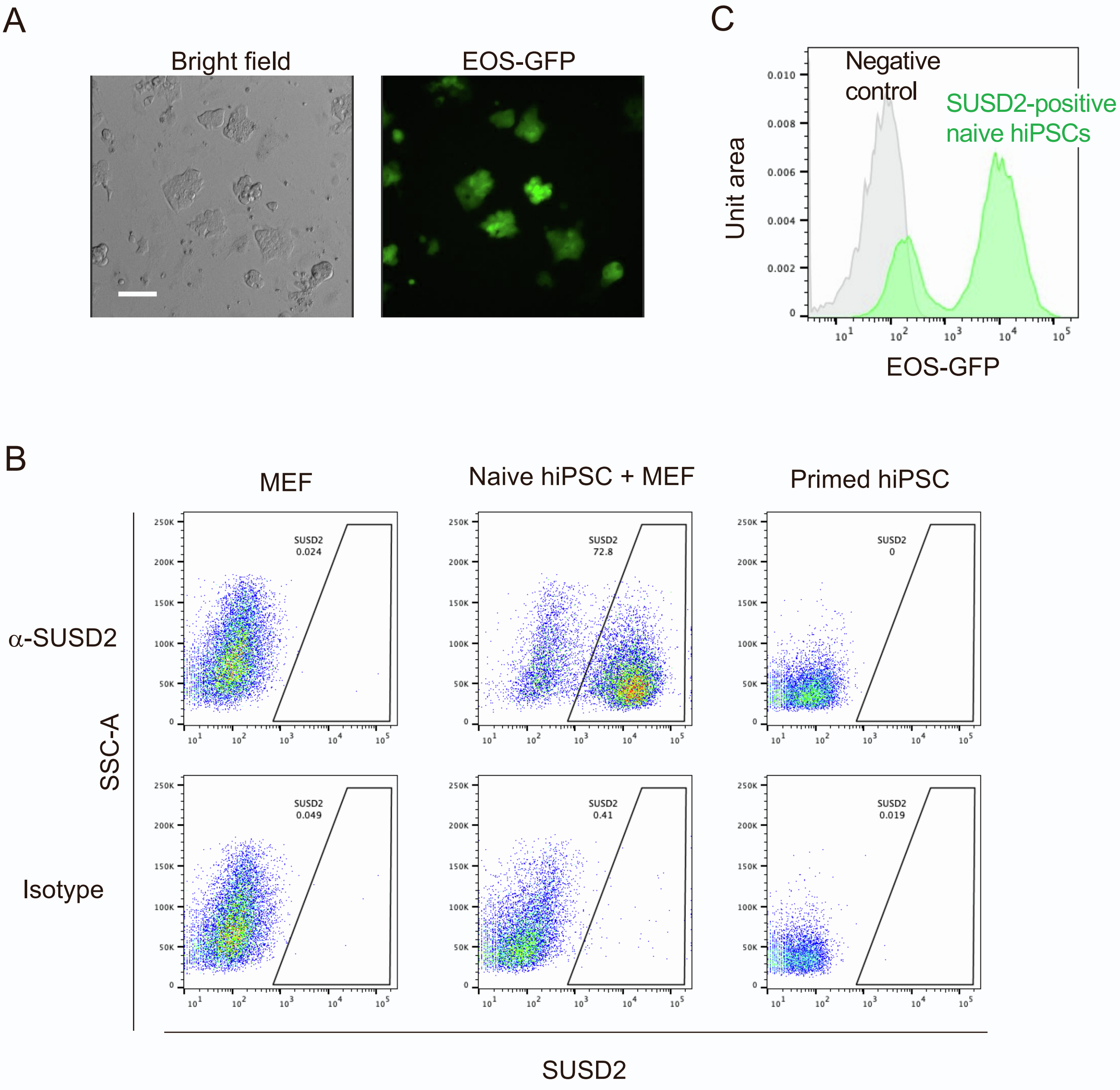

Supplementary Figure 3. Naive hiPSCs generated using t2iLGö medium, related to Figure 5.

(A) Morphology of naive hiPSCs and EOS-GFP reporter expression in t2iLGö medium. Scale bar: 100 μm.

(B) Expression of naive-state marker SUSD2. MEF feeders could be excluded from the naive hiPSC culture by gating the expression of SUSD2.

(C) The expression of EOS-GFP in the SUSD2-positive population shown in (B).

Supplementary Figure 4

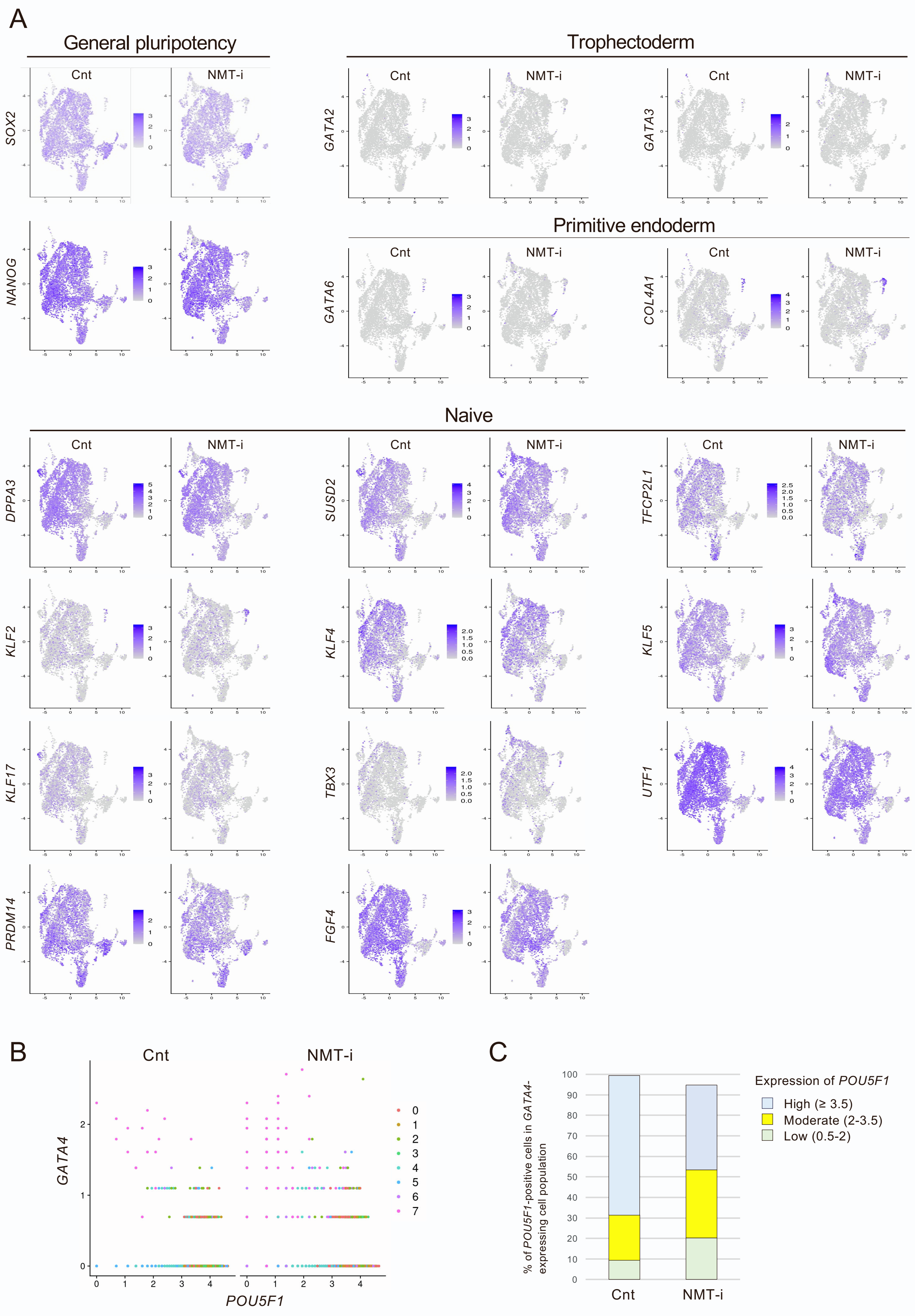

Supplementary Figure 4. Effect of NMT inhibitor on the expression of developmental markers in naive hiPSCs analyzed by scRNA-seq, related to Figure 6.

(A) UMAPs showing the expression of developmental markers.

(B) Scatter plots of *POU5F1* and *GATA4* co-expression, colored by clusters 0–7 as in Figure 6A.

(C) Quantification of co-expression shown in (B), stratified by *POU5F1* expression levels.

Supplementary Figure 5

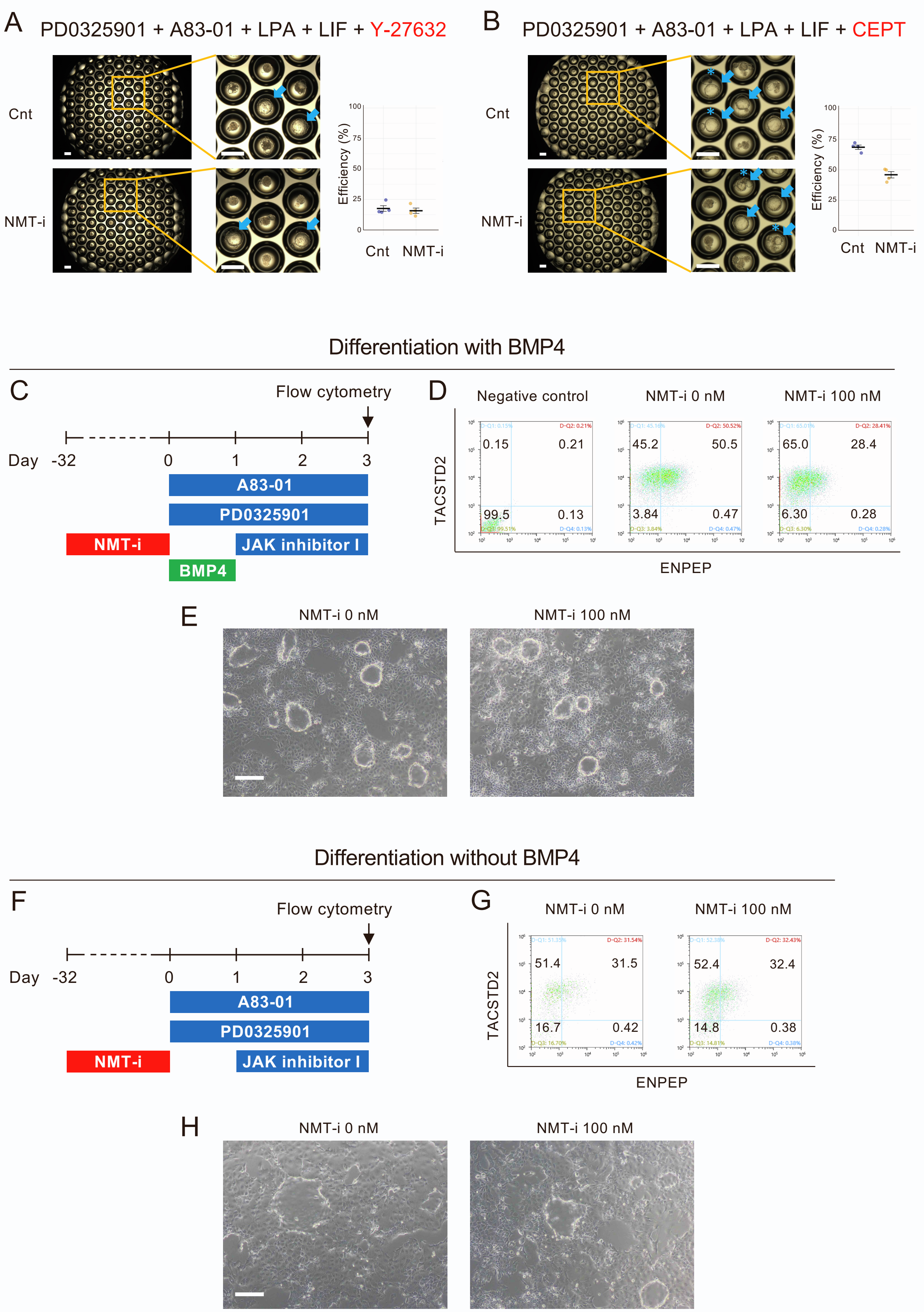

Supplementary Figure 5. Effect of the NMT inhibitor on blastoid formation and trophectoderm differentiation, related to Figure 6.

(A, B) Blastoid formation assay. Phase-contrast images of human blastoids formed in an Elplasia microwell plate (Corning) and the efficiency of blastoid formation judged by cyst formation. To enhance the viability of cell aggregates, Y-27632 was used in (A), while the CEPT cocktail was used in (B). Representative images of an Elplasia well, each containing approximately 90 microwells, are shown on the left. Blue arrows indicate microwells in which blastoid formation occurred. Multi-cystic structures were frequently observed in the presence of the CEPT cocktail (B), as indicated by asterisks. The efficiency of blastoid formation from four wells is presented as mean ± SEM. NMT-i, NMT inhibitor; Cnt, control without NMT-i. Scale bar: 400 μm.

(C-H) Trophectoderm differentiation assay. (C, F) Schematic representation of the protocol for trophectoderm induction with (C) and without (F) BMP4. The NMT inhibitor was added prior to the differentiation induction but omitted during the differentiation process. (D, G) Flow cytometry analysis of trophectoderm markers on day 3. Gating for the expression of ENPEP and TACSTD2 was established based on unstained cells differentiated in the presence of BMP4, as shown in (D), and the same gating was applied in (G). (E, H) Phase-contrast image of differentiated cells on day 3. Scale bar: 500 μm.

Supplementary Figure 6

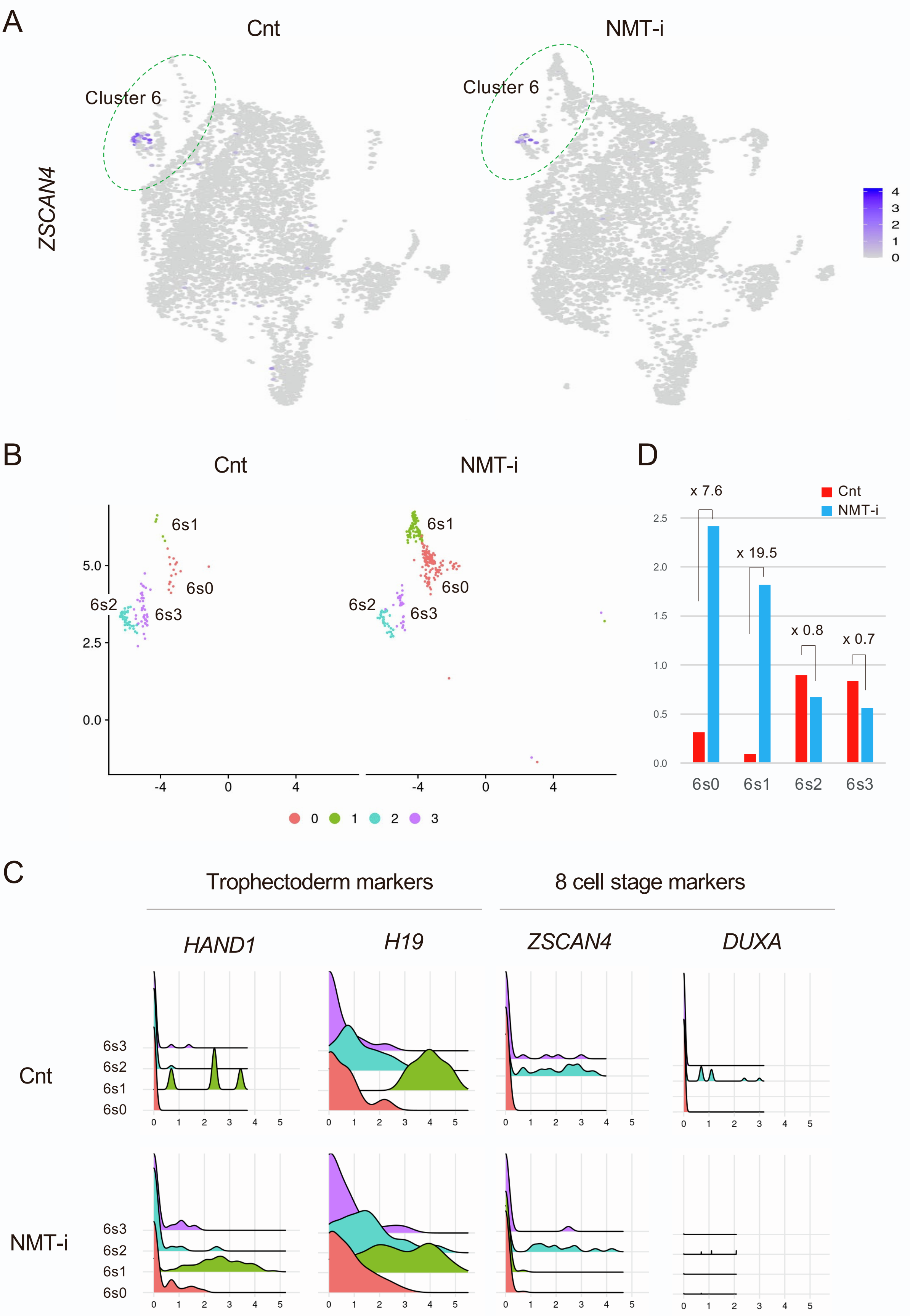

Supplementary Figure 6. Analysis of eight-cell-stage marker expression by scRNA-seq, related to Figure 6.

(A) UMAP visualization showing the expression of *ZSCAN4*.

(B) Subclustering of cluster 6.

(C) Ridge plot showing the expression patterns of trophectoderm and eight-cell-stage markers.

(D) Proportions of cell populations in each subcluster identified in (B).

Supplementary Table 1. PCR primers

Construction of the *Nmt1* -targeting vector	
Nmt1-L-Upp1	cgtttaaacggcgcgccAGCTCCAACTCCAGTACGTGCTGTCTATTC
Nmt1-L-Low1	cttcgtataatgtgtactatacgaagttatGTTAACTGGTCAGGGGCTAGGTCGAGGCAC
Nmt1-L-Upp2	cttcgtatagtacacattatacgaagttatTGACGAGTATTTTGACTGGTTTGCTCTTCT
Nmt1-L-Low2	taccgtcgaccttaattaaATCCCACGCTGGTCTCATTTGGACATTTTA
Nmt1-S-Upp1	agttctagagcggccgcCACGGCCTTGCTGTGAGAGCACGTGGGAGG
Nmt1-S-Low1	cactgctcgacatttaaatGTAGCGGATGCTTCCTGCCTCCCAAGGACA
Screening of the *Nmt1* -targeted clones	
Nmt1-scn1	GCCTCCCAAGGACAGATTCCATCTCATTCT
bsd3-1	AATTGCTGCCCTCTGGTTATGTGTGGGAGG
Detection of the Cre/*loxP* -mediated deletion of the *Nmt1* exon 2 region	
Nmt1-Flpo-Scn-F1	TTTTACATTGTCCCCACCCCAGCCT
Nmt1-Flpo-Scn-R1	TTAGGCTGCTTTTCCCCCAGTAAGT
Nmt1-Cre-Scn-R1	CATACTGTGCTGTCAATGTACTGTG
qRT-PCR	
mFgf5-RP-F3	AAGTAGCGCGACGTTTTCTTC
mFgf5-RP-R3	CTGGAAACTGCTATGTTCCGAG
Dppa3-F1	GAGGACGCTTTGGATGATACAGACG
Dppa3-R1	CAACAAAGTGCGGACCCTTCTCTTG
Actb-F1	CAGGGTGTGATGGTGGGAATGGGTCAGAAG
Actb-R1	TACGTACATGGCTGGGGTGTTGAAGGTCTC

SUPPLEMENTAL EXPERIMENTAL PROCEDURES

Cell Line and Cell Culture

mESCs were cultured either in serum-containing medium or serum-free 2i medium depending on the purpose of the experiment. The serum-containing medium was composed of KnockOut DMEM (Cat. 10829018, Thermo Fisher Scientific) supplemented with 20% fetal bovine serum, non-essential amino acids (Cat. 11140050, Thermo Fisher Scientific), penicillin-streptomycin-glutamine (Cat. 10378016, Thermo Fisher Scientific), 0.1 mM of 2-mercaptoethanol (Cat. M3148, Sigma) and 1,000 U/ml of leukemia inhibitory factor (LIF) (Cat. ESG1107, Merck Millipore), and mitomycin C (MMC)-treated MEFs were used as feeder cells. The serum-free 2i medium was composed of N2B27 (Guo et al., 2017) supplemented with 1 μM of MEK inhibitor PD0325901 (Cat. Axon1408, Axon Medchem) and 3 μM of GSK3 inhibitor CHIR99021 (Cat. Axon1386, Axon Medchem). We routinely added LIF to the serum-free medium (2i/LIF), except in the experiment shown in Fig. 1D.

mEpiSCs were cultured in DMED/F12 (Cat. 11320033, Thermo Fisher Scientific) supplemented with 20% KnockOut Serum Replacement (KSR) (Cat. 10828028, Thermo Fisher Scientific), non-essential amino acids, sodium pyruvate, 0.1 mM of 2-mercaptoethanol, 5 ng/ml of bFGF (Cat. 16100102, Katayama Chemical Industries) and 10 ng/ml of activin A (Cat. 120-14, PeproTech). MMC-treated MEFs were used as feeder cells.

Naive-state hiPSCs were established from the primed-state adipocyte-derived hiPSCs (Takashima et al., 2014). We first introduced the EOS-GFP reporter vector (Hotta et al., 2009) into the primed-state hiPSCs and then induced conversion to the naive state by the protocol previously described (Guo et al., 2017). Naive hiPSCs were maintained in t2iLGö or PXGL media. t2iLGö medium consists of N2B27 (Ndiff227; Cat. Y40002, Takara Bio) with 1 μM of PD0325901 (Cat. 4192, Tocris), 1 μM of CHIR99021 (Cat. SML1046, Sigma-Aldrich), 10 ng/mL of recombinant human LIF (Cat. 300-05, Peprotech), and 2 μM of Gö6983 (Cat. 2285, Tocris), as previously described (Takashima *et al.*, 2014). Naive hiPSCs were passaged every 3-5 days using Accutase (Cat. A6964, Sigma-Aldrich). PXGL medium consists of N2B27 with 1 μM of PD0325901, 2 μM of XAV939 (Cat.X3004, Sigma-Aldrich), 2 μM of Gö6983, and 10 ng/mL of recombinant human LIF (Cat. 300-05, Peprotech), as previously described (Bredenkamp et al., 2019).

The primed-state hiPSC line MRC5iPS was generated from the human fetal lung fibroblast cell line MRC-5 (Jacobs et al., 1970) by retroviral transduction of reprogramming factors (*OCT3/4*, *SOX2*, *KLF4*, *c-MYC*) (Takahashi et al., 2007). ALP activity was detected with VECTOR Red Alkaline Phosphatase Substrate Kit I (Cat. SK-5100, Vector Laboratories) according to the manufacturer's instructions.

The primed-state hESC line KhES-1 was maintained as previously described (Suemori et al., 2006). To test the effect of the NMT inhibitor DDD85646 (Frearson et al., 2010) (provided by Dr. Paul Wyatt, University of Dundee, UK) on prime-state hESC, KhES-1 was passaged in 2i/LIF with or without the inhibitor and analyzed for gene expression.

For real-time PCR analysis and bulk RNA-seq of mESCs, MEF feeder cells were removed by plating cells on a gelatin-coated dish for 30 min during the passaging and collecting unattached cells. For real-time PCR analysis of the hESCs, MEF feeder cells were removed by separating them from clumps of hESCs under gravity sedimentation during the passaging. For scRNA-seq of hiPSCs, MEF feeder cells were not physically removed during cell culture; instead, their contributions were computationally excluded, as described later.

Isolation of *Nmt1*-revertant Clones

Nmt1-homozygous mESCs were transfected with pCAGGS-FLPo-IRESpuro (Kranz et al., 2010) using TransFast (Cat. E2431, Promega) to excise the *FRT*-flanked gene trap cassette. Three days after

transfection, mESCs were sparsely plated on MMC-treated MEFs for single cell cloning. One week later, single cell-derived mESC colonies were picked and divided into three culture conditions: (1) with G418 (Geneticin; Cat. 10131027, Thermo Fisher Scientific), (2) with puromycin (Cat. P7255, Sigma-Aldrich), and (3) without drug selection. The parental *Nmt1*-homozygous mESC clone expresses the neomycin-resistance gene and the puromycin-resistance gene from each allele. Therefore, reversion of both alleles confers sensitivity to both G418 and puromycin.

Immunostaining

For the immunostaining of OCT3/4, NANOG and KLF4, cells were fixed with 4% paraformaldehyde (Cat. 02890-45, Nacali Tesque) in PBS for 10 min, permeabilized with 0.2% Triton X-100 (Cat. 35501, Nacali Tesque) for 10 min, and subjected to blocking with 1% bovine serum albumin (BSA) (Cat A5611, Sigma-Aldrich) in PBS for 20 min. The following primary antibodies were used: anti-OCT3/4 mouse monoclonal antibody (1:300, clone c-10, Cat. Sc-5279, Santa Cruz Biotechnology), anti-NANOG rabbit polyclonal antibody (1:200, Cat. RCAB002P-F, ReproCELL), and anti-KLF4 rabbit monoclonal antibody (1:1000, Cat. ab214666, Abcam). Alexa Fluor 488-conjugated goat anti-mouse IgG (Cat. A-11001, Thermo Fisher Scientific) was used for OCT3/4 and Alexa Fluor 594-conjugated goat anti-rabbit IgG (Cat. A-11012, Thermo Fisher Scientific) for NANOG and KLF4 as a secondary antibody, respectively, and DAPI (Cat. 62248, Thermo Fisher Scientific) was used for counterstaining when needed. For the immunostaining of SUSD2, the cells were incubated with APC-conjugated anti-SUSD2 antibody (1:20, clone W5C5, Cat. 327401, BioLegend) for 30 min in culture medium. The cells were washed three times with PBS and analyzed by FACSAria II (Becton, Dickinson and Company) or SH800 (SONY).

Converting mEpiSCs to Naive-state mEpi-iPSCs

mEpiSCs (2×10^4) were plated onto MMC-treated MEFs in N2B27-based medium supplemented with bFGF and activin A. The next day (day 0), the medium was changed to an N2B27-based 2i/LIF medium with or without the NMT inhibitor (DDD85646) or the SRC inhibitor (CGP77675) (Cat. 21089, Cayman Chemical). On day 2, the cells were passaged at 1:20 onto MEFs in the same medium. On day 7, the cells were passaged at 1:100 onto MEFs in 2i/LIF medium without inhibitors. On day 15, the number of dome-shaped colonies were counted.

Generating Chimeric Mice and Assessing Germline Transmission

After generating mEpi-iPSCs from mEpiSCs in serum-free 2i/LIF medium, we cultured them in serum-containing medium on MMC-treated MEFs for several days and injected them into eight-cell-stage embryos or blastocysts. We used ICR or BDF1-derived embryos as a host. As the parental mEpiSCs were derived from a female 129SV mouse strain, we selected female agouti-colored chimeric mice and crossed them with male C57BL/6J mice to test germline transmission. The germline transmission was assessed by the agouti coat-color of the progeny.

Generating the Conditional Allele at the *Nmt1* Locus

To conduct a conditional knockout of the *Nmt1* gene, we performed gene targeting and floxed the second exon of the *Wt* allele of the *Nmt1*-heterozygous mESC clone that we previously obtained by gene trapping (Horie et al., 2011). The targeting vector was constructed as follows, and the PCR primer sequences are listed in Supplementary Table 1. We first PCR-amplified a genomic fragment of the first intron of the *Nmt1* gene, using primer pairs Nmt1-S-Upp1 and Nmt1-S-Low1, using the genomic DNA of the mESC line KY1.1 (Yagita et al., 2010) as a template, digested with NotI and SwaI, and cloned into the NotI-SwaI site of the pMulti-Lox5171-FRT-CAG-bsd-pA-FRT (unpublished), which contains the *FRT*-flanked blasticidin S deaminase expression cassette and a

single copy of the *lox5171* site at this cloning site, resulting in the pMulti-Lox5171-FRT-CAG-bsd-pA-FRT-5HR. We next amplified the *Nmt1* genomic region spanning from the first intron to the second intron, using the primers Nmt1-L-Upp2 and Nmt1-L-Low2, and the genomic region spanning from the second intron to the third intron, using the primers Nmt1-L-Upp1 and Nmt1-L-Low1. These fragments have overlapped sequences containing the *lox5171* site introduced by PCR primers. We therefore conducted fusion PCR, using the mixture of these fragments as a template, and using primers Nmt1-L-Upp1 and Nmt1-L-Low2. The fused fragments were digested with AscI and PacI and cloned into the AscI-PacI site of the pMulti-Lox5171-FRT-CAG-bsd-pA-FRT-5HR, resulting in the targeting vector pMulti-Lox5171-FRT-CAG-bsd-pA-FRT-5HR-3HR. The targeting vector was linearized with AscI, and 25 μg of the targeting vector was transfected into 1×10^7 of *Nmt1*-heterozygous cells (Horie *et al.*, 2011) by electroporation (240 V, 500 μF) with Gene Pulser II (Bio-Rad). One week later, blasticidin S-resistant clones were picked and screened for homologous recombinants by primers Nmt1-scn1 and bsd3-1. Targeted clones were transfected with pCAGGS-FLPo-IRESpuro to remove the bsd cassette and generate the floxed *Nmt1* allele. The parental mESC line contained the *ERT2-iCre-ERT2* gene at the *Rosa26* locus (Horie *et al.*, 2011). Therefore, the conditional knockout of the *Nmt1* gene was achieved by treating cells with 4-hydroxytamoxifen (4HT) (Cat. H6278, Sigma-Aldrich). To confirm that the conditional allele was correctly generated, we treated mESCs with 1 μM of 4HT overnight and analyzed Cre-mediated recombination by PCR, using primers Nmt1-Flpo-Scn-F1 and Nmt1-Flpo-Scn-R1 for detecting the undeleted allele, and primers Nmt1-Flpo-Scn-F1 and Nmt1-Cre-Scn-R1 for detecting the deleted allele. We also conducted PCR by mixing all three primers in order to suppress the amplification of MEF-derived genomic DNAs that could not be eliminated by plating on a gelatin-coated dish.

Converting mESCs to mEpiSC-like Primed-state Cells

mESCs carrying the floxed *Nmt1* allele were converted into mEpiSC-like primed-state cells according to the published protocol (Guo et al., 2009). Briefly, mESCs were cultured in N2B27 medium supplemented with 12 ng/ml of bFGF and 20 ng/ml of activin A on a dish coated with fibronectin (Cat. 354008, Corning), and passaged at every 3–5 days. The morphology of the cell clusters became gradually flatter. We analyzed the primed-state marker *Fgf5* and the naive state marker *Dppa3* by qRT-PCR at passage nine to confirm conversion into the primed state.

Converting mEpiSC-like Primed-state Cells to Naive-state mEpi-iPSCs by the Conditional Knockout of *Nmt1*

mEpiSC-like primed-state cells were plated onto MMC-treated MEFs in N2B27-based medium supplemented with 12 ng/ml of bFGF and 20 ng/ml of activin A and treated with 1 μM of 4HT for 12 hours to induce the Cre-mediated deletion of the *Nmt1* allele. The same amount of ethanol was added to the medium as a mock. The cells were maintained in the same N2B27/bFGF/activin A medium for another five days to reduce the intracellular concentration of NMT1 protein. Then, the cells were plated onto MMC-treated MEFs in the same medium at the concentration of 2×10^4 cells per well. The next day (day 0), the medium was changed to 2i/LIF medium to induce conversion to the naive state. On day 7, the cells were passaged at 1:70 onto MEFs. On day 15, the cells were stained for ALP activity, using VECTOR Red Alkaline Phosphatase Substrate Kit I (Cat. SK-5100, Vector Laboratories), according to the manufacturer's instructions, and the number of ALP-positive colonies were counted.

Quantitative RT-PCR (qRT-PCR)

To quantify gene expression in the mouse cells, the total RNA was extracted with RNeasy Plus Mini Kit (Cat. 74136, Qiagen) and reverse-transcribed with SuperScript III (Cat. 18080044, Thermo Fisher

Scientific), using random primers (Cat. C1181, Promega). The expression levels of mRNAs encoding *Dppa3*, *Fgf5*, and *Actb* were quantified by real-time PCR, using the LightCycler FastStart DNA Master SYBR Green I kit (Cat. 12239264001, Roche Diagnostics) on the LightCycler (Roche Diagnostics). The primer pairs are listed in Supplementary Table 1. The amplification conditions for *Dppa3* and *Fgf5* were 95 °C for 10 min for one cycle, followed by 40 cycles of denaturation at 95 °C for 10 sec, annealing at 56 °C for 5 sec and extension at 72 °C for 20 sec. The amplification conditions for *Actb* were the same except that the annealing temperature was 55 °C. The quantity of each transcript was measured from a standard curve, and the amounts of *Dppa3* and *Fgf5* transcript were normalized to *Actb* transcript levels.

To quantify gene expression in hESCs (KhES-1), the total RNA was extracted with RNeasy Micro Kit (Cat. 74004, Qiagen) and reverse-transcribed with an RT^2 First Strand Kit (Cat. 330404, Qiagen). The expression levels of the mRNAs were quantified using the Human Embryonic Stem Cell RT^2 Profile™ PCR Array (Cat. PAHS-081, Qiagen) and RT^2 SYBR Green qPCR Master Mix (Cat. 330504, Qiagen). All procedures followed the manufacturer's instructions.

Microscopic Analysis of myrVenus Reporter Localization

The myrVenus reporter (Rhee et al., 2006) was cloned into the piggyBac transposon vector (Cadinanos and Bradley, 2007) under the control of the CAG promoter (Niwa et al., 1991) and with the IRES-bsd selection cassette. This vector was introduced into mESCs by co-transfecting the piggyBac expression vector mPB (Cadinanos and Bradley, 2007), using TransFast transfection reagent, and selected by 30 µg/ml of blasticidin S (Cat. KK-400, Kaken Pharmaceutical). We stained the plasma membrane using CellMask Deep Red Plasma Membrane Stain (Cat. C10046, Thermo Fisher Scientific) according to the manufacturer's instructions and fixed the cells with 4% of paraformaldehyde. We captured the fluorescent images and conducted a line-plot analysis of the fluorescence signal using DeltaVision Elite (Cytiva).

Western blot analysis

To determine the effect of the *Nmt1* knockout on the localization of the myrVenus reporter, membrane and cytosol fractions were prepared from mESCs using Minute Plasma Membrane Protein Isolation and Cell Fractionation Kit (Cat. SM-005, Invent Biotechnologies, Inc.) following the manufacturer's protocol. Each fraction was homogenized in 1× Laemmli sample buffer (Laemmli, 1970), and protein concentrations were determined using Pierce 660nm Protein Assay Kit (Cat. 22662, Thermo Fisher Scientific) supplemented with Ionic Detergent Compatibility Reagent for Pierce 660nm Protein Assay Reagent (Cat. 22663, Thermo Fisher Scientific). On average, we obtained a 5.5-fold higher protein yield in the membrane fraction compared to the cytosol fraction. Therefore, we loaded 1.8 µg of membrane fraction proteins and 10 µg of cytosol fraction proteins on 10% Mini-PROTEAN TGX Precast Protein Gels (Cat. 4561035, Bio-Rad) to ensure that the ratio of membrane to cytosol proteins loaded on the gel reflected the protein yield from each fractionation. Separated proteins were transferred to a PVDF membrane (Cat. 170-4156, Bio-Rad). The enrichment of membrane proteins was assessed using an antibody against Na/K-ATPase (1:1000, Cat. 3010, Cell Signaling), followed by incubation with an anti-rabbit horseradish peroxidase-linked antibody (1:2000, Cat. 7074, Cell Signaling). Protein detection was performed using Chemi-Lumi One Super (Cat. 02230, Nacalai Tesque) on the FUSION-SOLO.7S.EDGE imaging system (Vilber Bio Imaging). The antibodies were stripped, and the localization of the myrVenus reporter was assessed using an anti-GFP antibody (1:2500, Cat. 598, MBL International Corp.). The remaining procedure followed the method described above. The antibodies were then stripped again, and the enrichment of cytosolic proteins was investigated using a primary antibody against GAPDH (1:3000, Cat. 2118, Cell Signaling). Band

intensities were determined using Fiji (Schindelin et al., 2012). The amounts of myrVenus in membrane and cytosol fractions were normalized by dividing the respective values by those of GAPDH and Na/K-ATPase. The ratio of normalized cytosolic myrVenus to normalized membrane myrVenus was calculated to assess the effect of the *Nmt1* knockout.

To detect FAK-Y925 phosphorylation, mEpiSCs were maintained as described in the 'Cell Line and Cell Culture' section. Subsequently, the medium was replaced with fresh DMED/F12 supplemented with non-essential amino acids, sodium pyruvate, and 0.1 mM of 2-mercaptoethanol, along with different concentrations of CGP77675, but without KSR, bFGF, or activin A for 19 h. Then, mEpiSCs were dissociated, resuspended in the same medium, and plated onto a culture dish coated with 20 µg/ml fibronectin (Cat. 354008, Corning). After 1 h, mEpiSCs were rinsed with PBS and lysed in 1× Laemmli sample buffer. Ten µg of proteins were separated and transferred to a PVDF membrane as described above. The membranes were probed with the primary antibody against Phospho-FAK (Tyr925) (1:1000, Cat. 3284, Cell Signaling). The secondary antibody and the protein detection method were the same as described above. The antibodies were stripped, and the total amount of FAK was assessed using the primary antibody against FAK (D2R2E) (1:1000, Cat. 13009, Cell Signaling). The remaining procedure for band detection followed the method described above. The inhibitory effect of CGP77675 on FAK phosphorylation was determined by dividing the band intensity of phospho-FAK by the total FAK band intensity for normalization. The results were presented by setting the value of the control (without CGP77675) to 1.

Bulk RNA-seq

For bulk RNA-seq, total RNA was extracted using the RNeasy Plus Mini Kit (Cat. 74136, Qiagen). Library preparation was performed using the TruSeq Stranded mRNA Library Prep Kit (Cat. 20020595, Illumina) according to the manufacturer’s instructions. Sequencing was performed on an Illumina NovaSeq 6000 platform in paired-end mode (2x 101 nt). Sequenced reads were mapped to the mouse (mm10) or human (hg19) reference genome sequences using TopHat2 version 2.1.1 (Trapnell et al., 2009). Raw counts were calculated using featureCounts v. 2.0.0 (Liao et al., 2014). The fragments per kilobase of exon per million mapped fragments (FPKMs) were calculated using Cuffdiff v. 2.2.1 (Trapnell et al., 2010).

In creating heatmaps for human genes specific to the epiblast, trophectoderm, and primitive endoderm, we utilized the top 25 lineage-specific genes as reported by Petropoulos et al. (Petropoulos et al., 2016). Initially, we excluded genes whose FPKM values were zero in any of the six samples (three each from control and inhibitor-treated hiPSCs) due to the low accuracy of quantitative evaluation. As a result, four genes were excluded for the primitive endoderm, while all 25 genes were retained for both the epiblast and trophectoderm. Next, for each gene across all samples, we calculated relative FPKM values against the average FPKM values of three control samples. Finally, these values were expressed on a log2 scale, and heatmaps were generated using the pheatmap package (v. 1.0.12.) in R software (v. 4.4.1).

scRNA-seq

The scRNA-Seq libraries were constructed using the Chromium Next GEM Single Cell 5’ Library and Gel Bead Kit v2 (Cat. 1000263, 10x Genomics) in accordance with the manufacturer’s instructions. Sequencing of the libraries was performed on NovaSeq 6000 (Illumina) using paired-end mode (26x90 nt), achieving over 20,000 reads per cell. The sequencing data were aligned and quantified using Cell Ranger Single-Cell Software Suite (v.7.1.0, 10x Genomics) against the Homo sapiens (human) genome assembly GRCh38 and Mus musculus (mouse) genome assembly mm10 in 2020-A 10x Genomics reference packages. The gene count data were analyzed with the R package Seurat

(v5.0, Hao et al., 2024). MEF cells were filtered with more than 1% of the counts aligned to the mm10 genome. The cell doublets and low-quality cells were filtered using a unique molecular count threshold greater than 7,500 or less than 1000. Low-quality cells with more than 7% mitochondrial counts were filtered out. Then the gene count data were normalized and scaled with the Seurat SCTransform (vars.to.regress = "percent.mt") function. The datasets of the hiPSC samples, with or without treatment with the NMT inhibitor, were integrated using the R package Harmony (v1.0, Korsunsky et al., 2019) with the RunHarmony function for batch correction. All cells were first clustered with the Seurat FindNeighbors (reduction = “harmony”, dims = 1:30) and FindClusters (resolution = 0.2) functions. Dimensionality reduction was performed by UMAP embedding using the Seurat RunUMAP (reduction = “harmony”, dims = 1:30) functions. Subclustering of cluster 6 was conducted with the Seurat FindNeighbors (reduction = “harmony”, dims = 1:30) and FindClusters (resolution = 0.5) functions. Plots of individual gene expression amounts were visualized with the Seurat FeaturePlot, FeatureScatter, and RidgePlot functions.

Blastoid formation assay

Blastoid formation from naive hiPSCs was conducted according to the published protocol (Heidari Khoei et al., 2023) with some modifications. Briefly, naive hiPSCs cultured with or without 100 μM NMT inhibitor DDD85646 were dissociated using Accutase and plated onto gelatin-coated plates in the presence of 2.5 μM Y-27632 (Cat. 036-24023, FUJIFILM Wako Chemicals) for 1 h to remove MEF feeders. Unattached cells were centrifuged, washed with Ndiff227, and plated into each well of an Elplasia 96-well plate (Cat. 4442, Corning) in 150 μl aggregation medium (Heidari Khoei *et al.*, 2023) at a density of 6,000 cells per well. Next day (day 0), we added 150 μl 2× PALLY medium (Heidari Khoei *et al.*, 2023), consisting of 1 μM PD0325901, 1 μM A83-01 (ALK4/5/7 inhibitor; Cat. 2939, Tocris), 1 μM 1-oleoyl lysophosphatidic acid (LPA, Cat. 3854, Tocris), 20 ng/mL human LIF and 20 μM Y-27632 to each well. The efficiency of blastoid formation was assessed on day 4. We also used CEPT cocktail (Cat. 033-26071, FUJIFILM Wako Chemicals) in place of Y-27632 because the CEPT cocktail was reported to increase blastoid formation efficiency compared with Y-27632 (Yu et al., 2023).

Differentiation induction of hiPSCs toward trophectoderm lineage

Trophectoderm differentiation was performed following the published protocol (Io et al., 2021a; Io et al., 2021b). Briefly, naive hiPSCs cultured with or without 100 μM NMT inhibitor DDD85646 were dissociated using Accutase and plated onto gelatin-coated plates with 2.5 μM Y-27632 to remove MEF feeders. After two hours, unattached cells were replated onto 24-well plates coated with iMatrix-511 silk (Cat. T311, Takara Bio) at a density of 2×10^4 cells/cm^2 in differentiation induction medium (day 0). The differentiation induction medium consisted of Ndiff227 supplemented with 2 μM A83-1, 2 μM PD0325901 and 2.5 μM Y-27632. The NMT inhibitor was omitted during differentiation induction. On day 1, the medium was replaced with the same medium supplemented with 1 μM JAK inhibitor (Cat. 420099, Calbiochem). On day 3, cells were dissociated, stained with an APC-conjugated anti-human TROP2 antibody (1:100, Cat. 130-115-098, Miltenyi Biotech) and a PE-conjugated anti-human CD249 (ENPEP) antibody (1:100, Cat. 564533, BD Biosciences), and analyzed using a cell sorter (SH800, SONY Corp.).

REFERENCES

Bredenkamp, N., Yang, J., Clarke, J., Stirparo, G.G., von Meyenn, F., Dietmann, S., Baker, D., Drummond, R., Ren, Y., Li, D., et al. (2019). Wnt Inhibition Facilitates RNA-Mediated Reprogramming of Human Somatic Cells to Naive Pluripotency. Stem Cell Reports *13*, 1083-1098.

10.1016/j.stemcr.2019.10.009.

Cadinanos, J., and Bradley, A. (2007). Generation of an inducible and optimized piggyBac transposon system. Nucleic Acids Res *35*, e87. 10.1093/nar/gkm446.

Frearson, J.A., Brand, S., McElroy, S.P., Cleghorn, L.A., Smid, O., Stojanovski, L., Price, H.P., Guther, M.L., Torrie, L.S., Robinson, D.A., et al. (2010). N-myristoyltransferase inhibitors as new leads to treat sleeping sickness. Nature *464*, 728-732. 10.1038/nature08893.

Guo, G., von Meyenn, F., Rostovskaya, M., Clarke, J., Dietmann, S., Baker, D., Sahakyan, A., Myers, S., Bertone, P., Reik, W., et al. (2017). Epigenetic resetting of human pluripotency. Development *144*, 2748-2763. 10.1242/dev.146811.

Guo, G., Yang, J., Nichols, J., Hall, J.S., Eyres, I., Mansfield, W., and Smith, A. (2009). Klf4 reverts developmentally programmed restriction of ground state pluripotency. Development *136*, 1063-1069. 10.1242/dev.030957.

Heidari Khoei, H., Javali, A., Kagawa, H., Sommer, T.M., Sestini, G., David, L., Slovakova, J., Novatchkova, M., Scholte Op Reimer, Y., and Rivron, N. (2023). Generating human blastoids modeling blastocyst-stage embryos and implantation. Nat Protoc *18*, 1584-1620. 10.1038/s41596-023-00802-1.

Horie, K., Kokubu, C., Yoshida, J., Akagi, K., Isotani, A., Oshitani, A., Yusa, K., Ikeda, R., Huang, Y., Bradley, A., and Takeda, J. (2011). A homozygous mutant embryonic stem cell bank applicable for phenotype-driven genetic screening. Nat Methods *8*, 1071-1077. 10.1038/nmeth.1739.

Hotta, A., Cheung, A.Y., Farra, N., Vijayaragavan, K., Séguin, C.A., Draper, J.S., Pasceri, P., Maksakova, I.A., Mager, D.L., Rossant, J., et al. (2009). Isolation of human iPS cells using EOS lentiviral vectors to select for pluripotency. Nat Methods *6*, 370-376. 10.1038/nmeth.1325.

Io, S., Iemura, Y., and Takashima, Y. (2021a). Optimized protocol for naive human pluripotent stem cell-derived trophoblast induction. STAR Protoc *2*, 100921. 10.1016/j.xpro.2021.100921.

Io, S., Kabata, M., Iemura, Y., Semi, K., Morone, N., Minagawa, A., Wang, B., Okamoto, I., Nakamura, T., Kojima, Y., et al. (2021b). Capturing human trophoblast development with naive pluripotent stem cells in vitro. Cell Stem Cell *28*, 1023-1039 e1013. 10.1016/j.stem.2021.03.013.

Jacobs, J.P., Jones, C.M., and Baille, J.P. (1970). Characteristics of a human diploid cell designated MRC-5. Nature *227*, 168-170. 10.1038/227168a0.

Kranz, A., Fu, J., Duerschke, K., Weidlich, S., Naumann, R., Stewart, A.F., and Anastassiadis, K. (2010). An improved Flp deleter mouse in C57Bl/6 based on Flpo recombinase. Genesis *48*, 512-520. 10.1002/dvg.20641.

Laemmli, U.K. (1970). Cleavage of structural proteins during the assembly of the head of bacteriophage T4. Nature *227*, 680-685. 10.1038/227680a0.

Liao, Y., Smyth, G.K., and Shi, W. (2014). featureCounts: an efficient general purpose program for assigning sequence reads to genomic features. Bioinformatics (Oxford, England) *30*, 923-930. 10.1093/bioinformatics/btt656.

Niwa, H., Yamamura, K., and Miyazaki, J. (1991). Efficient selection for high-expression transfectants with a novel eukaryotic vector. Gene *108*, 193-199. 10.1016/0378-1119(91)90434-d.

Petropoulos, S., Edsgärd, D., Reinius, B., Deng, Q., Panula, Sarita P., Codeluppi, S., Plaza Reyes, A.,

Linnarsson, S., Sandberg, R., and Lanner, F. (2016). Single-Cell RNA-Seq Reveals Lineage and X Chromosome Dynamics in Human Preimplantation Embryos. Cell *165*, 1012-1026. 10.1016/j.cell.2016.03.023.

Rhee, J.M., Pirity, M.K., Lackan, C.S., Long, J.Z., Kondoh, G., Takeda, J., and Hadjantonakis, A.K. (2006). In vivo imaging and differential localization of lipid-modified GFP-variant fusions in embryonic stem cells and mice. Genesis *44*, 202-218. 10.1002/dvg.20203.

Schindelin, J., Arganda-Carreras, I., Frise, E., Kaynig, V., Longair, M., Pietzsch, T., Preibisch, S., Rueden, C., Saalfeld, S., Schmid, B., et al. (2012). Fiji: an open-source platform for biological-image analysis. Nature Methods *9*, 676-682. 10.1038/nmeth.2019.

Suemori, H., Yasuchika, K., Hasegawa, K., Fujioka, T., Tsuneyoshi, N., and Nakatsuji, N. (2006). Efficient establishment of human embryonic stem cell lines and long-term maintenance with stable karyotype by enzymatic bulk passage. Biochem Biophys Res Commun *345*, 926-932. 10.1016/j.bbrc.2006.04.135.

Takahashi, K., Tanabe, K., Ohnuki, M., Narita, M., Ichisaka, T., Tomoda, K., and Yamanaka, S. (2007). Induction of pluripotent stem cells from adult human fibroblasts by defined factors. Cell *131*, 861-872. 10.1016/j.cell.2007.11.019.

Takashima, Y., Guo, G., Loos, R., Nichols, J., Ficz, G., Krueger, F., Oxley, D., Santos, F., Clarke, J., Mansfield, W., et al. (2014). Resetting transcription factor control circuitry toward ground-state pluripotency in human. Cell *158*, 1254-1269. 10.1016/j.cell.2014.08.029.

Trapnell, C., Pachter, L., and Salzberg, S.L. (2009). TopHat: discovering splice junctions with RNA-Seq. Bioinformatics (Oxford, England) *25*, 1105-1111. 10.1093/bioinformatics/btp120.

Trapnell, C., Williams, B.A., Pertea, G., Mortazavi, A., Kwan, G., van Baren, M.J., Salzberg, S.L., Wold, B.J., and Pachter, L. (2010). Transcript assembly and quantification by RNA-Seq reveals unannotated transcripts and isoform switching during cell differentiation. Nat Biotechnol *28*, 511-515. 10.1038/nbt.1621.

Yagita, K., Horie, K., Koinuma, S., Nakamura, W., Yamanaka, I., Urasaki, A., Shigeyoshi, Y., Kawakami, K., Shimada, S., Takeda, J., and Uchiyama, Y. (2010). Development of the circadian oscillator during differentiation of mouse embryonic stem cells in vitro. Proc Natl Acad Sci U S A *107*, 3846-3851. 10.1073/pnas.0913256107.

Yu, L., Logsdon, D., Pinzon-Arteaga, C.A., Duan, J., Ezashi, T., Wei, Y., Ribeiro Orsi, A.E., Oura, S., Liu, L., Wang, L., et al. (2023). Large-scale production of human blastoids amenable to modeling blastocyst development and maternal-fetal cross talk. Cell Stem Cell *30*, 1246-1261 e1249. 10.1016/j.stem.2023.08.002.
