# Supplementary material for: Inhibition of N-myristoyltransferase in pluripotent stem cells promotes the naive state in mice and elicits trophectoderm and primitive endoderm markers in humans
Source: Stem Cell Reports. 2025 Aug 28;20(9):102610. doi: 10.1016/j.stemcr.2025.102610 (PMC12447342; doi:10.1016/j.stemcr.2025.102610)
Supplement: Document S2. Article plus supplemental information [file mmc4.pdf]

Stem Cell Reports
Article
OPEN ACCESS

Inhibition of N-myristoyltransferase in pluripotent stem cells promotes the naive state in mice and elicits trophectoderm and primitive endoderm markers in humans

Junko Yoshida,[1,2] Hitomi Watanabe,[3] Kaori Yamauchi,[4,14] Takumi Nishikubo,[1] Ayako Isotani,[5,6] Satoshi Ohtsuka,[7,8] Hitoshi Niwa,[7,9] Yuki Kawamoto,[1] Hidenori Akutsu,[10] Akihiro Umezawa,[10] Hirofumi Suemori,[4] Yasuhiro Takashima,[11] Hideo Matsuda,[12] Gen Kondoh,[3] Junji Takeda,[2,13,*] and Kyoji Horie[1,2,15,*]

[1]Department of Physiology II, Nara Medical University, 840 Shijo-cho, Kashihara, Nara 634-8521, Japan
[2]Department of Genome Biology, Graduate School of Medicine, Osaka University, 2-2 Yamadaoka, Suita, Osaka 565-0871, Japan
[3]Laboratory of Integrative Biological Science, Institute for Life and Medical Sciences, Kyoto University, 53 Shogoin Kawahara-cho, Sakyo-ku, Kyoto 606-8507, Japan
[4]Laboratory of Embryonic Stem Cell Research, Institute for Life and Medical Sciences, Kyoto University, 53 Shogoin Kawahara-cho, Sakyo-ku, Kyoto 606-8507, Japan
[5]Genome Information Research Center, Research Institute for Microbial Diseases, Osaka University, 3-1 Yamadaoka, Suita, Osaka 565-0871, Japan
[6]Division of Biological Science, Graduate School of Science and Technology, Nara Institute of Science and Technology, 8916-5 Takayama-cho, Ikoma, Nara 630-0192, Japan
[7]Pluripotent Stem Cell Studies, RIKEN Center for Developmental Biology, 2-2-3 Minatojima-minamimachi, Chuo-ku, Kobe, Hyogo 650-0047, Japan
[8]Laboratory for Experimental Animals, Kyoto Prefectural University of Medicine, 465 Kajii-cho, Kamigyo-ku, Kyoto 602-8566, Japan
[9]Department of Pluripotent Stem Cell Biology, Institute of Molecular Embryology and Genetics, Kumamoto University, 2-2-1 Honjo, Chuo-ku, Kumamoto 860-0811, Japan
[10]Center for Regenerative Medicine, National Center for Child Health and Development, 2-10-1 Okura, Setagaya-ku, Tokyo 157-8535, Japan
[11]Department of Life Science Frontiers, Center for iPS Cell Research and Application (CiRA), Kyoto University, 53 Shogoin Kawahara-cho, Sakyo-ku, Kyoto 606-8507, Japan
[12]Department of Bioinformatic Engineering, Graduate School of Information Science and Technology, Osaka University, 1-5 Yamadaoka, Suita, Osaka 565-0871, Japan
[13]Laboratory of Immunoglycobiology, Research Institute for Microbial Diseases, Osaka University, 3-1 Yamadaoka, Suita, Osaka 565-0871, Japan
[14]Present address: RAYMEI Inc., 2-1 Yamadaoka, Suita, Osaka 565–0871, Japan
[15]Lead contact
*Correspondence: jjjtakeda@biken.osaka-u.ac.jp (J.T.), k-horie@naramed-u.ac.jp (K.H.)
https://doi.org/10.1016/j.stemcr.2025.102610

SUMMARY

Naive and primed states represent distinct phases of pluripotency during early embryonic development, both of which can be captured and interconverted *in vitro*. To understand pluripotency regulation, we performed a recessive genetic screen using homozygous mutant mouse embryonic stem cells (mESCs) and identified *N*-myristoyltransferase (NMT) as a novel regulator. Disruption of *Nmt1* in mESCs conferred resistance to differentiation, and NMT suppression in mouse epiblast stem cells (mEpiSCs) promoted the conversion from the primed to the naive state. This effect was independent of proto-oncogene tyrosine-protein kinase Src (SRC), which is a major substrate of NMT and is known to promote mESC differentiation. In contrast, NMT suppression in naive-state human induced pluripotent stem cells (hiPSCs) partially induced naive markers but, more notably, expanded subpopulations expressing trophectoderm and primitive endoderm markers, most of which co-expressed the pluripotency marker *POU5F1*. These results identify NMT as a novel regulator of pluripotency, with distinct roles in mice and humans.

INTRODUCTION

Pluripotency is the ability to differentiate into three primary germ layers and, subsequently, adult tissues. Over recent decades, various approaches have been used to capture the pluripotent state in cell cultures (Rossant and Tam, 2017), revealing distinct pluripotent states in both mice and humans. The most widely studied are the naive and primed states. The naive state corresponds to pre-implantation embryos, from which mouse embryonic stem cells (mESCs) are derived (Evans and Kaufman, 1981), while the primed state reflects post-implantation embryos, from which mouse epiblast stem cells (mEpiSCs) are established (Brons et al., 2007; Tesar et al., 2007). In contrast, human embryonic stem cells (hESCs) were in the primed state under conventional culture conditions, despite being derived from pre-implantation embryos (Thomson et al., 1998). Human induced pluripotent stem cells (hiPSCs) were also in the primed state when established under the conventional hESC culture media (Takahashi et al., 2007; Yu et al., 2007). Naive-state hESCs/hiPSCs have since been established using chemical pathway modulation or transient expression of transcription factors (Takashima et al., 2014; Theunissen et al., 2014). More recently, an intermediate "formative" pluripotent state has also been described in both species (Kalkan

© 2025 The Author(s).
Published by Elsevier Inc. on behalf of International Society for Stem Cell Research.
This is an open access article under the CC BY license (http://creativecommons.org/licenses/by/4.0/).

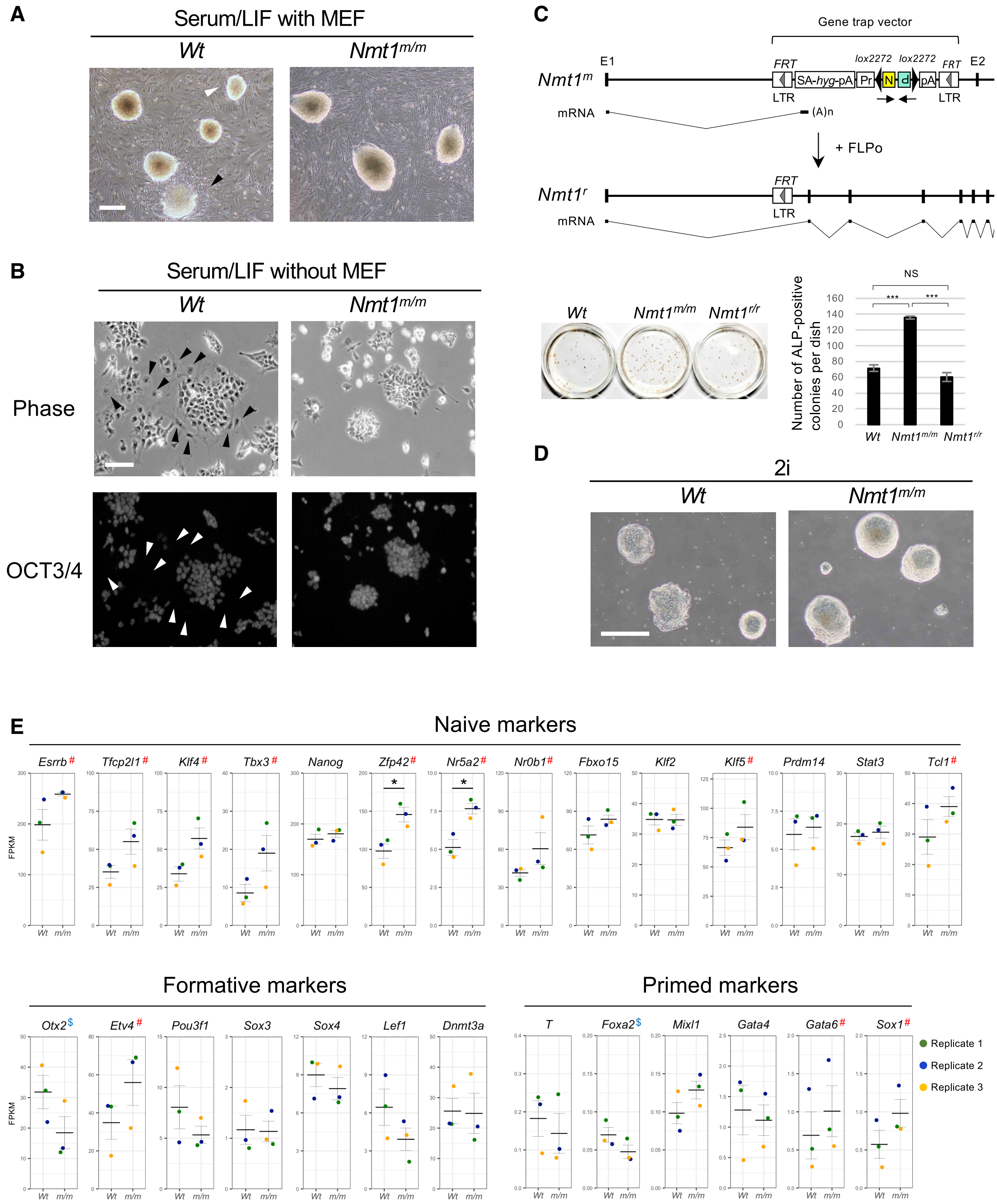

Figure 1. Disruption of *Nmt1* confers differentiation resistance to mESCs and enhances properties of the naive state

(A) Morphological differences between wild-type (WT) and *Nmt1*-homozygous mutant (*Nmt1*$^{m/m}$) mESC colonies in serum/LIF medium. mESCs were sparsely plated on MEFs to obtain single cell-derived colonies. Flat (black arrowhead) or small-sized (white arrowhead) colonies were observed in *WT* mESCs, whereas *Nmt1*$^{m/m}$ mESCs were more homogeneous in shape and size and noticeably dome shaped. Scale bar: 500 μm.

(B) OCT3/4 staining of mESCs cultured for 12 days in serum/LIF medium without MEFs. *Nmt1*$^{m/m}$ mESCs formed compact colonies with homogeneous OCT3/4 staining, whereas WT mESCs exhibited irregular-shaped colonies with scattered cells. Note that some WT mESCs are enlarged and negative for OCT3/4 (arrowheads), indicating differentiation. Scale bar: 50 μm.

(C) Reversion of differentiation resistance by deletion of the gene trap vector. (Top) Generation of the revertant allele (*Nmt1*^{r}) from the mutant allele (*Nmt1*^{m}) by Flp/*FRT* recombination. (Bottom) Differentiation resistance in each genotype. Six hundred mESCs were plated on

(legend continued on next page)

et al., 2017; Kinoshita et al., 2021; Smith, 2017). Elucidation of the regulatory mechanisms of these distinct states may advance our understanding of pluripotency and supports regenerative medicine applications.

We previously developed a method to generate homozygous mutant mESCs by converting heterozygosity to homozygosity via transient *Blm* gene inactivation (Horie et al., 2011). Expanding this approach, we established nearly 200 homozygous mutant lines, especially for genes with unknown functions. Through phenotypic screening, we found that *Nmt1*-homozygous mutant mESCs resists differentiation. *N*-myristoyltransferase (NMT) is an enzyme that attaches a myristoyl group to protein N termini (Yuan et al., 2020). Inhibition of NMT activity induced naive-state markers in mESCs and promoted the conversion of primed-state mEpiSCs to the naive state. In contrast, in hiPSCs, NMT inhibition expanded subpopulations expressing trophectoderm and primitive endoderm markers, most of which co-expressed the pluripotency marker *POU5F1*. These findings suggest that NMT is a novel regulator of pluripotency with distinct functions in mouse and human cells.

RESULTS

Disruption of *Nmt1* confers differentiation resistance to mESCs and enhances the properties of the naive state

As part of our phenotypic screening (Horie et al., 2011), we cultured single cell-derived colonies of each mESC clone on mouse embryonic fibroblasts (MEFs) in serum-containing medium. While wild-type (WT) mESCs occasionally formed flat or small-sized colonies (Figure 1A, left), *Nmt1*-homozygous mutant mESCs formed uniform, dome-shaped colonies (Figure 1A, right), suggesting resistance to differentiation. To test this, we cultured cells in serum-containing medium without MEFs. WT mESCs displayed differentiation-associated features (e.g., enlarged cells and reduced OCT3/4), whereas *Nmt1* mutants formed compact, OCT3/4-positive colonies (Figure 1B), confirming differentiation resistance. This phenotype was reversed by excising the gene trap vector via Flp/*FRT* recombination, resulting in fewer alkaline phosphatase (ALP)-positive colonies (Figure 1C). In serum-free 2i (inhibitors of MAPK/ERK kinase, MEK and glycogen synthase kinase 3, GSK3) conditions without leukemia inhibitory factor (LIF), which is known to stabilize naive state and is called ground state condition (Ying et al., 2008), both WT and *Nmt1*-mutant mESCs formed undifferentiated colonies, but the latter displayed enhanced dome-shaped morphology (Figure 1D), suggesting that NMT1 deficiency promotes the naive state.

To confirm that NMT1 deficiency promotes the naive state in mESCs, we performed RNA sequencing (RNA-seq) on three independent cultures (Figure 1E). We analyzed the expression of naive, formative, and primed markers (Carbognin et al., 2023; Kalkan et al., 2017; Kinoshita et al., 2021; Smith, 2017). *Nmt1* mutants consistently showed elevated expression of naive-state markers; mean fragments per kilobase per million mapped reads (FPKM) values increased for all except *Klf2*, and 9 markers increased in all experiments, though only two reached statistical significance. In contrast, formative and primed markers varied. These findings suggest that NMT1 deficiency specifically promotes the naive state in mESCs.

Conversion of primed-state mEpiSCs into mESC-like naive-state cells by an NMT inhibitor

Given the observed phenotypes, we hypothesized that NMT1 inhibition may facilitate the conversion of primed-state mEpiSCs into mESC-like naive-state cells. To test this possibility, we used an NMT inhibitor DDD85646 (Frearson et al., 2010), originally reported as a lead compound against NMT of *Trypanosoma brucei*. Expression of an *N*-myristoylation signal-containing Venus (myrVenus) reporter (Rhee et al., 2006) (Figure 2A) showed membrane localization that was disrupted by the inhibitor (Figure 2B), indicating effective NMT inhibition. Proto-oncogene tyrosine-protein kinase Src (SRC) undergoes *N*-myristoylation and localizes to cell membranes (Patwardhan and Resh, 2023). To validate DDD85646 functionality, we examined its effect on SRC-EGFP localization. DDD85646 reduced membrane localization of SRC-EGFP (Figure 2C), confirming its inhibition on NMT activity in mammalian cells.

Next, we tested whether the NMT inhibitor facilitates the conversion of the primed-state mEpiSCs derived from post-implantation embryos into the mESC-like naive-state cells

MEFs in serum-containing medium without LIF, and the number of undifferentiated colonies was determined by ALP staining. Data are shown as mean ± SEM ($n = 3$, independent replicates). Tukey-Kramer test; $***p < 0.001$; NS, not significant ($p > 0.05$). E, exon; LTR, long terminal repeat; SA, splice acceptor; *hyg*, hygromycin resistance gene; pA, polyadenylation signal; Pr, *Pgk1* promoter; N, neomycin-resistance gene; P, fusion gene of the puromycin-resistance gene and the herpes simplex virus thymidine kinase gene. Arrows below the gene trap vector indicate the orientation of each selection marker.

(D) Morphological differences in single cell-derived colonies between WT and $Nmt1^{m/m}$ mESCs in serum-free 2i medium without LIF and MEFs. Note that $Nmt1^{m/m}$ mESC colonies are noticeably dome shaped compared to WT mESC colonies. Scale bar: 500 µm.

(E) RNA-seq analysis presented as mean ± SEM from three independent experiments in serum/LIF medium. Unpaired Welch's t test; $*p < 0.05$. "#" and "$" indicate genes consistently up- or downregulated, respectively, across all three experiments. FPKM, fragments per kilobase per million mapped reads.

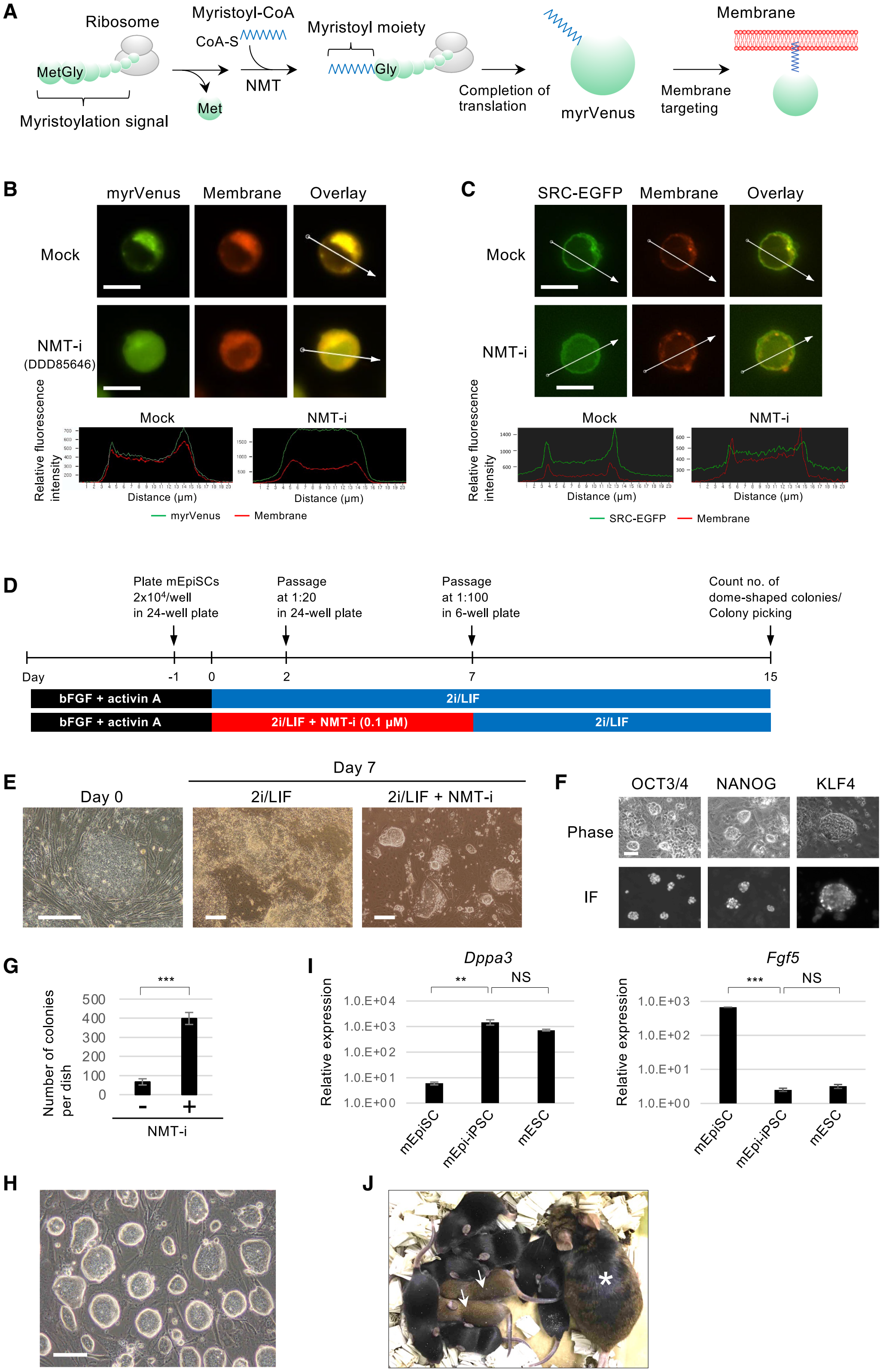

(legend on next page)

(Figure 2D). High levels of cell death and differentiation were observed in mEpiSCs under 2i/LIF, an optimal culture condition for naive-state mESCs (Figure 2E, middle), consistent with prior reports (Brons et al., 2007; Tesar et al., 2007). A combination of 2i/LIF and the NMT inhibitor DDD85646 also induced cell death and differentiation; however, dome-shaped mESC-like colonies appeared after 1 week (Figure 2E, right) and were positive for the pluripotency markers OCT3/4 and NANOG and the naive state-specific marker KLF4 (Figure 2F). Upon replating and continued culture in 2i/LIF, the increase in colony number became evident and quantifiable in the presence of the NMT inhibitor (Figure 2G). Subsequently, mESC-like clones could be established from each colony under 2i/LIF without the NMT inhibitor (Figure 2H). The gene expression pattern of these clones, named mEpi-iPSC clones, resembled naive-state mESCs, with high expression of the naive-state marker *Dppa3* and low expression of the primed-state marker *Fgf5* (Figure 2I). To further demonstrate the conversion to the naive state, we injected each mEpi-iPSC clone into pre-implantation mouse embryos and generated chimeric mice. The efficiency of generating chimeric mice is extremely low when using mEpiSCs (Brons et al., 2007; Tesar et al., 2007); however, six out of seven mEpi-iPSC clones contributed to chimeric mice, and four transmitted to germline (Figures 2J and S1A), confirming that these clones were in the naive state. It should be noted that this germline transmission cannot be considered a direct measurement of the effect of the NMT inhibitor, as the process of selecting individual colonies for clone establishment may inherently favor high-quality clones. Nevertheless, the gene expression profiles observed in the bulk cell culture (Figures 2F and 2I) support a conversion effect by NMT inhibition.

Validation of the effect of NMT1 deficiency on the primed-to-naive conversion by conditional *Nmt1* knockout

To confirm that the effect of the NMT inhibitor DDD85646 on the primed-to-naive conversion was not the off-target effect but the on-target effect, we genetically inactivated the *Nmt1* gene in the primed state and examined whether this induces a naive state, as outlined in Figure 3A. First, we converted the WT allele of the *Nmt1*-mutant heterozygous mESC line into the floxed allele (Figures 3A and S1B). The parental mESC line of this mutant harbors *ERT2-iCre-ERT2* at the *Rosa26* locus (Casanova et al., 2002; Horie et al., 2011), allowing conditional inactivation of *Nmt1* by 4-hydroxytamoxifen (4HT). Next, we differentiated the floxed mESCs into mEpiSC-like primed-state cells using basic fibroblast growth factor (bFGF) and activin A according to the published protocol (Guo et al., 2009) (Figure 3A). Last, we inactivated *Nmt1* in the primed state using 4HT and cultured under 2i/LIF to examine whether NMT1 deficiency triggers naive conversion.

Deletion of *Nmt1* was confirmed by PCR (Figure 3B) and led to reduced membrane localization of the myrVenus reporter (Figure 3C). This was further validated by fractionation and western blot analysis of membrane- and cytosol-localized myrVenus (Figure 3D). However, residual membrane localization of myrVenus remained (Figures 3C and 3D), suggesting additional mechanisms. There are two *Nmt* genes in mice, *Nmt1* and *Nmt2* (Yang et al., 2005). We speculate that the residual localization is due to NMT2 activity for the following reasons. First, our RNA-seq analysis detected NMT2 expression in both WT and *Nmt1*-homozygous mutant mESCs (Figure S1C). Second, we previously isolated an *Nmt2* mutant mESC clone by gene trap (Horie et al., 2011), registered in GenBank

Figure 2. Conversion of primed-state mEpiSCs into mESC-like naive-state cells by the NMT inhibitor

(A) Schematic of translation, myristoylation, and membrane targeting of the *N*-myristoylation signal-containing Venus reporter (myrVenus).

(B and C) The effect of the NMT inhibitor DDD85646 on subcellular localization of myrVenus (B) and SRC-EGFP (C) in mESCs. Line plots indicate the relative fluorescence intensity of fluorescent proteins and membrane staining along the white arrow shown in the overlaid picture. The plasma membrane localization of fluorescent proteins is decreased in the presence of the NMT inhibitor. Scale bar: 10 μm. NMT-i, NMT inhibitor.

(D) Schematic of the protocol for the conversion of primed-state mEpiSCs into the mESC-like naive state.

(E) Colony morphology at day 0 and day 7. Dome-shaped mESC-like colonies appeared with the NMT inhibitor. Scale bar: 200 μm.

(F) Immunostaining of cells cultured under 2i/LIF + NMT inhibitor. Dome-shaped colonies were positive for the pluripotency markers OCT3/4 and NANOG and the naive state-specific marker KLF4. IF, immunofluorescence. Scale bar: 50 μm.

(G) The number of dome-shaped colonies at day 15. Data are shown as mean ± SEM ($n = 3$, independent replicates). Unpaired Student's t test; $^{***}p < 0.001$.

(H) mESC-like cells stably maintained under 2i/LIF without the NMT inhibitor. Scale bar: 500 μm.

(I) mRNA expression of the naive-state marker *Dppa3* and the primed-state marker *Fgf5*. mEpi-iPSC indicates mESC-like cell induced from mEpiSC. Data are shown as mean ± SEM ($n = 3$, independent replicates). Dunnett's test; $^{**}p < 0.01$ and $^{***}p < 0.001$; NS, not significant ($p > 0.05$).

(J) Germline transmission of mEpi-iPSCs. An asterisk indicates a female parent chimera, and arrows denote agouti color-coated offspring.

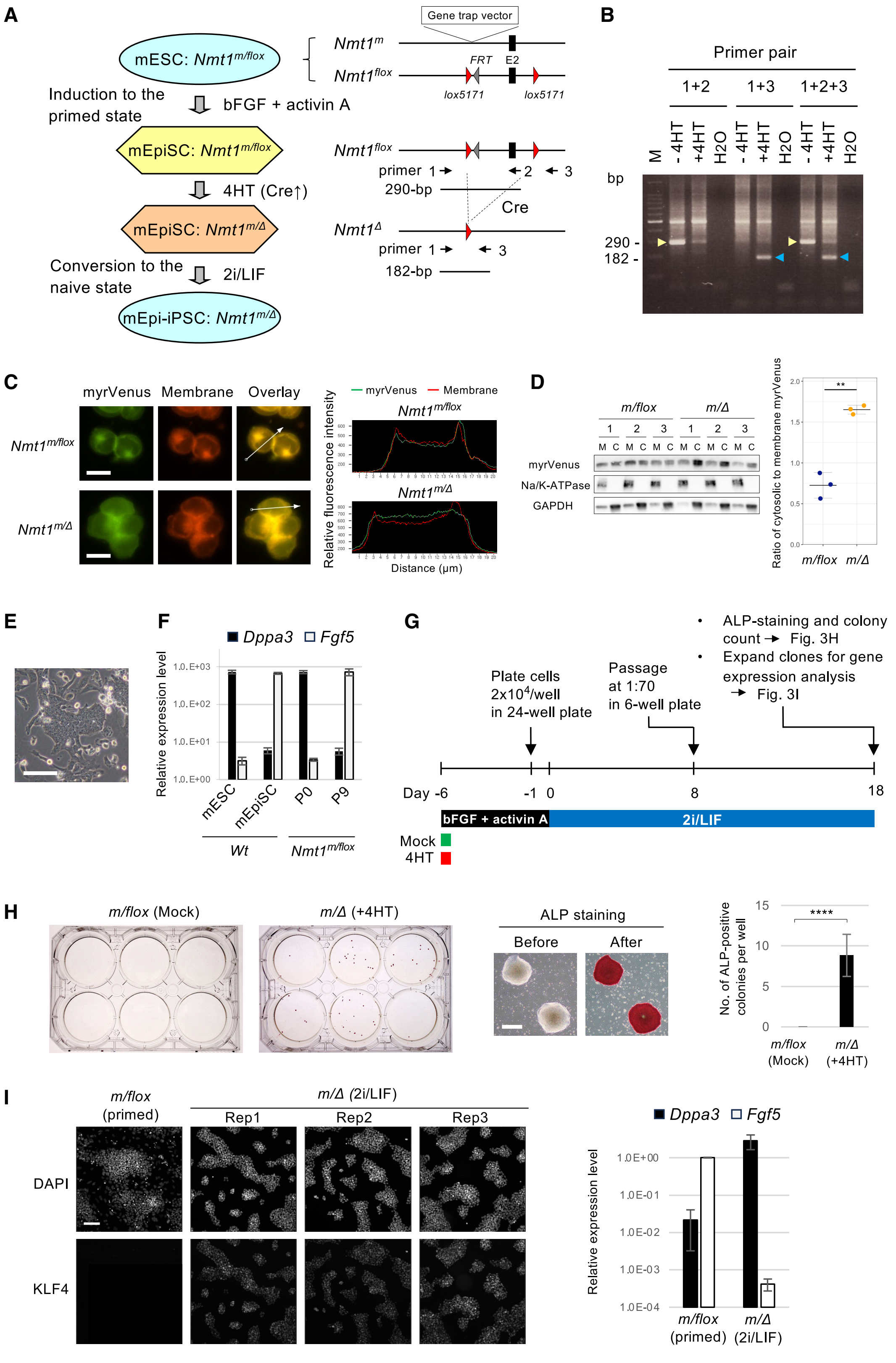

(legend on next page)

(GenBank: AB735704.1.) and in Exchangeable Gene Trap Clones (EGTC: https://egtc.jp/clones/K19F03), a member of the International Gene Trap Consortium. The gene trap vector includes a splice acceptor-hygromycin resistance cassette to identify insertions into expressed genes (Horie et al., 2011). This indicates that NMT2 is expressed at a level sufficient to confer hygromycin resistance in mESCs.

Continuous culture of mESCs under bFGF and activin A yielded mEpiSC-like flat colonies (Figure 3E). mEpiSC-like features were confirmed by a decrease in the naive-state marker *Dppa3* and the induction of the primed-state marker *Fgf5* (Figure 3F). We then inactivated the *Nmt1* gene with 4HT and cultured the cells under 2i/LIF to induce conversion to the naive state (Figure 3G). ALP-positive dome-shaped colonies appeared upon *Nmt1* inactivation but not with mock treatment (Figure 3H). These colonies were confirmed to be in the naive state by KLF4 immunostaining (Figure 3I, Left) and quantitative reverse-transcription PCR (RT-qPCR) of *Dppa3* and *Fgf5* (Figure 3I, Right). The results were consistent with the observation in the NMT inhibitor (Figure 2), demonstrating that NMT1 deficiency promotes conversion from the primed to the naive state.

The effect of the NMT inhibitor on the primed-to-naive conversion is not mediated by SRC signaling pathways

Next, we searched for NMT substrates involved in naive pluripotency regulation. Among many known targets (Yuan et al., 2020), we focused on SRC for the following reasons. First, an SRC inhibitor supports the maintenance of the naive state in mESCs and can replace the MEK inhibitor in serum-free 2i/LIF culture (Shimizu et al., 2012). Second, an SRC inhibitor was included in a chemical cocktail for establishing naive human pluripotent stem cells (hPSCs), specifically hESCs and hiPSCs (Theunissen et al., 2014). These findings led us to consider that the effects of the NMT inhibitor observed in our study may be mediated via SRC inhibition.

To test this possibility, we treated mEpiSCs with the SRC inhibitor CGP77675 (Missbach et al., 1999; Shimizu et al., 2012) and compared the effect on primed-to-naive conversion with that of the NMT inhibitor DDD85646 (Figure 4). We confirmed the dose-dependent inhibitory effect of CGP77675 on SRC kinase activity by measuring focal adhesion kinase (FAK) phosphorylation at tyrosine 925 (FAK-Y925), a major SRC target (Schlaepfer and Hunter, 1996) (Figures 4A and S1D). Based on the previous report identifying 1.5 μM as an optimal concentration for maintaining the naive state in mESCs (Shimizu et al., 2012), we tested a range of 0.5–6 μM (Figures 4B and 4C). However, no increase in conversion was observed (Figure 4D), and higher doses (≥4 μM) severely suppressed cell growth (Figure 4C). These results indicate that factors other than SRC mediate the effect of the NMT inhibitor on primed-to-naive conversion.

NMT suppression induces the expression of markers for trophectoderm and primitive endoderm in naive hiPSCs, as identified by bulk RNA-seq

To assess whether the effect of NMT suppression is conserved in humans, we first attempted primed-to-naive conversion of hESCs/hiPSCs using 2i medium with human LIF and the NMT inhibitor. However, cells differentiated gradually and undifferentiated cells were lost (Figure S2), indicating that this condition is insufficient for primed-to-naive conversion.

Next, we investigated whether the NMT inhibitor could enhance naive features in already naive hiPSCs using the

Figure 3. Verification of the effect of NMT1 deficiency by conditional gene knockout
(A) Outline of the generation of genetically modified mEpiSC-like cells and conversion to naive-state mEpi-iPSCs by conditional knockout of *Nmt1*. 4HT, 4-hydroxytamoxifen.
(B) PCR analysis of the 4HT-induced deletion of the *Nmt1* gene. Primer pairs are depicted in (A). PCR bands derived from the *Nmt1*-floxed allele and the 4HT-induced deleted allele are indicated by yellow and blue arrowheads, respectively. M, 100-bp size marker.
(C) Effect of *Nmt1* knockout on subcellular localization of the myrVenus reporter. Line plots indicate the relative fluorescence intensity of myrVenus and membrane staining along the white arrows. Plasma membrane localization is decreased upon knockout. Scale bar: 10 μm.
(D) Western blot analysis of the effect of the *Nmt1* knockout on subcellular localization of myrVenus. M, membrane fraction; C, cytosol fraction. Na/K-ATPase and GAPDH were used as reference for the enrichment of the membrane and cytosol fractions, respectively. Data are shown as mean ± SEM ($n = 3$, independent replicates). Unpaired Welch's t test; $^{**}p < 0.01$.
(E) Morphology of mEpiSC-like cells induced from mESCs at passage 9 under bFGF and activin A. Scale bar: 100 μm.
(F) Validation of primed-state identity in cells from (E) by RT-qPCR. P0, passage 0; P9, passage 9. Data are shown as mean ± SEM ($n = 3$, independent replicates).
(G) Schematic of the protocol for the conversion of primed-state mEpiSC-like cells into the naive-state mEpi-iPSCs.
(H) (Left) ALP staining of colonies post-conversion. (Middle) Representative of ALP-positive colonies. Scale bar: 500 μm. (Right) Number of ALP-positive colonies are shown as mean ± SEM ($n = 3$, independent replicates). Unpaired Student's t test; $^{****}p < 0.001$.
(I) Naive-state confirmation by immunostaining (left) and RT-qPCR (right). '*m/flox* (primed)' indicates $Nmt1^{m/flox}$ mEpiSC-like cells prior to conversion, while '*m/Δ* (2i/LIF)' refers to Cre-induced NMT1-deficient cells converted to the naive state. Scale bar: 100 μm. RT-qPCR data are shown as mean ± SEM ($n = 3$, independent replicates).

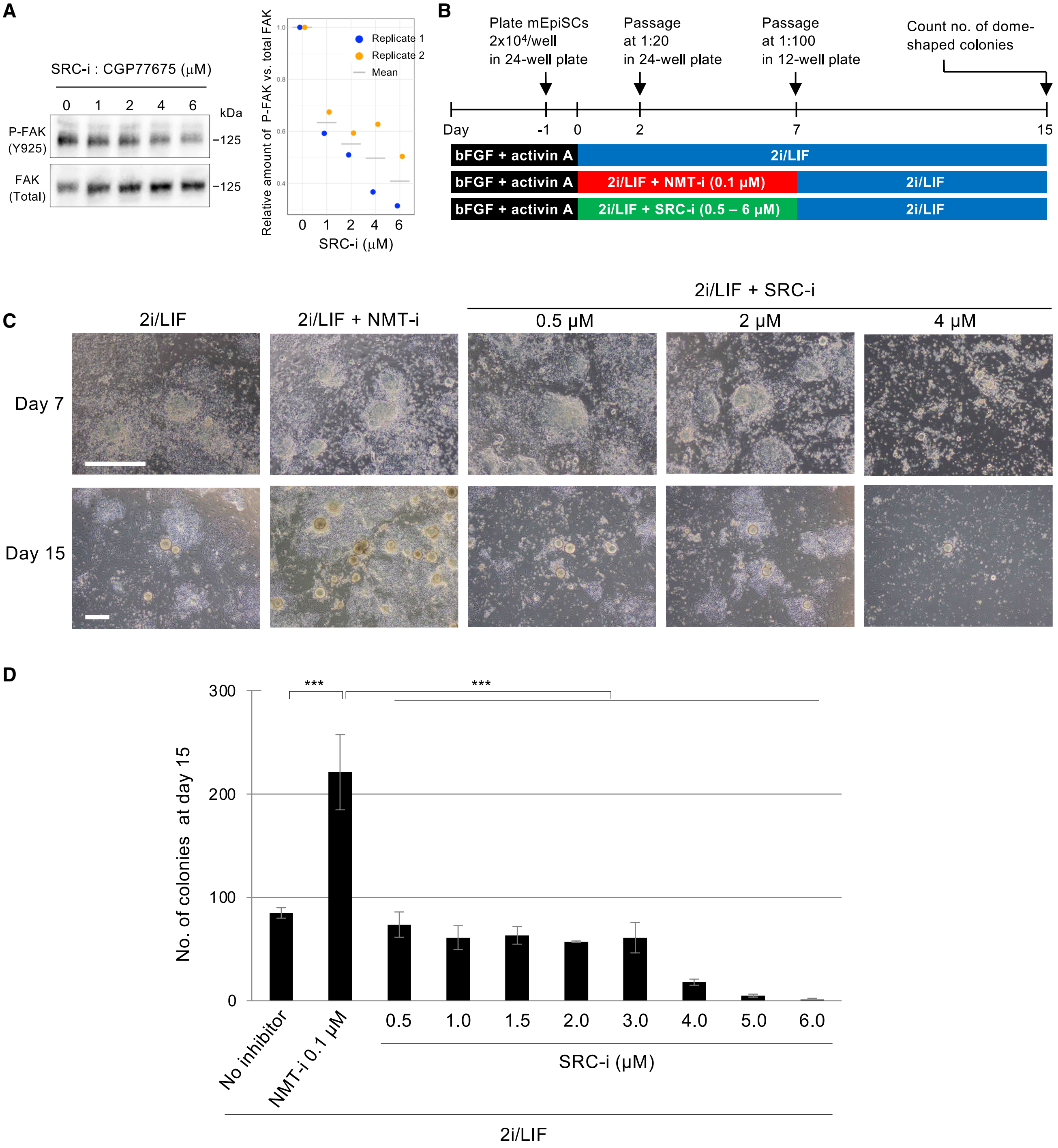

Figure 4. The effect of the NMT inhibitor on the conversion of the primed-to-naive state is not mediated by SRC signaling pathways

(A) Western blot analysis demonstrating the dose-dependent inhibitory effect of the SRC inhibitor CGP77675 on FAK phosphorylation at Y925 in mEpiSCs. The immunoblot for replicate 1 is shown on the left, and that for replicate 2 is presented in Figure S1D. SRC-i, SRC inhibitor.

(B) Schematic of the protocol for the comparison of the primed-to-naive state conversion efficiency between NMT and SRC inhibitors.

(C) A morphological view of the cells during conversion. Scale bar: 500 μm.

(D) The number of dome-shaped colonies at day 15. Data are shown as mean ± SEM (n = 3, independent replicates). Tukey-Kramer test; ***p < 0.001.

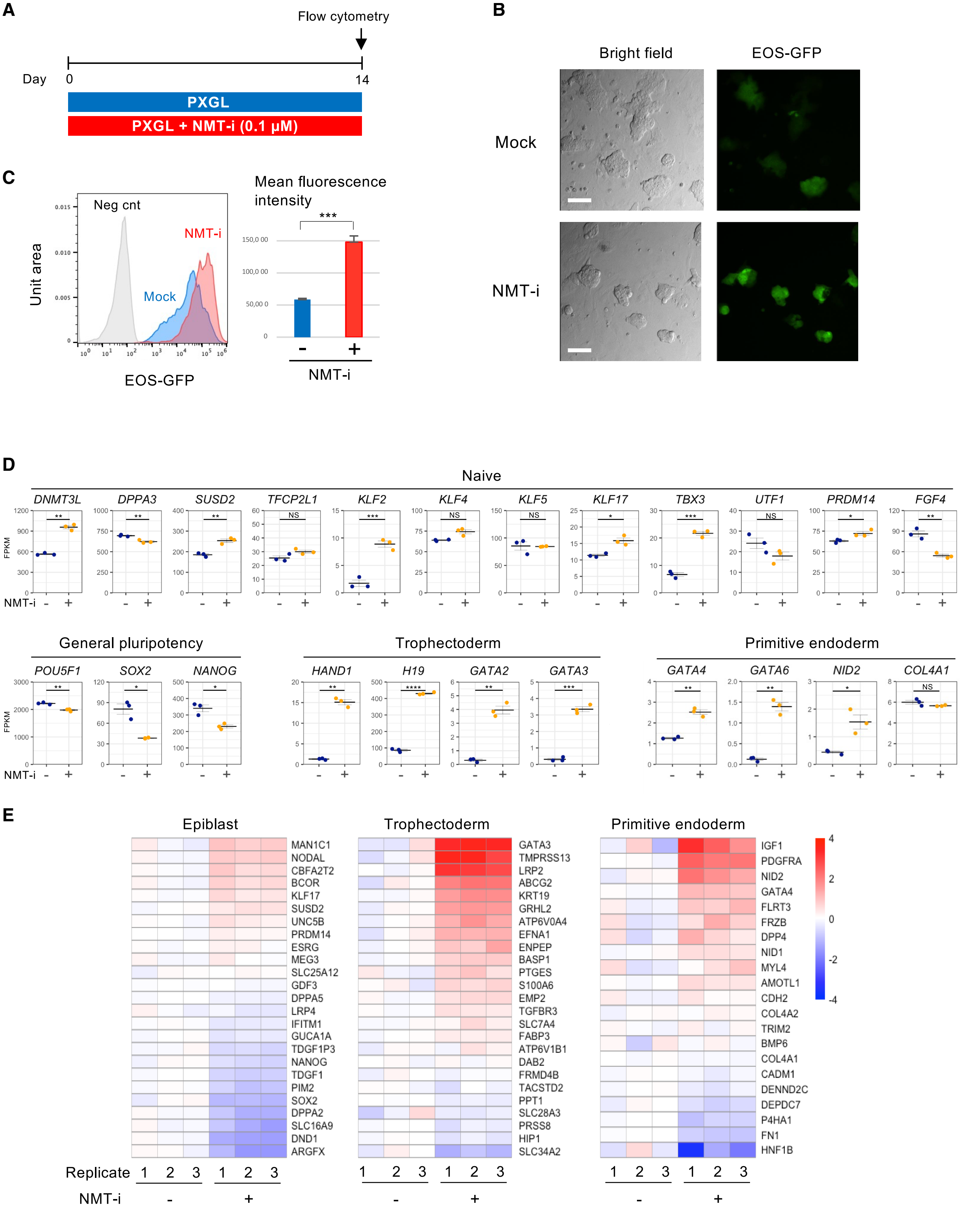

(legend on next page)

EOS-GFP reporter (Hotta et al., 2009), originally developed to identify primed-state hiPSCs and later found to be highly induced in the naive state (Takashima et al., 2014). Adipocyte-derived primed-state hiPSCs were converted to a naive state on MEF feeder cells in t2iLGö medium (Guo et al., 2017), and naive hiPSCs were separated from MEFs by the expression of SUSD2, a naive state-specific cell surface marker (Bredenkamp et al., 2019a) (Figures S3A and S3B). Within the SUSD2-positive hiPSCs, we observed EOS-GFP-negative cells (Figure S3C), suggesting incomplete conversion. Growth retardation also occurred with 0.1 μM inhibitor, the concentration used in mouse cell experiments. We therefore utilized PXGL medium (Bredenkamp et al., 2019b), a more recently developed medium optimized for naive hiPSCs. PXGL eliminated the EOS-GFP-negative population and alleviated growth retardation caused by the NMT inhibitor (Figures 5A–5C). We then cultured hiPSCs with or without 0.1 μM NMT inhibitor for 2 weeks, resulting in a 2.5-fold increase in EOS-GFP signal with NMT inhibition (Figure 5C).

To further investigate the effect of the NMT suppression, we performed RNA-seq. In contrast to mESCs, where most naive markers were upregulated (Figure 1E), only partial induction of naive markers was observed in hiPSCs (Figure 5D). However, *KLF2* and *TBX3* showed substantial increases, with 5.6-fold and 3.3-fold upregulation, respectively (Figure 5D). While *KLF2* is expressed at low levels in naive hPSCs (Guo et al., 2016), its forced expression with *NANOG* has been reported to facilitate primed-to-naive conversion (Takashima et al., 2014; Theunissen et al., 2014), suggesting that its upregulation may mediate the effects of the NMT inhibitor. General pluripotency markers, such as *POU5F1* (encoding OCT3/4), *SOX2*, and *NANOG*, remained highly expressed, though *SOX2* and *NANOG* showed modest reductions (Figure 5D).

Unexpectedly, trophectoderm and primitive endoderm markers significantly increased with NMT inhibitor treatment (Figure 5D). These increases were absent in NMT1-deficient mESCs (Figure S1E), suggesting a response specific to hPSCs. To explore this further, we examined top-ranked markers of epiblast, trophectoderm, and primitive endoderm using single-cell RNA sequencing (scRNA-seq) data from human pre-implantation embryos (Petropoulos et al., 2016). This confirmed significant induction of trophectoderm and primitive endoderm markers, with stronger upregulation seen in the trophectoderm lineage (Figure 5E).

NMT suppression expands hiPSC subpopulations expressing trophectoderm and primitive endoderm markers while co-expressing pluripotency markers, as identified by scRNA-seq

Bulk RNA-seq analysis (Figure 5) could not determine whether the increased expression of trophectoderm and primitive endoderm markers was due to expansion of specific cell populations or a global change across all cells. Recent scRNA-seq studies have shown that naive hiPSCs cultured in 5iLAF medium (Theunissen et al., 2014), a well-established naive-state medium, exhibit cellular heterogeneity, with subsets expressing trophectoderm, primitive endoderm, and eight-cell (8C)-stage markers (Moya-Jódar et al., 2023). The marker upregulation observed in our bulk RNA-seq data (Figure 5) suggests that NMT inhibition may enhance this heterogeneity. To investigate this, we performed scRNA-seq on hiPSCs cultured with or without 0.1 μM NMT inhibitor for 2 weeks.

Naive hiPSCs were grouped into clusters 0–7 (Figure 6A), with clusters 6 and 7 expanding in response to NMT inhibition (Figure 6B). Independent of NMT inhibition, general pluripotency markers such as *POU5F1*, *SOX2*, and *NANOG* were broadly expressed, though their levels were relatively low in cluster 7 (Figures 6C and S4A). Similarly, naive-state markers such as *DNMT3L*, *DPPA3*, and *PRDM14* were expressed at lower levels in this cluster (Figures 6D and S4A). In contrast, trophectoderm markers including *HAND1*, *H19*, *GATA2*, and *GATA3* were primarily expressed in cluster 7, with additional expression also noted in cluster 6 (Figures 6E and S4A). Therefore, as previously reported in 5iLAF medium, naive hiPSCs cultured in PXGL medium also contained subpopulations expressing trophectoderm markers. Expression of these markers was further enhanced by NMT inhibition (Figures 6E and S4A), supporting the notion that the increase in trophectoderm markers observed in bulk RNA-seq reflects subpopulation expansion, rather than a global effect. Subpopulations expressing primitive endoderm markers have also been reported in

Figure 5. NMT suppression induces the expression of markers for trophectoderm and primitive endoderm in naive hiPSCs, as identified by bulk RNA-seq

(A) Schematic of the protocol for assessing the effect of the NMT inhibitor on naive hiPSCs in PXGL medium.

(B) Morphology of naive hiPSCs and EOS-GFP reporter expression. Scale bar: 100 μm.

(C) Flow cytometric analysis of EOS-GFP expression. Negative control is primed-state hiPSCs. The mean fluorescence intensity of EOS-GFP is shown as mean ± SEM ($n = 3$, independent replicates). Unpaired Student's t test; ***$p < 0.001$.

(D) Bulk RNA-seq analysis presented as mean ± SEM from three independent cultures. Unpaired Welch's t test; *$p < 0.05$, **$p < 0.01$, and ***$p < 0.001$; NS, not significant ($p > 0.05$).

(E) Relative expression levels of the epiblast-, trophectoderm-, and primitive endoderm-specific genes (Petropoulos et al., 2016).

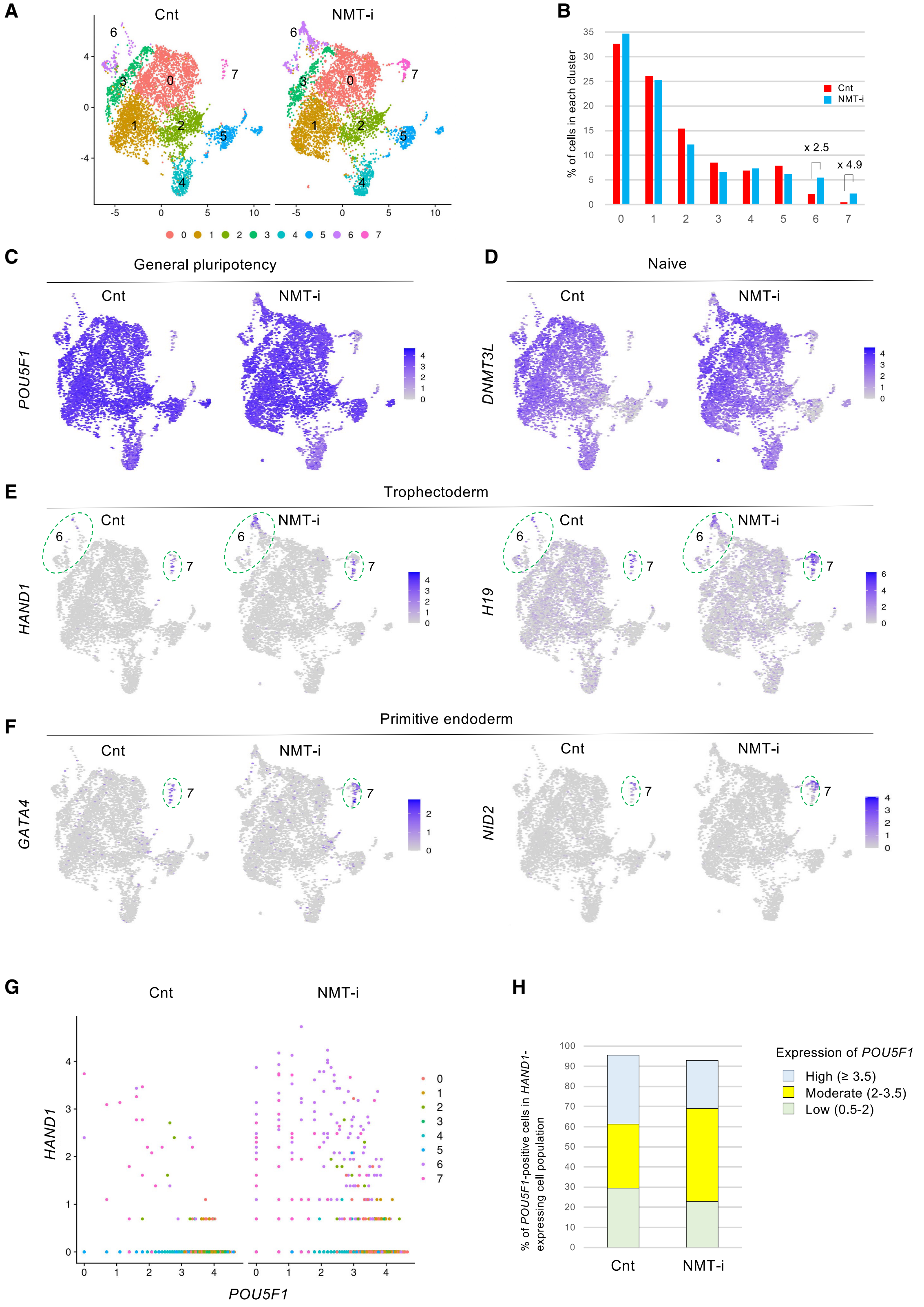

(legend on next page)

5iLAF medium (Moya-Jódar et al., 2023). Likewise, primitive endoderm markers, such as *GATA4*, *GATA6*, *NID2*, and *COL4A1*, were expressed in cluster 7, and their expression was enhanced by NMT inhibition (Figures 6F and S4A).

Notably, most cells expressing trophectoderm or primitive endoderm markers also co-expressed the pluripotency marker *POU5F1*, with or without the NMT inhibitor (Figures 6G, 6H, S4B, and S4C). This co-expression resembles transitional states in human embryos (Petropoulos et al., 2016), suggesting that NMT inhibition may enhance developmental plasticity within the naive population. However, when we tested functional outcomes by performing blastoid formation (Kagawa et al., 2022; Yanagida et al., 2021; Yu et al., 2021) and trophectoderm differentiation assays (Guo et al., 2021; Io et al., 2021), no enhancement was observed under NMT inhibition (Figure S5).

A small fraction of naive hiPSCs express markers of 8C-stage human embryos (Mazid et al., 2022; Moya-Jódar et al., 2023; Taubenschmid-Stowers et al., 2022). These cells are referred to as 8C-like cells (8CLCs) and express totipotency-associated genes such as *ZSCAN4*, *DUXA*, and *TPRX1*. *ZSCAN4*-positive cells were found in cluster 6 (Figure S6A) but were distinct from trophectoderm-expressing population (Figure 6E). Subclustering of cluster 6 revealed that the trophectoderm-positive subset (6s1) expanded markedly (19.5-fold) by NMT inhibition, whereas 8CLC-associated subsets (6s2 and 6s3) did not increase (Figures S6B–S6D). Thus, NMT suppression does not induce 8CLCs.

These findings demonstrate that NMT inhibition induces the expression of trophectoderm and primitive endoderm markers in naive hiPSCs while maintaining co-expression of pluripotency markers. In contrast, NMT suppression in mouse cells primarily induces naive-state markers, suggesting intrinsic species-specific differences in the naive state.

DISCUSSION

In this study, we identified NMT as a novel regulator of the naive state, with distinct functions in mice and humans. NMT catalyzes the attachment of myristate, a 14-carbon fatty acid, to the N-terminal glycine residue of proteins (Yuan et al., 2020). The significance of myristoylation during early development is underscored by the embryonic lethality observed in *Nmt1*-knockout mice (Yang et al., 2005). Myristoylation increases protein hydrophobicity, promoting membrane targeting and clustering of signaling molecules, thereby activating diverse pathways (Yuan et al., 2020). Considering this observation, an attractive model to explain how NMT suppression enhances the naive state in mice is that it shields cells from external differentiation stimuli by reducing the density of signaling molecules at the plasma membrane. This concept resembles the mechanism by which 2i inhibitors stabilize the naive state (Ying et al., 2008). MEK, a key 2i target, acts as a signal transducer in the FGF2-dependent differentiation pathway. Thus, naive cells cultured in 2i are sequestered from a major differentiation stimulus (Ying et al., 2008). Our results indicated that 2i alone was inefficient in converting mouse cells from the primed to the naive state, as reported previously (Guo et al., 2009). In contrast, the addition of the NMT inhibitor enhanced the efficiency of this conversion (Figure 2G). Furthermore, the dome-shaped morphology of the colonies, a characteristic feature of naive cells, became more pronounced with the addition of the NMT inhibitor to 2i medium, indicating a non-overlapping effect between MEK and NMT inhibitors (Figure 1D). These observations suggest that signaling pathways other than the FGF2-MEK axis are targeted by the NMT inhibitor.

SRC, a known NMT substrate, promotes mESC differentiation (Meyn et al., 2005), and its inhibition stabilizes the naive state (Shimizu et al., 2012). Therefore, we initially hypothesized that SRC mediates the conversion of primed-state mEpiSCs into naive-state cells by NMT suppression. However, the SRC inhibitor did not enhance this conversion (Figure 4), suggesting that other Nmt substrates are involved. Advances in proteomics have identified more than 100 *N*-myristoylated proteins in HeLa cells (Thinon et al., 2014). Comparative profiling of naive and primed states may uncover novel pluripotency regulators.

Various roles other than plasma membrane targeting have been reported for myristoylation. A hydrophobic myristoyl moiety can alter protein folding and provide a novel interface for protein-protein interactions (Spassov et al., 2018). The myristoylation of proteasome components controls the shuttling of proteasome complexes between nucleus

Figure 6. NMT suppression expands hiPSC subpopulations expressing trophectoderm and primitive endoderm markers while co-expressing pluripotency markers, as identified by scRNA-seq

(A) Uniform Manifold Approximation and Projection (UMAP) plots of naive hiPSCs in the absence (Cnt, control: 5,360 cells) and presence (NMT-i: 5,506 cells) of the NMT inhibitor.

(B) Cluster proportions from (A).

(C–F) UMAPs showing expression of developmental markers. Clusters 6 and 7 are circled.

(G) Scatterplots of *POU5F1* and *HAND1* co-expression, colored by clusters 0–7 as in (A).

(H) Quantification of co-expression in (G), stratified by *POU5F1* levels.

and cytoplasm, which regulates the degradation of misfolded proteins (Kimura et al., 2016). Several proteins involved in apoptosis are myristoylated following caspase-mediated cleavage, which can enhance or reduce the activity of each protein and influence the balance between cell death and survival (Martin et al., 2008). These or other unknown mechanisms may be involved in the regulation of pluripotency observed in our study.

Transcriptome analyses of naive hiPSCs treated with the NMT inhibitor revealed an upregulation of trophectoderm and primitive endoderm markers (Figures 5D, 5E, and 6), in contrast to the findings in mouse cells (Figure 1). Given that naive hPSCs naturally express these markers, and that we observed co-expression of the pluripotency marker *POU5F1* with these lineage markers in the presence of the NMT inhibitor (Figures 6G, 6H, S4B, and S4C), we hypothesized that this upregulation might reflect increased differentiation plasticity rather than a loss of pluripotency. Nonetheless, blastoid formation and trophectoderm differentiation assays showed no enhancement (Figure S5). Thus, the functional significance of this marker upregulation remains unclear and warrants further investigation.

We consider that there is room to improve the efficacy and specificity of the NMT inhibitor because our conditional *Nmt1*-knockout experiment gave superior results compared to the NMT inhibitor in terms of the induction level of naive cells from primed-state cells (Figures 2G and 3H). Recently, NMT has attracted increasing attention as a therapeutic target in cancers, and new NMT inhibitors are being developed accordingly (Yuan et al., 2020). The NMT inhibitor used in the present study (DDD85646) was originally developed as a lead compound to target NMT of *Trypanosoma brucei* (Frearson et al., 2010) and is therefore unlikely to be an optimal inhibitor for mammalian NMT. The newly developed inhibitors optimized for human NMT may regulate pluripotent stem cells more effectively.

METHODS

Full experimental details are available in the supplemental information.

Cell line and cell culture

Experiments using hiPSCs, including blastoid formation assay, were approved by the institutional review board of Nara Medical University. The *Nmt1*-mutant mESC clone was obtained previously (Horie et al., 2011). The retroviral gene trap vector was inserted at the first intron of the *Nmt1* gene. The flanking sequence of the insertion site is 5′-ATCCCACGCTGGTCTCATTTGGACA-3′. mESCs, mEpiSCs, hiPSCs, and hESCs were cultured under standard conditions.

Immunostaining

For immunostaining of OCT3/4, NANOG, and KLF4, cells were fixed in 4% paraformaldehyde in PBS for 10 min, permeabilized with 0.2% Triton X-100 for 10 min, and stained using a standard protocol with the following primary antibodies: anti-OCT3/4 mouse monoclonal antibody (1:300, clone c-10, Cat. Sc-5279, Santa Cruz Biotechnology), anti-NANOG rabbit polyclonal antibody (1:200, Cat. RCAB002P-F, ReproCELL), and anti-KLF4 rabbit monoclonal antibody (1:1,000, Cat. ab214666, Abcam). For SUSD2 staining, cells were incubated with allophycocyanin(APC)-conjugated anti-SUSD2 antibody (1:20, clone W5C5, Cat. 327401, BioLegend) in culture medium for 30 min and analyzed by flow cytometry.

Generating chimeric mice and assessing germline transmission

All animal experiments were approved by the institutional review boards of Nara Medical University, Osaka University, and Kyoto University, and were performed in accordance with institutional guidelines. mEpi-iPSCs were injected into 8C-stage embryos or blastocysts derived from ICR or BDF1 mice. As the parental mEpiSCs originated from a female 129SV mouse, female chimeric mice with agouti coat color were selected and crossed with male C57BL/6J mice. Germline transmission was assessed based on the presence of agouti-colored progeny.

Generating the conditional allele at the *Nmt1* locus

We conducted a standard gene targeting to flox the second exon of the WT allele of the *Nmt1*-heterozygous mESC clone. The targeting vector was constructed by a standard protocol using PCR primers listed in Table S1.

Conversion between primed and naive states

mESCs carrying the floxed *Nmt1* allele were converted into mEpiSC-like primed-state cells using a published protocol (Guo et al., 2009). To induce the naive state, the *Nmt1* allele was deleted via 4HT-induced Cre activation, followed by culture in 2i/LIF medium.

RT-qPCR

Expression levels of *Dppa3*, *Fgf5*, and *Actb* in mouse cells were quantified by real-time PCR using the LightCycler FastStart DNA Master SYBR Green I kit (Cat. 12239264001, Roche Diagnostics) using primers listed in Table S1. Gene expression in hESCs (KhES-1) was analyzed using the hESC RT^2 Profiler PCR array (Cat. PAHS-081, QIAGEN).

Microscopic analysis of myrVenus reporter localization

The expression vector of the myrVenus reporter (Rhee et al., 2006) was constructed as described in the

supplemental information. The plasma membrane was stained with CellMask Deep Red plasma membrane stain (Cat. C10046, Thermo Fisher Scientific), and fluorescence signals were evaluated by line-plot analysis using the DeltaVision Elite system (Cytiva).

Western blot analysis

To examine the effect of the *Nmt1* knockout on the localization of the myrVenus reporter, membrane and cytosol fractions were prepared from mESCs using Minute plasma membrane protein isolation and cell fractionation kit (Cat. SM-005, Invent Biotechnologies, Inc.) and analyzed by standard western blotting. Primary antibodies used were anti-GFP (1:2,500, Cat. 598, MBL International Corp.), anti-Na/K-ATPase (1:1,000, Cat. 3010, Cell Signaling Technology), and anti-GAPDH (1:3,000, Cat. 2118, Cell Signaling Technology). The amounts of myrVenus in each fraction were normalized to GAPDH for the cytosol fraction and Na/K-ATPase for the membrane fraction.

To assess the inhibitory effect of CGP77675 on FAK phosphorylation, western blotting was performed using anti-Phospho-FAK (Tyr925) (1:1,000, Cat. 3284, Cell Signaling Technology) and anti-total FAK (D2R2E) (1:1,000, Cat. 13009, Cell Signaling Technology). Phospho-FAK levels were normalized by total FAK.

Bulk RNA-seq and scRNA-seq

Bulk RNA-seq libraries were prepared from total RNA using the TruSeq stranded mRNA library prep kit (Cat. 20020595, Illumina). scRNA-Seq libraries were generated using the Chromium Next GEM Single Cell 5′ Library and Gel Bead Kit v.2 (Cat. 1000263, 10× Genomics). Sequencing was performed on an Illumina NovaSeq 6000 platform in paired-end mode (2 × 101 nt). Details of the bioinformatic procedures are provided in the supplemental information. Gene expression levels in mESCs and hiPSCs are presented as FPKM values in Tables S2 and S3, respectively.

Blastoid formation and trophectoderm differentiation assays

Blastoid formation from naive hiPSCs was performed according to the published protocol on an Elplasia 96-well plate (Cat. 4442, Corning), with modifications detailed in the supplemental information. Trophectoderm differentiation was carried out following the published protocol with or without BMP4.

Statistical analysis

Student's t test or Welch's t test was used for comparisons between two groups. Tukey-Kramer test was applied for multiple comparisons across all groups, and Dunnett's test for comparisons to a control.

RESOURCE AVAILABILITY

Lead contact

Requests for further information and resources should be directed to and will be fulfilled by the lead contact, Kyoji Horie (k-horie@naramed-u.ac.jp).

Materials availability

The *Nmt1*-homozygous mutant mESC line Nmt1-K1 and its parental mESC line vdR2-4 have been deposited in the Japanese Collection of Reseach Bioresources (JCRB) Cell Bank (https://cellbank.nibn.go.jp/english) under the accession numbers AyuK8A06 and JCRB1658, respectively.

Data and code availability

RNA-seq data have been deposited in the DNA Data Bank of Japan (DDBJ) BioProject database under accession number PRJDB18034. This paper does not report any original code.

ACKNOWLEDGMENTS

We acknowledge the NGS core facility of the Genome Information Research Center at the Research Institute for Microbial Diseases of Osaka University for supporting RNA-seq experiments. We thank Dr. Paul Wyatt at the Drug Discovery Unit, School of Life Sciences, University of Dundee, for providing DDD85646 (prepared with the support of Wellcome Trust grant WT 077705). We also thank Dr. Paul Tesar for the mEpiSCs; Dr. Kat Hadjantonakis for the myrVenus reporter vector; Dr. Masaru Okabe for supporting the generation of chimeric mice; and Drs. Daisuke Okuzaki, Miwa Sasai, and Masahiro Yamamoto for their assistance with the RNA-seq experiments. This work was supported by Grants-in-Aid for Scientific Research from the Ministry of Education, Culture, Sports, Science and Technology of Japan (JP16H04683, JP18K19275, and JP20H03174 for K.H.); JST PRESTO (K.H.); AMED (JP20bm0704035 for Y.T.); and the Cooperative Research Program (Joint Usage/Research Center program) of Institute for Life and Medical Sciences, Kyoto University (K.H.). This work was also supported in part by the research grant from the Takeda Science Foundation (K.H.), Naito Foundation (K.H.), and Daiichi Sankyo Foundation of Life Science (K.H.).

AUTHOR CONTRIBUTIONS

Conceptualization, K.H. and J.T.; methodology, J.Y., H.M., and K.H.; investigation, J.Y., H.W., K.Y., T.N., A.I., Y.K., H.A., H.S., Y.T., H.M., G.K., and K.H.; writing – original draft, K.H.; resources, S.O., H.N., and Y.T.; supervision, H.A., A.U., H.S., H.M., J.T., and K.H.; project administration, J.T. and K.H.; funding acquisition, Y.T., H.M., and K.H.

DECLARATION OF INTERESTS

The authors declare no competing interests.

DECLARATION OF GENERATIVE AI AND AI-ASSISTED TECHNOLOGIES IN THE WRITING PROCESS

During the preparation of this work, the authors used ChatGPT in order to improve language and readability. After using this

tool/service, the authors reviewed and edited the content as needed and take full responsibility for the content of the publication.

SUPPLEMENTAL INFORMATION

Supplemental information can be found online at https://doi.org/10.1016/j.stemcr.2025.102610.

Received: July 17, 2021
Revised: July 29, 2025
Accepted: July 30, 2025
Published: August 28, 2025

REFERENCES

Bredenkamp, N., Stirparo, G.G., Nichols, J., Smith, A., and Guo, G. (2019a). The Cell-Surface Marker Sushi Containing Domain 2 Facilitates Establishment of Human Naive Pluripotent Stem Cells. Stem Cell Rep. *12*, 1212–1222. https://doi.org/10.1016/j.stemcr.2019.03.014.

Bredenkamp, N., Yang, J., Clarke, J., Stirparo, G.G., von Meyenn, F., Dietmann, S., Baker, D., Drummond, R., Ren, Y., Li, D., et al. (2019b). Wnt Inhibition Facilitates RNA-Mediated Reprogramming of Human Somatic Cells to Naive Pluripotency. Stem Cell Rep. *13*, 1083–1098. https://doi.org/10.1016/j.stemcr.2019.10.009.

Brons, I.G.M., Smithers, L.E., Trotter, M.W.B., Rugg-Gunn, P., Sun, B., Chuva de Sousa Lopes, S.M., Howlett, S.K., Clarkson, A., Ahrlund-Richter, L., Pedersen, R.A., and Vallier, L. (2007). Derivation of pluripotent epiblast stem cells from mammalian embryos. Nature *448*, 191–195. https://doi.org/10.1038/nature05950.

Carbognin, E., Carlini, V., Panariello, F., Chieregato, M., Guerzoni, E., Benvegnù, D., Perrera, V., Malucelli, C., Cesana, M., Grimaldi, A., et al. (2023). Esrrb guides naive pluripotent cells through the formative transcriptional programme. Nat. Cell Biol. *25*, 643–657. https://doi.org/10.1038/s41556-023-01131-x.

Casanova, E., Fehsenfeld, S., Lemberger, T., Shimshek, D.R., Sprengel, R., and Mantamadiotis, T. (2002). ER-based double iCre fusion protein allows partial recombination in forebrain. Genesis *34*, 208–214. https://doi.org/10.1002/gene.10153.

Evans, M.J., and Kaufman, M.H. (1981). Establishment in culture of pluripotential cells from mouse embryos. Nature *292*, 154–156. https://doi.org/10.1038/292154a0.

Frearson, J.A., Brand, S., McElroy, S.P., Cleghorn, L.A.T., Smid, O., Stojanovski, L., Price, H.P., Guther, M.L.S., Torrie, L.S., Robinson, D.A., et al. (2010). N-myristoyltransferase inhibitors as new leads to treat sleeping sickness. Nature *464*, 728–732. https://doi.org/10.1038/nature08893.

Guo, G., Stirparo, G.G., Strawbridge, S.E., Spindlow, D., Yang, J., Clarke, J., Dattani, A., Yanagida, A., Li, M.A., Myers, S., et al. (2021). Human naive epiblast cells possess unrestricted lineage potential. Cell Stem Cell *28*, 1040–1056.e6. https://doi.org/10.1016/j.stem.2021.02.025.

Guo, G., von Meyenn, F., Rostovskaya, M., Clarke, J., Dietmann, S., Baker, D., Sahakyan, A., Myers, S., Bertone, P., Reik, W., et al. (2017). Epigenetic resetting of human pluripotency. Development *144*, 2748–2763. https://doi.org/10.1242/dev.146811.

Guo, G., von Meyenn, F., Santos, F., Chen, Y., Reik, W., Bertone, P., Smith, A., and Nichols, J. (2016). Naive Pluripotent Stem Cells Derived Directly from Isolated Cells of the Human Inner Cell Mass. Stem Cell Rep. *6*, 437–446. https://doi.org/10.1016/j.stemcr.2016.02.005.

Guo, G., Yang, J., Nichols, J., Hall, J.S., Eyres, I., Mansfield, W., and Smith, A. (2009). Klf4 reverts developmentally programmed restriction of ground state pluripotency. Development *136*, 1063–1069. https://doi.org/10.1242/dev.030957.

Horie, K., Kokubu, C., Yoshida, J., Akagi, K., Isotani, A., Oshitani, A., Yusa, K., Ikeda, R., Huang, Y., Bradley, A., and Takeda, J. (2011). A homozygous mutant embryonic stem cell bank applicable for phenotype-driven genetic screening. Nat. Methods *8*, 1071–1077. https://doi.org/10.1038/nmeth.1739.

Hotta, A., Cheung, A.Y.L., Farra, N., Vijayaragavan, K., Séguin, C.A., Draper, J.S., Pasceri, P., Maksakova, I.A., Mager, D.L., Rossant, J., et al. (2009). Isolation of human iPS cells using EOS lentiviral vectors to select for pluripotency. Nat. Methods *6*, 370–376. https://doi.org/10.1038/nmeth.1325.

Io, S., Kabata, M., Iemura, Y., Semi, K., Morone, N., Minagawa, A., Wang, B., Okamoto, I., Nakamura, T., Kojima, Y., et al. (2021). Capturing human trophoblast development with naive pluripotent stem cells in vitro. Cell Stem Cell *28*, 1023–1039.e13. https://doi.org/10.1016/j.stem.2021.03.013.

Kagawa, H., Javali, A., Khoei, H.H., Sommer, T.M., Sestini, G., Novatchkova, M., Scholte Op Reimer, Y., Castel, G., Bruneau, A., Maenhoudt, N., et al. (2022). Human blastoids model blastocyst development and implantation. Nature *601*, 600–605. https://doi.org/10.1038/s41586-021-04267-8.

Kalkan, T., Olova, N., Roode, M., Mulas, C., Lee, H.J., Nett, I., Marks, H., Walker, R., Stunnenberg, H.G., Lilley, K.S., et al. (2017). Tracking the embryonic stem cell transition from ground state pluripotency. Development *144*, 1221–1234. https://doi.org/10.1242/dev.142711.

Kimura, A., Kurata, Y., Nakabayashi, J., Kagawa, H., and Hirano, H. (2016). N-Myristoylation of the Rpt2 subunit of the yeast 26S proteasome is implicated in the subcellular compartment-specific protein quality control system. J. Proteomics *130*, 33–41. https://doi.org/10.1016/j.jprot.2015.08.021.

Kinoshita, M., Barber, M., Mansfield, W., Cui, Y., Spindlow, D., Stirparo, G.G., Dietmann, S., Nichols, J., and Smith, A. (2021). Capture of Mouse and Human Stem Cells with Features of Formative Pluripotency. Cell Stem Cell *28*, 453–471.e8. https://doi.org/10.1016/j.stem.2020.11.005.

Martin, D.D.O., Vilas, G.L., Prescher, J.A., Rajaiah, G., Falck, J.R., Bertozzi, C.R., and Berthiaume, L.G. (2008). Rapid detection, discovery, and identification of post-translationally myristoylated proteins during apoptosis using a bio-orthogonal azidomyristate analog. FASEB J. *22*, 797–806. https://doi.org/10.1096/fj.07-9198com.

Mazid, M.A., Ward, C., Luo, Z., Liu, C., Li, Y., Lai, Y., Wu, L., Li, J., Jia, W., Jiang, Y., et al. (2022). Rolling back human pluripotent stem cells to an eight-cell embryo-like stage. Nature *605*, 315–324. https://doi.org/10.1038/s41586-022-04625-0.

Meyn, M.A., 3rd, Schreiner, S.J., Dumitrescu, T.P., Nau, G.J., and Smithgall, T.E. (2005). SRC family kinase activity is required for murine embryonic stem cell growth and differentiation. Mol. Pharmacol. *68*, 1320–1330. https://doi.org/10.1124/mol.104.010231.

Missbach, M., Jeschke, M., Feyen, J., Müller, K., Glatt, M., Green, J., and Susa, M. (1999). A novel inhibitor of the tyrosine kinase Src suppresses phosphorylation of its major cellular substrates and reduces bone resorption in vitro and in rodent models in vivo. Bone *24*, 437–449. https://doi.org/10.1016/s8756-3282(99)00020-4.

Moya-Jódar, M., Ullate-Agote, A., Barlabé, P., Rodríguez-Madoz, J. R., Abizanda, G., Barreda, C., Carvajal-Vergara, X., Vilas-Zornoza, A., Romero, J.P., Garate, L., et al. (2023). Revealing cell populations catching the early stages of human embryo development in naive pluripotent stem cell cultures. Stem Cell Rep. *18*, 64–80. https://doi.org/10.1016/j.stemcr.2022.11.015.

Patwardhan, P., and Resh, M.D. (2010). Myristoylation and Membrane Binding Regulate c-Src Stability and Kinase Activity. Mol. Cell Biol. *30*, 4094–4107. https://doi.org/10.1128/mcb.00246-10.

Petropoulos, S., Edsgärd, D., Reinius, B., Deng, Q., Panula, S.P., Codeluppi, S., Plaza Reyes, A., Linnarsson, S., Sandberg, R., and Lanner, F. (2016). Single-Cell RNA-Seq Reveals Lineage and X Chromosome Dynamics in Human Preimplantation Embryos. Cell *165*, 1012–1026. https://doi.org/10.1016/j.cell.2016.03.023.

Rhee, J.M., Pirity, M.K., Lackan, C.S., Long, J.Z., Kondoh, G., Takeda, J., and Hadjantonakis, A.K. (2006). In vivo imaging and differential localization of lipid-modified GFP-variant fusions in embryonic stem cells and mice. Genesis *44*, 202–218. https://doi.org/10.1002/dvg.20203.

Rossant, J., and Tam, P.P.L. (2017). New Insights into Early Human Development: Lessons for Stem Cell Derivation and Differentiation. Cell Stem Cell *20*, 18–28. https://doi.org/10.1016/j.stem.2016.12.004.

Schlaepfer, D.D., and Hunter, T. (1996). Evidence for in vivo phosphorylation of the Grb2 SH2-domain binding site on focal adhesion kinase by Src-family protein-tyrosine kinases. Mol. Cell Biol. *16*, 5623–5633. https://doi.org/10.1128/mcb.16.10.5623.

Shimizu, T., Ueda, J., Ho, J.C., Iwasaki, K., Poellinger, L., Harada, I., and Sawada, Y. (2012). Dual inhibition of Src and GSK3 maintains mouse embryonic stem cells, whose differentiation is mechanically regulated by Src signaling. Stem Cell. *30*, 1394–1404. https://doi.org/10.1002/stem.1119.

Smith, A. (2017). Formative pluripotency: the executive phase in a developmental continuum. Development *144*, 365–373. https://doi.org/10.1242/dev.142679.

Spassov, D.S., Ruiz-Saenz, A., Piple, A., and Moasser, M.M. (2018). A Dimerization Function in the Intrinsically Disordered N-Terminal Region of Src. Cell Rep. *25*, 449–463.e4. https://doi.org/10.1016/j.celrep.2018.09.035.

Takahashi, K., Tanabe, K., Ohnuki, M., Narita, M., Ichisaka, T., Tomoda, K., and Yamanaka, S. (2007). Induction of pluripotent stem cells from adult human fibroblasts by defined factors. Cell *131*, 861–872. https://doi.org/10.1016/j.cell.2007.11.019.

Takashima, Y., Guo, G., Loos, R., Nichols, J., Ficz, G., Krueger, F., Oxley, D., Santos, F., Clarke, J., Mansfield, W., et al. (2014). Resetting transcription factor control circuitry toward ground-state pluripotency in human. Cell *158*, 1254–1269. https://doi.org/10.1016/j.cell.2014.08.029.

Taubenschmid-Stowers, J., Rostovskaya, M., Santos, F., Ljung, S., Argelaguet, R., Krueger, F., Nichols, J., and Reik, W. (2022). 8C-like cells capture the human zygotic genome activation program in vitro. Cell Stem Cell *29*, 449–459.e6. https://doi.org/10.1016/j.stem.2022.01.014.

Tesar, P.J., Chenoweth, J.G., Brook, F.A., Davies, T.J., Evans, E.P., Mack, D.L., Gardner, R.L., and McKay, R.D.G. (2007). New cell lines from mouse epiblast share defining features with human embryonic stem cells. Nature *448*, 196–199. https://doi.org/10.1038/nature05972.

Theunissen, T.W., Powell, B.E., Wang, H., Mitalipova, M., Faddah, D.A., Reddy, J., Fan, Z.P., Maetzel, D., Ganz, K., Shi, L., et al. (2014). Systematic identification of culture conditions for induction and maintenance of naive human pluripotency. Cell Stem Cell *15*, 471–487. https://doi.org/10.1016/j.stem.2014.07.002.

Thinon, E., Serwa, R.A., Broncel, M., Brannigan, J.A., Brassat, U., Wright, M.H., Heal, W.P., Wilkinson, A.J., Mann, D.J., and Tate, E.W. (2014). Global profiling of co- and post-translationally N-myristoylated proteomes in human cells. Nat. Commun. *5*, 4919. https://doi.org/10.1038/ncomms5919.

Thomson, J.A., Itskovitz-Eldor, J., Shapiro, S.S., Waknitz, M.A., Swiergiel, J.J., Marshall, V.S., and Jones, J.M. (1998). Embryonic stem cell lines derived from human blastocysts. Science (New York, N.Y.) *282*, 1145–1147. https://doi.org/10.1126/science.282.5391.1145.

Yanagida, A., Spindlow, D., Nichols, J., Dattani, A., Smith, A., and Guo, G. (2021). Naive stem cell blastocyst model captures human embryo lineage segregation. Cell Stem Cell *28*, 1016–1022.e4. https://doi.org/10.1016/j.stem.2021.04.031.

Yang, S.H., Shrivastav, A., Kosinski, C., Sharma, R.K., Chen, M.H., Berthiaume, L.G., Peters, L.L., Chuang, P.T., Young, S.G., and Bergo, M.O. (2005). N-myristoyltransferase 1 is essential in early mouse development. J. Biol. Chem. *280*, 18990–18995. https://doi.org/10.1074/jbc.M412917200.

Ying, Q.L., Wray, J., Nichols, J., Batlle-Morera, L., Doble, B., Woodgett, J., Cohen, P., and Smith, A. (2008). The ground state of embryonic stem cell self-renewal. Nature *453*, 519–523. https://doi.org/10.1038/nature06968.

Yu, J., Vodyanik, M.A., Smuga-Otto, K., Antosiewicz-Bourget, J., Frane, J.L., Tian, S., Nie, J., Jonsdottir, G.A., Ruotti, V., Stewart, R., et al. (2007). Induced pluripotent stem cell lines derived from human somatic cells. Science *318*, 1917–1920. https://doi.org/10.1126/science.1151526.

Yu, L., Wei, Y., Duan, J., Schmitz, D.A., Sakurai, M., Wang, L., Wang, K., Zhao, S., Hon, G.C., and Wu, J. (2021). Blastocyst-like structures generated from human pluripotent stem cells. Nature *591*, 620–626. https://doi.org/10.1038/s41586-021-03356-y.

Yuan, M., Song, Z.H., Ying, M.D., Zhu, H., He, Q.J., Yang, B., and Cao, J. (2020). N-myristoylation: from cell biology to translational medicine. Acta Pharmacol. Sin. *41*, 1005–1015. https://doi.org/10.1038/s41401-020-0388-4.

Stem Cell Reports, Volume *20*

Supplemental Information

Inhibition of N-myristoyltransferase in pluripotent stem cells promotes the naive state in mice and elicits trophectoderm and primitive endoderm markers in humans

Junko Yoshida, Hitomi Watanabe, Kaori Yamauchi, Takumi Nishikubo, Ayako Isotani, Satoshi Ohtsuka, Hitoshi Niwa, Yuki Kawamoto, Hidenori Akutsu, Akihiro Umezawa, Hirofumi Suemori, Yasuhiro Takashima, Hideo Matsuda, Gen Kondoh, Junji Takeda, and Kyoji Horie

Inhibition of N-myristoyltransferase in Pluripotent Stem Cells Promotes the Naive State in Mice and Elicits Trophectoderm and Primitive Endoderm Markers in Humans

Junko Yoshida, Hitomi Watanabe, Kaori Yamauchi, Takumi Nishikubo, Ayako Isotani, Satoshi Ohtsuka, Hitoshi Niwa, Yuki Kawamoto, Hidenori Akutsu, Akihiro Umezawa, Hirofumi Suemori, Yasuhiro Takashima, Hideo Matsuda, Gen Kondoh, Junji Takeda, and Kyoji Horie

This Supplementary File includes:

- Supplementary Figures 1-6 and their legends
- Supplementary Table 1. PCR primers used in this study
- Supplemental Experimental Procedures

The following Supplementary Tables are provided in Excel format:

- Supplementary Table 2. RNA-seq analysis of *Nmt1*-mutant and wild-type mESCs, related to Figure 1E
- Supplementary Table 3. RNA-seq analysis of naive hiPSCs with or without NMT inhibitor, related to Figure 5D

Supplementary Figure 1

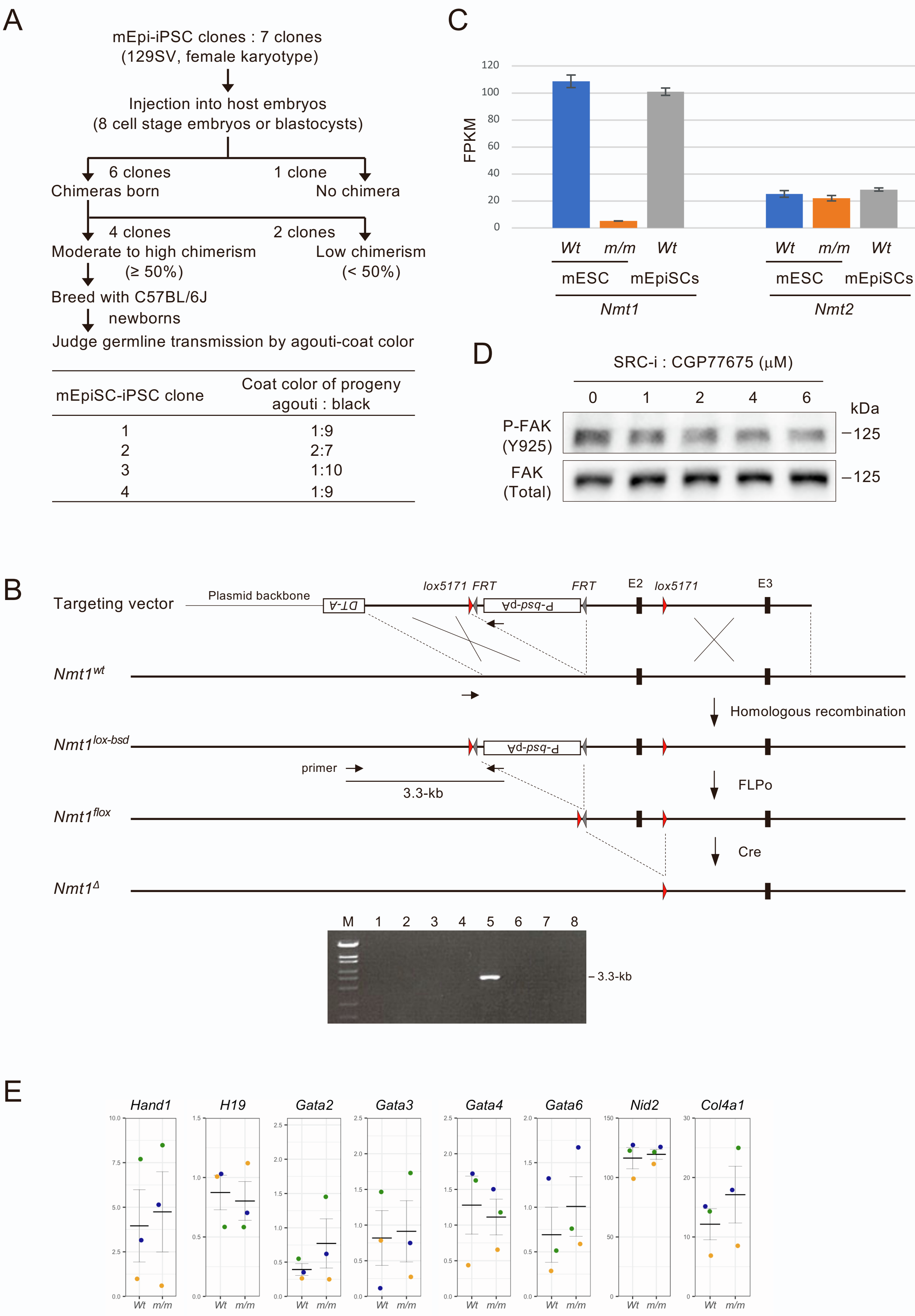

Supplementary Figure 1. Effect of NMT suppression on mouse cells, related to Figures 1-5.

(A) Generation of chimeric mice from mEpi-iPSC clones and evaluation of germline transmission rate, related to Figure 2J.

(B) Generation of the conditional allele at the *Nmt1* locus by gene targeting, related to Figures 3A and 3B. Gene targeting was conducted to the wild-type *Nmt1* allele (*Nmt1*^{wt}) of the heterozygous gene-trapped clone (*Nmt1*$^{m/wt}$). Homologous recombination between the targeting vector and the *Nmt1*^{wt} allele was screened by PCR as shown in the gel image. FLPo-mediated excision of the P-*bsd*-pA cassette was identified by blasticidin S-sensitivity of the single cell-derived clones. Detection of Cre-mediated recombination was performed by 4-hydroxytamoxifen administration as shown in Figures 3A and 3B. *DT-A*, diphtheria toxin A fragment; P, CAG promoter; *bsd,* blasticidin S deaminase gene; pA, bovine growth hormone polyadenylation signal; E, exon; M, size marker (λ/StyI digest).

(C) Expression levels of *Nmt1* and *Nmt2* in mESCs and mEpiSCs as determined by RNA-seq. Data are presented as the mean ± SEM of FPKM (fragments per kilobase per million mapped reads) from three independent replicates.

(D) Western blot analysis of replicate 2 from Figure 4A, demonstrating the dose-dependent inhibitory effect of the SRC inhibitor on FAK-phosphorylation at Y925 in mEpiSCs. Quantification of band intensities is shown in Figure. 4A.

(E) Effect of *Nmt1* disruption on mouse orthologs corresponding to the human trophectoderm and primitive endoderm marker genes shown in Figure 5D. No significant difference was observed in the expression of mouse markers shown here between *Wt* and *Nmt1*$^{m/m}$ based on statistical analysis. Note that the data for *Gata4* and *Gata6* are the same as those shown in Figure 1E.

Supplementary Figure 2

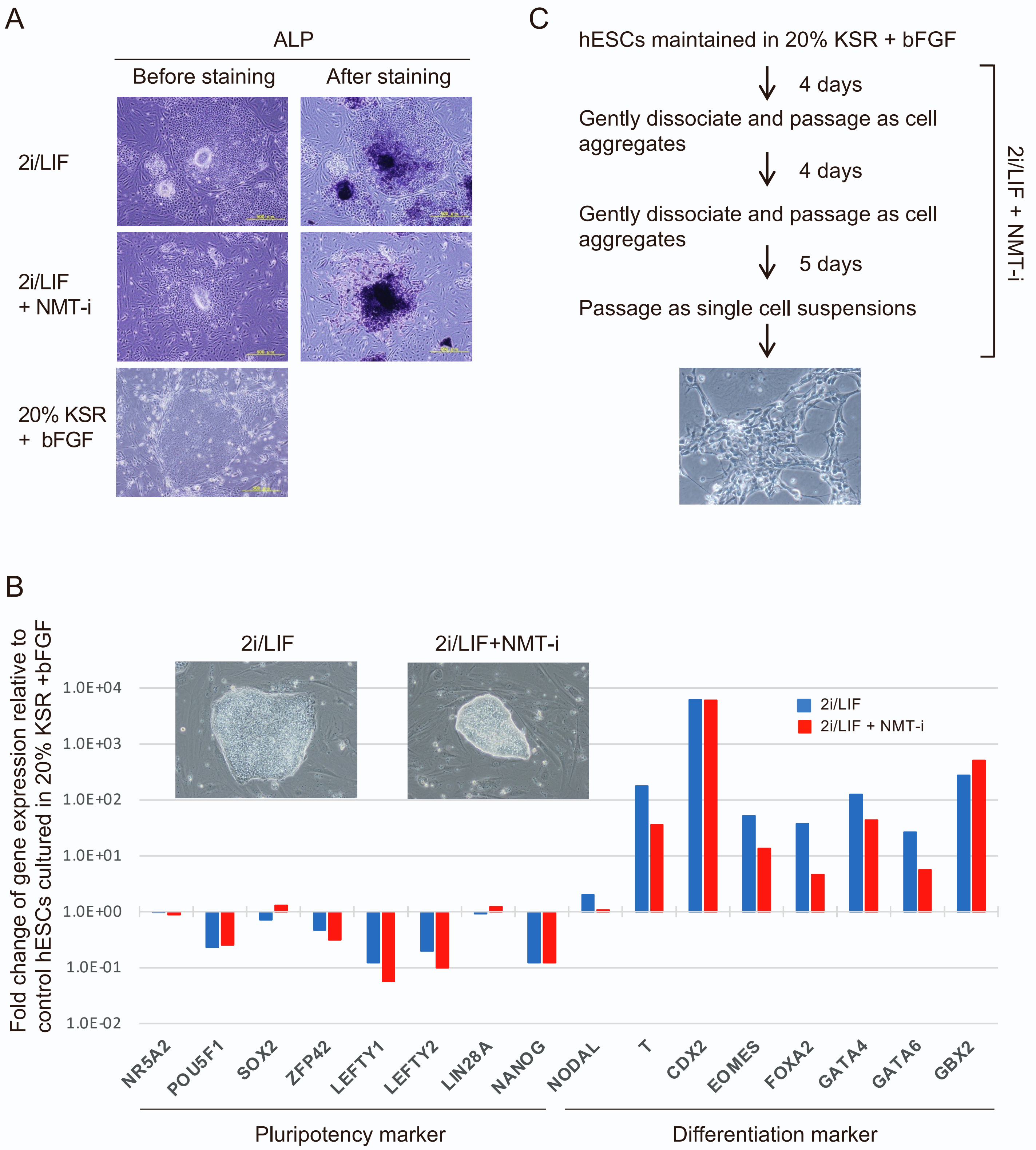

Supplementary Figure 2. Culture condition of 2i/LIF + NMT inhibitor does not support the undifferentiated state of human pluripotent stem cells.
(A) ALP-staining of hiPSCs at passage 2 in 2i/LIF + NMT inhibitor.
(B) Gene expression in hESCs after 4 day-culture in 2i/LIF or 2i/LIF + NMT inhibitor relative to KSR +bFGF.
(C) Morphology of hESCs after long-term culture in 2i/LIF + NMT inhibitor.

Supplementary Figure 3

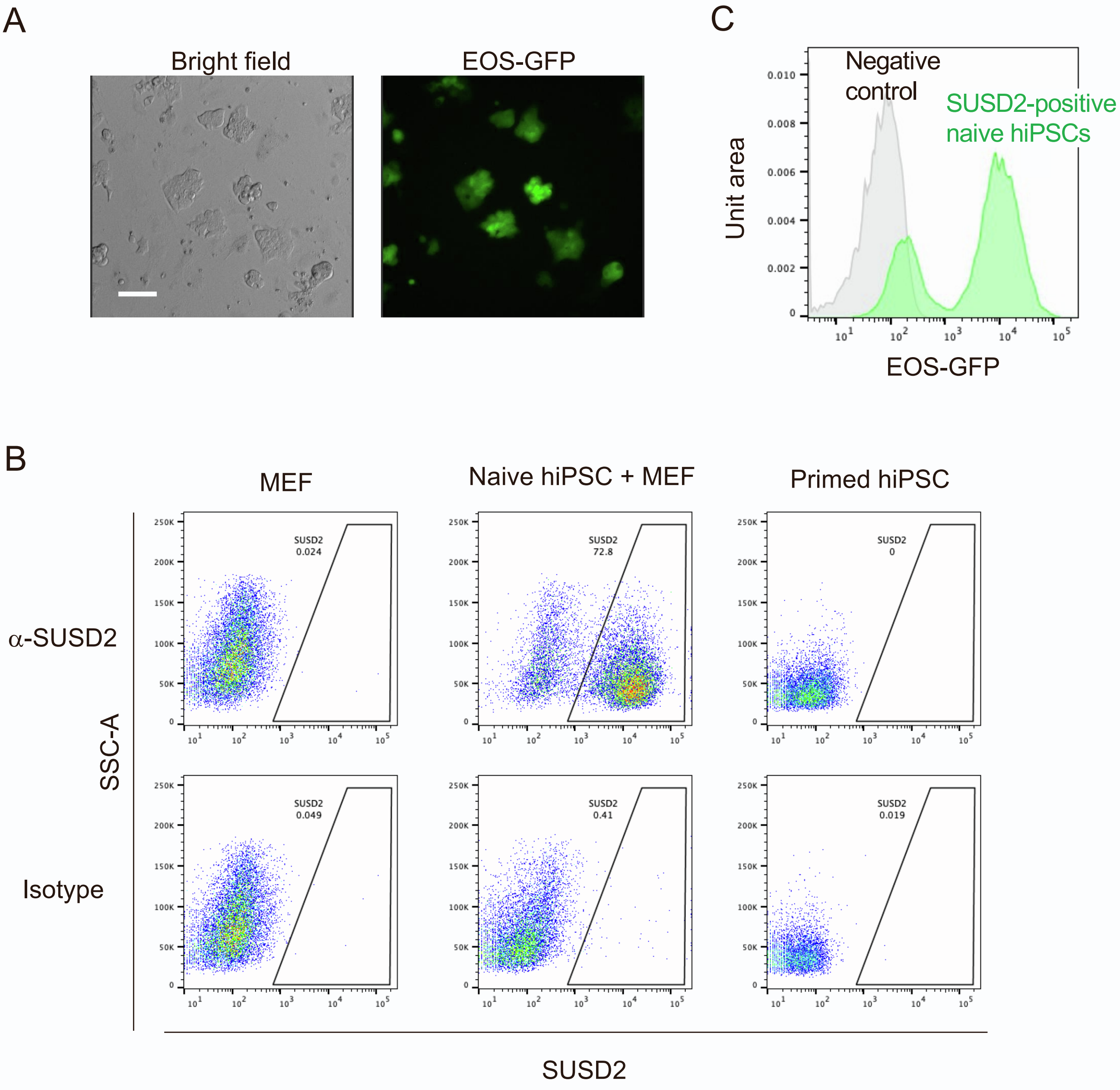

Supplementary Figure 3. Naive hiPSCs generated using t2iLGö medium, related to Figure 5.

(A) Morphology of naive hiPSCs and EOS-GFP reporter expression in t2iLGö medium. Scale bar: 100 µm.

(B) Expression of naive-state marker SUSD2. MEF feeders could be excluded from the naive hiPSC culture by gating the expression of SUSD2.

(C) The expression of EOS-GFP in the SUSD2-positive population shown in (B).

Supplementary Figure 4

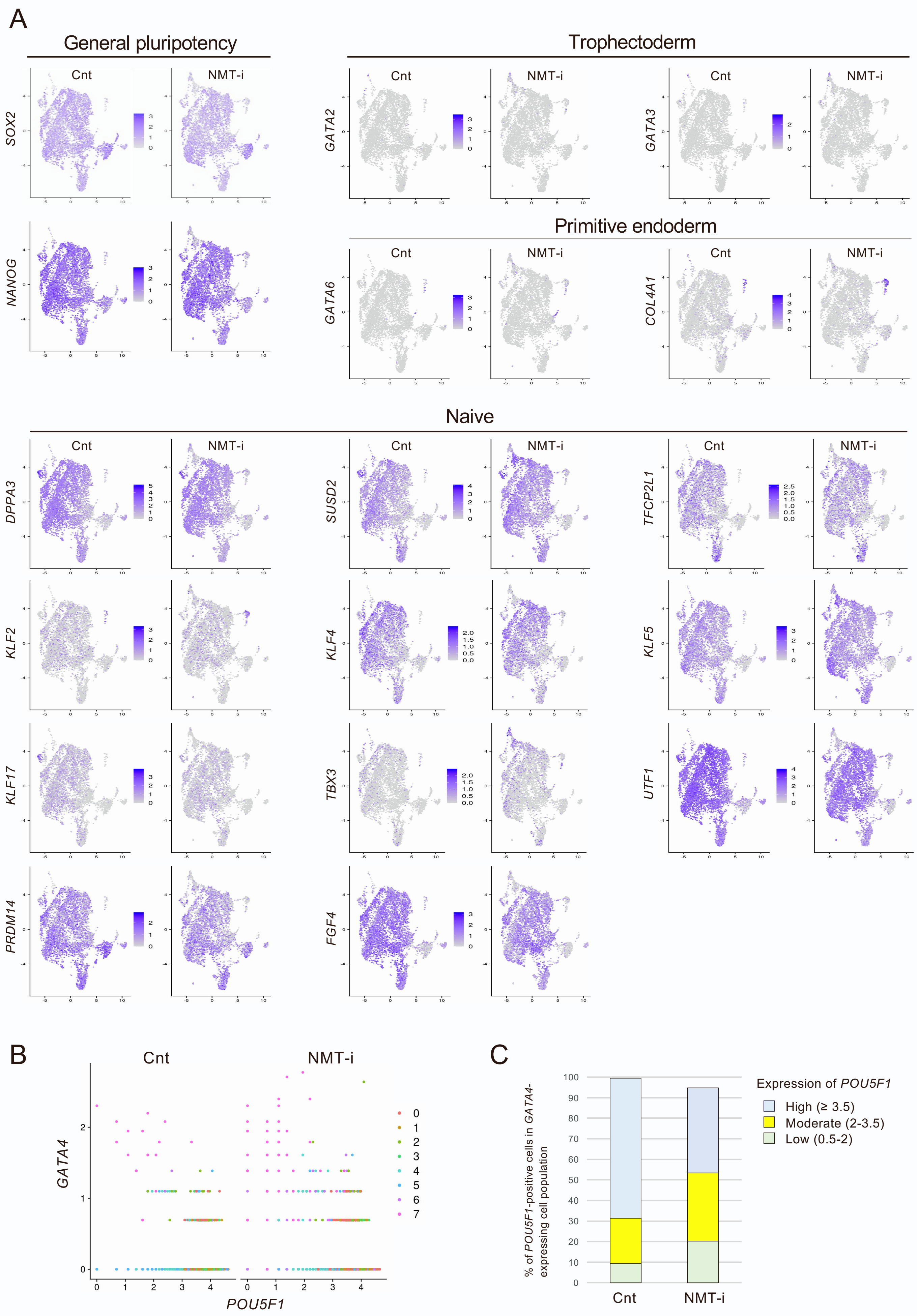

Supplementary Figure 4. Effect of NMT inhibitor on the expression of developmental markers in naive hiPSCs analyzed by scRNA-seq, related to Figure 6.

(A) UMAPs showing the expression of developmental markers.

(B) Scatter plots of *POU5F1* and *GATA4* co-expression, colored by clusters 0–7 as in Figure 6A.

(C) Quantification of co-expression shown in (B), stratified by *POU5F1* expression levels.

Supplementary Figure 5

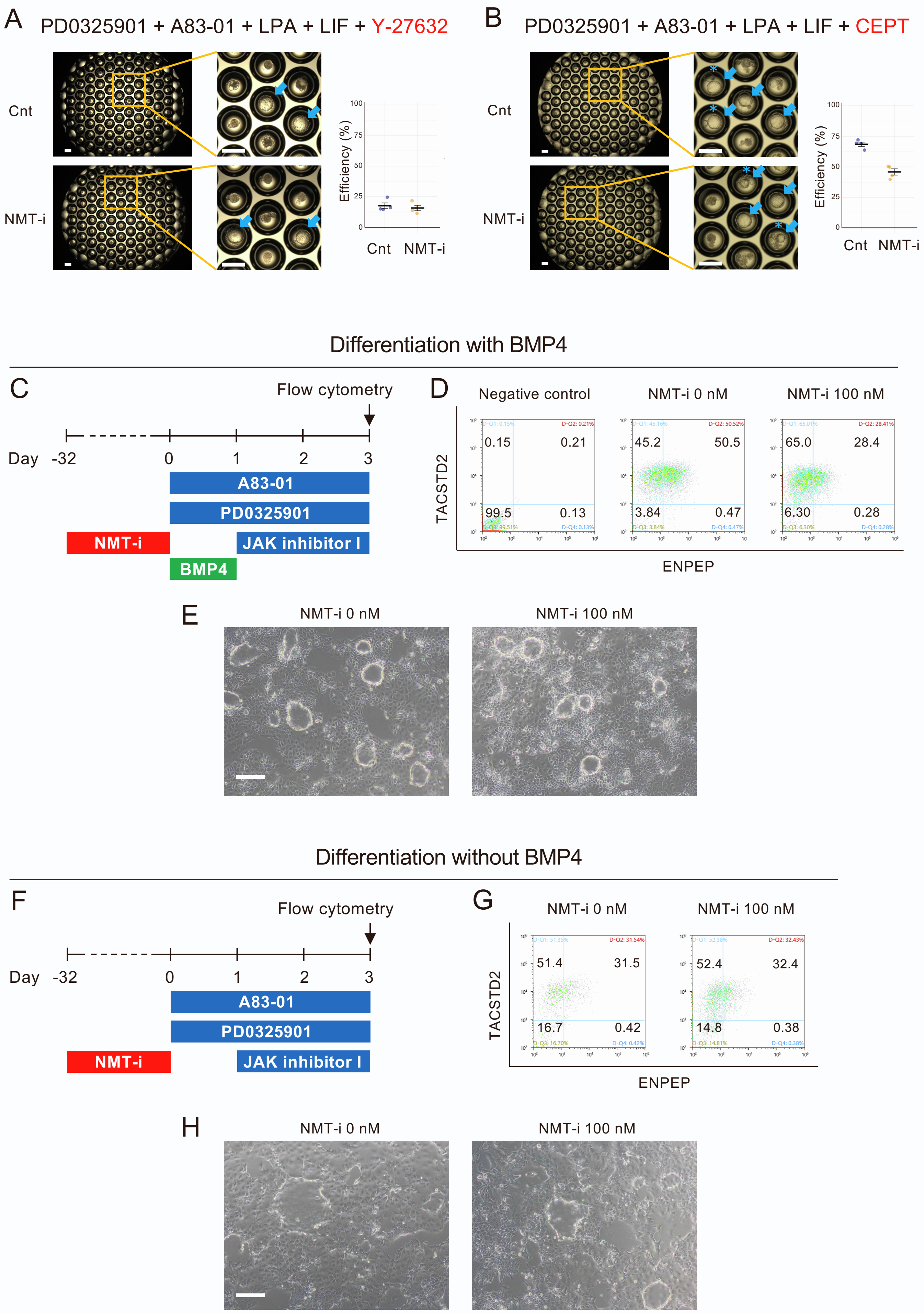

Supplementary Figure 5. Effect of the NMT inhibitor on blastoid formation and trophectoderm differentiation, related to Figure 6.

(A, B) Blastoid formation assay. Phase-contrast images of human blastoids formed in an Elplasia microwell plate (Corning) and the efficiency of blastoid formation judged by cyst formation. To enhance the viability of cell aggregates, Y-27632 was used in (A), while the CEPT cocktail was used in (B). Representative images of an Elplasia well, each containing approximately 90 microwells, are shown on the left. Blue arrows indicate microwells in which blastoid formation occurred. Multi-cystic structures were frequently observed in the presence of the CEPT cocktail (B), as indicated by asterisks. The efficiency of blastoid formation from four wells is presented as mean ± SEM. NMT-i, NMT inhibitor; Cnt, control without NMT-i. Scale bar: 400 μm.

(C-H) Trophectoderm differentiation assay. (C, F) Schematic representation of the protocol for trophectoderm induction with (C) and without (F) BMP4. The NMT inhibitor was added prior to the differentiation induction but omitted during the differentiation process. (D, G) Flow cytometry analysis of trophectoderm markers on day 3. Gating for the expression of ENPEP and TACSTD2 was established based on unstained cells differentiated in the presence of BMP4, as shown in (D), and the same gating was applied in (G). (E, H) Phase-contrast image of differentiated cells on day 3. Scale bar: 500 μm.

Supplementary Figure 6

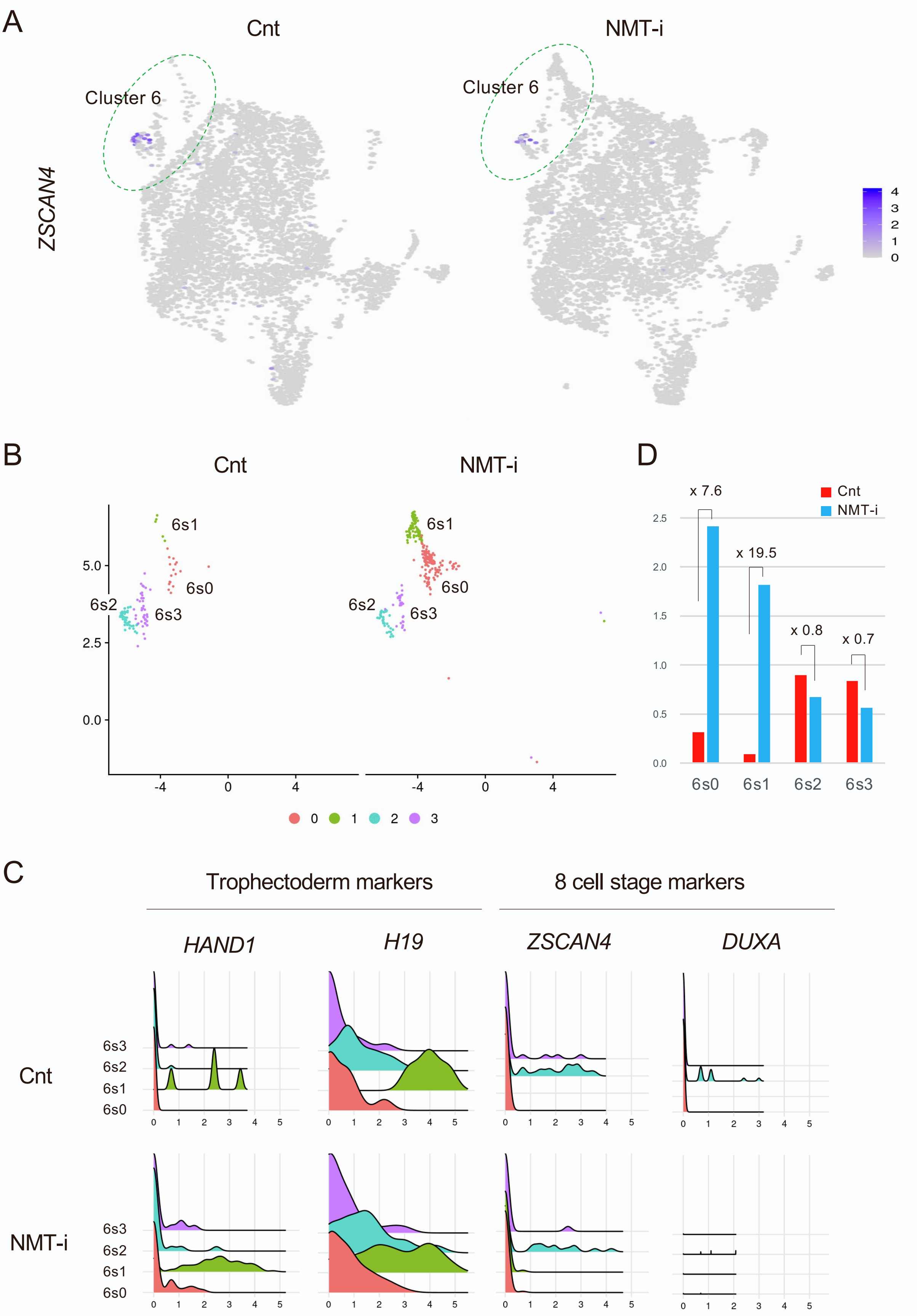

Supplementary Figure 6. Analysis of eight-cell-stage marker expression by scRNA-seq, related to Figure 6.

(A) UMAP visualization showing the expression of *ZSCAN4*.
(B) Subclustering of cluster 6.
(C) Ridge plot showing the expression patterns of trophectoderm and eight-cell-stage markers.
(D) Proportions of cell populations in each subcluster identified in (B).

Supplementary Table 1. PCR primers

Construction of the *Nmt1* -targeting vector	
Nmt1-L-Upp1	cgtttaaacggcgcgccAGCTCCAACTCCAGTACGTGCTGTCTATTC
Nmt1-L-Low1	cttcgtataatgtgtactatacgaagttatGTTAACTGGTCAGGGGCTAGGTCGAGGCAC
Nmt1-L-Upp2	cttcgtatagtacacattatacgaagttatTGACGAGTATTTTGACTGGTTTGCTCTTCT
Nmt1-L-Low2	taccgtcgaccttaattaaATCCCACGCTGGTCTCATTTGGACATTTTA
Nmt1-S-Upp1	agttctagagcggccgcCACGGCCTTGCTGTGAGAGCACGTGGGAGG
Nmt1-S-Low1	cactgctcgacatttaaatGTAGCGGATGCTTCCTGCCTCCCAAGGACA
Screening of the *Nmt1* -targeted clones	
Nmt1-scn1	GCCTCCCAAGGACAGATTCCATCTCATTCT
bsd3-1	AATTGCTGCCCTCTGGTTATGTGTGGGAGG
Detection of the Cre/*loxP* -mediated deletion of the *Nmt1* exon 2 region	
Nmt1-Flpo-Scn-F1	TTTTACATTGTCCCCACCCCAGCCT
Nmt1-Flpo-Scn-R1	TTAGGCTGCTTTTCCCCCAGTAAGT
Nmt1-Cre-Scn-R1	CATACTGTGCTGTCAATGTACTGTG
qRT-PCR	
mFgf5-RP-F3	AAGTAGCGCGACGTTTTCTTC
mFgf5-RP-R3	CTGGAAACTGCTATGTTCCGAG
Dppa3-F1	GAGGACGCTTTGGATGATACAGACG
Dppa3-R1	CAACAAAGTGCGGACCCTTCTCTTG
Actb-F1	CAGGGTGTGATGGTGGGAATGGGTCAGAAG
Actb-R1	TACGTACATGGCTGGGGTGTTGAAGGTCTC

SUPPLEMENTAL EXPERIMENTAL PROCEDURES

Cell Line and Cell Culture

mESCs were cultured either in serum-containing medium or serum-free 2i medium depending on the purpose of the experiment. The serum-containing medium was composed of KnockOut DMEM (Cat. 10829018, Thermo Fisher Scientific) supplemented with 20% fetal bovine serum, non-essential amino acids (Cat. 11140050, Thermo Fisher Scientific), penicillin-streptomycin-glutamine (Cat. 10378016, Thermo Fisher Scientific), 0.1 mM of 2-mercaptoethanol (Cat. M3148, Sigma) and 1,000 U/ml of leukemia inhibitory factor (LIF) (Cat. ESG1107, Merck Millipore), and mitomycin C (MMC)-treated MEFs were used as feeder cells. The serum-free 2i medium was composed of N2B27 (Guo et al., 2017) supplemented with 1 μM of MEK inhibitor PD0325901 (Cat. Axon1408, Axon Medchem) and 3 μM of GSK3 inhibitor CHIR99021 (Cat. Axon1386, Axon Medchem). We routinely added LIF to the serum-free medium (2i/LIF), except in the experiment shown in Fig. 1D.

mEpiSCs were cultured in DMED/F12 (Cat. 11320033, Thermo Fisher Scientific) supplemented with 20% KnockOut Serum Replacement (KSR) (Cat. 10828028, Thermo Fisher Scientific), non-essential amino acids, sodium pyruvate, 0.1 mM of 2-mercaptoethanol, 5 ng/ml of bFGF (Cat. 16100102, Katayama Chemical Industries) and 10 ng/ml of activin A (Cat. 120-14, PeproTech). MMC-treated MEFs were used as feeder cells.

Naive-state hiPSCs were established from the primed-state adipocyte-derived hiPSCs (Takashima et al., 2014). We first introduced the EOS-GFP reporter vector (Hotta et al., 2009) into the primed-state hiPSCs and then induced conversion to the naive state by the protocol previously described (Guo et al., 2017). Naive hiPSCs were maintained in t2iLGö or PXGL media. t2iLGö medium consists of N2B27 (Ndiff227; Cat. Y40002, Takara Bio) with 1 μM of PD0325901 (Cat. 4192, Tocris), 1 μM of CHIR99021 (Cat. SML1046, Sigma-Aldrich), 10 ng/mL of recombinant human LIF (Cat. 300-05, Peprotech), and 2 μM of Gö6983 (Cat. 2285, Tocris), as previously described (Takashima *et al.*, 2014). Naive hiPSCs were passaged every 3-5 days using Accutase (Cat. A6964, Sigma-Aldrich). PXGL medium consists of N2B27 with 1 μM of PD0325901, 2 μM of XAV939 (Cat.X3004, Sigma-Aldrich), 2 μM of Gö6983, and 10 ng/mL of recombinant human LIF (Cat. 300-05, Peprotech), as previously described (Bredenkamp et al., 2019).

The primed-state hiPSC line MRC5iPS was generated from the human fetal lung fibroblast cell line MRC-5 (Jacobs et al., 1970) by retroviral transduction of reprogramming factors (*OCT3/4*, *SOX2*, *KLF4*, *c-MYC*) (Takahashi et al., 2007). ALP activity was detected with VECTOR Red Alkaline Phosphatase Substrate Kit I (Cat. SK-5100, Vector Laboratories) according to the manufacturer's instructions.

The primed-state hESC line KhES-1 was maintained as previously described (Suemori et al., 2006). To test the effect of the NMT inhibitor DDD85646 (Frearson et al., 2010) (provided by Dr. Paul Wyatt, University of Dundee, UK) on prime-state hESC, KhES-1 was passaged in 2i/LIF with or without the inhibitor and analyzed for gene expression.

For real-time PCR analysis and bulk RNA-seq of mESCs, MEF feeder cells were removed by plating cells on a gelatin-coated dish for 30 min during the passaging and collecting unattached cells. For real-time PCR analysis of the hESCs, MEF feeder cells were removed by separating them from clumps of hESCs under gravity sedimentation during the passaging. For scRNA-seq of hiPSCs, MEF feeder cells were not physically removed during cell culture; instead, their contributions were computationally excluded, as described later.

Isolation of *Nmt1*-revertant Clones

Nmt1-homozygous mESCs were transfected with pCAGGS-FLPo-IRESpuro (Kranz et al., 2010) using TransFast (Cat. E2431, Promega) to excise the *FRT*-flanked gene trap cassette. Three days after

transfection, mESCs were sparsely plated on MMC-treated MEFs for single cell cloning. One week later, single cell-derived mESC colonies were picked and divided into three culture conditions: (1) with G418 (Geneticin; Cat. 10131027, Thermo Fisher Scientific), (2) with puromycin (Cat. P7255, Sigma-Aldrich), and (3) without drug selection. The parental *Nmt1*-homozygous mESC clone expresses the neomycin-resistance gene and the puromycin-resistance gene from each allele. Therefore, reversion of both alleles confers sensitivity to both G418 and puromycin.

Immunostaining

For the immunostaining of OCT3/4, NANOG and KLF4, cells were fixed with 4% paraformaldehyde (Cat. 02890-45, Nacali Tesque) in PBS for 10 min, permeabilized with 0.2% Triton X-100 (Cat. 35501, Nacali Tesque) for 10 min, and subjected to blocking with 1% bovine serum albumin (BSA) (Cat A5611, Sigma-Aldrich) in PBS for 20 min. The following primary antibodies were used: anti-OCT3/4 mouse monoclonal antibody (1:300, clone c-10, Cat. Sc-5279, Santa Cruz Biotechnology), anti-NANOG rabbit polyclonal antibody (1:200, Cat. RCAB002P-F, ReproCELL), and anti-KLF4 rabbit monoclonal antibody (1:1000, Cat. ab214666, Abcam). Alexa Fluor 488-conjugated goat anti-mouse IgG (Cat. A-11001, Thermo Fisher Scientific) was used for OCT3/4 and Alexa Fluor 594-conjugated goat anti-rabbit IgG (Cat. A-11012, Thermo Fisher Scientific) for NANOG and KLF4 as a secondary antibody, respectively, and DAPI (Cat. 62248, Thermo Fisher Scientific) was used for counterstaining when needed. For the immunostaining of SUSD2, the cells were incubated with APC-conjugated anti-SUSD2 antibody (1:20, clone W5C5, Cat. 327401, BioLegend) for 30 min in culture medium. The cells were washed three times with PBS and analyzed by FACSAria II (Becton, Dickinson and Company) or SH800 (SONY).

Converting mEpiSCs to Naive-state mEpi-iPSCs

mEpiSCs (2×10^4) were plated onto MMC-treated MEFs in N2B27-based medium supplemented with bFGF and activin A. The next day (day 0), the medium was changed to an N2B27-based 2i/LIF medium with or without the NMT inhibitor (DDD85646) or the SRC inhibitor (CGP77675) (Cat. 21089, Cayman Chemical). On day 2, the cells were passaged at 1:20 onto MEFs in the same medium. On day 7, the cells were passaged at 1:100 onto MEFs in 2i/LIF medium without inhibitors. On day 15, the number of dome-shaped colonies were counted.

Generating Chimeric Mice and Assessing Germline Transmission

After generating mEpi-iPSCs from mEpiSCs in serum-free 2i/LIF medium, we cultured them in serum-containing medium on MMC-treated MEFs for several days and injected them into eight-cell-stage embryos or blastocysts. We used ICR or BDF1-derived embryos as a host. As the parental mEpiSCs were derived from a female 129SV mouse strain, we selected female agouti-colored chimeric mice and crossed them with male C57BL/6J mice to test germline transmission. The germline transmission was assessed by the agouti coat-color of the progeny.

Generating the Conditional Allele at the *Nmt1* Locus

To conduct a conditional knockout of the *Nmt1* gene, we performed gene targeting and floxed the second exon of the *Wt* allele of the *Nmt1*-heterozygous mESC clone that we previously obtained by gene trapping (Horie et al., 2011). The targeting vector was constructed as follows, and the PCR primer sequences are listed in Supplementary Table 1. We first PCR-amplified a genomic fragment of the first intron of the *Nmt1* gene, using primer pairs Nmt1-S-Upp1 and Nmt1-S-Low1, using the genomic DNA of the mESC line KY1.1 (Yagita et al., 2010) as a template, digested with NotI and SwaI, and cloned into the NotI-SwaI site of the pMulti-Lox5171-FRT-CAG-bsd-pA-FRT (unpublished), which contains the *FRT*-flanked blasticidin S deaminase expression cassette and a

single copy of the *lox5171* site at this cloning site, resulting in the pMulti-Lox5171-FRT-CAG-bsd-pA-FRT-5HR. We next amplified the *Nmt1* genomic region spanning from the first intron to the second intron, using the primers Nmt1-L-Upp2 and Nmt1-L-Low2, and the genomic region spanning from the second intron to the third intron, using the primers Nmt1-L-Upp1 and Nmt1-L-Low1. These fragments have overlapped sequences containing the *lox5171* site introduced by PCR primers. We therefore conducted fusion PCR, using the mixture of these fragments as a template, and using primers Nmt1-L-Upp1 and Nmt1-L-Low2. The fused fragments were digested with AscI and PacI and cloned into the AscI-PacI site of the pMulti-Lox5171-FRT-CAG-bsd-pA-FRT-5HR, resulting in the targeting vector pMulti-Lox5171-FRT-CAG-bsd-pA-FRT-5HR-3HR. The targeting vector was linearized with AscI, and 25 μg of the targeting vector was transfected into 1×10^7 of *Nmt1*-heterozygous cells (Horie *et al.*, 2011) by electroporation (240 V, 500 μF) with Gene Pulser II (Bio-Rad). One week later, blasticidin S-resistant clones were picked and screened for homologous recombinants by primers Nmt1-scn1 and bsd3-1. Targeted clones were transfected with pCAGGS-FLPo-IRESpuro to remove the bsd cassette and generate the floxed *Nmt1* allele. The parental mESC line contained the *ERT2-iCre-ERT2* gene at the *Rosa26* locus (Horie *et al.*, 2011). Therefore, the conditional knockout of the *Nmt1* gene was achieved by treating cells with 4-hydroxytamoxifen (4HT) (Cat. H6278, Sigma-Aldrich). To confirm that the conditional allele was correctly generated, we treated mESCs with 1 μM of 4HT overnight and analyzed Cre-mediated recombination by PCR, using primers Nmt1-Flpo-Scn-F1 and Nmt1-Flpo-Scn-R1 for detecting the undeleted allele, and primers Nmt1-Flpo-Scn-F1 and Nmt1-Cre-Scn-R1 for detecting the deleted allele. We also conducted PCR by mixing all three primers in order to suppress the amplification of MEF-derived genomic DNAs that could not be eliminated by plating on a gelatin-coated dish.

Converting mESCs to mEpiSC-like Primed-state Cells

mESCs carrying the floxed *Nmt1* allele were converted into mEpiSC-like primed-state cells according to the published protocol (Guo et al., 2009). Briefly, mESCs were cultured in N2B27 medium supplemented with 12 ng/ml of bFGF and 20 ng/ml of activin A on a dish coated with fibronectin (Cat. 354008, Corning), and passaged at every 3–5 days. The morphology of the cell clusters became gradually flatter. We analyzed the primed-state marker *Fgf5* and the naive state marker *Dppa3* by qRT-PCR at passage nine to confirm conversion into the primed state.

Converting mEpiSC-like Primed-state Cells to Naive-state mEpi-iPSCs by the Conditional Knockout of *Nmt1*

mEpiSC-like primed-state cells were plated onto MMC-treated MEFs in N2B27-based medium supplemented with 12 ng/ml of bFGF and 20 ng/ml of activin A and treated with 1 μM of 4HT for 12 hours to induce the Cre-mediated deletion of the *Nmt1* allele. The same amount of ethanol was added to the medium as a mock. The cells were maintained in the same N2B27/bFGF/activin A medium for another five days to reduce the intracellular concentration of NMT1 protein. Then, the cells were plated onto MMC-treated MEFs in the same medium at the concentration of 2×10^4 cells per well. The next day (day 0), the medium was changed to 2i/LIF medium to induce conversion to the naive state. On day 7, the cells were passaged at 1:70 onto MEFs. On day 15, the cells were stained for ALP activity, using VECTOR Red Alkaline Phosphatase Substrate Kit I (Cat. SK-5100, Vector Laboratories), according to the manufacturer’s instructions, and the number of ALP-positive colonies were counted.

Quantitative RT-PCR (qRT-PCR)

To quantify gene expression in the mouse cells, the total RNA was extracted with RNeasy Plus Mini Kit (Cat. 74136, Qiagen) and reverse-transcribed with SuperScript III (Cat. 18080044, Thermo Fisher

Scientific), using random primers (Cat. C1181, Promega). The expression levels of mRNAs encoding *Dppa3*, *Fgf5*, and *Actb* were quantified by real-time PCR, using the LightCycler FastStart DNA Master SYBR Green I kit (Cat. 12239264001, Roche Diagnostics) on the LightCycler (Roche Diagnostics). The primer pairs are listed in Supplementary Table 1. The amplification conditions for *Dppa3* and *Fgf5* were 95 °C for 10 min for one cycle, followed by 40 cycles of denaturation at 95 °C for 10 sec, annealing at 56 °C for 5 sec and extension at 72 °C for 20 sec. The amplification conditions for *Actb* were the same except that the annealing temperature was 55 °C. The quantity of each transcript was measured from a standard curve, and the amounts of *Dppa3* and *Fgf5* transcript were normalized to *Actb* transcript levels.

To quantify gene expression in hESCs (KhES-1), the total RNA was extracted with RNeasy Micro Kit (Cat. 74004, Qiagen) and reverse-transcribed with an RT^2 First Strand Kit (Cat. 330404, Qiagen). The expression levels of the mRNAs were quantified using the Human Embryonic Stem Cell RT^2 Profile™ PCR Array (Cat. PAHS-081, Qiagen) and RT^2 SYBR Green qPCR Master Mix (Cat. 330504, Qiagen). All procedures followed the manufacturer's instructions.

Microscopic Analysis of myrVenus Reporter Localization

The myrVenus reporter (Rhee et al., 2006) was cloned into the piggyBac transposon vector (Cadinanos and Bradley, 2007) under the control of the CAG promoter (Niwa et al., 1991) and with the IRES-bsd selection cassette. This vector was introduced into mESCs by co-transfecting the piggyBac expression vector mPB (Cadinanos and Bradley, 2007), using TransFast transfection reagent, and selected by 30 μg/ml of blasticidin S (Cat. KK-400, Kaken Pharmaceutical). We stained the plasma membrane using CellMask Deep Red Plasma Membrane Stain (Cat. C10046, Thermo Fisher Scientific) according to the manufacturer's instructions and fixed the cells with 4% of paraformaldehyde. We captured the fluorescent images and conducted a line-plot analysis of the fluorescence signal using DeltaVision Elite (Cytiva).

Western blot analysis

To determine the effect of the *Nmt1* knockout on the localization of the myrVenus reporter, membrane and cytosol fractions were prepared from mESCs using Minute Plasma Membrane Protein Isolation and Cell Fractionation Kit (Cat. SM-005, Invent Biotechnologies, Inc.) following the manufacturer's protocol. Each fraction was homogenized in 1× Laemmli sample buffer (Laemmli, 1970), and protein concentrations were determined using Pierce 660nm Protein Assay Kit (Cat. 22662, Thermo Fisher Scientific) supplemented with Ionic Detergent Compatibility Reagent for Pierce 660nm Protein Assay Reagent (Cat. 22663, Thermo Fisher Scientific). On average, we obtained a 5.5-fold higher protein yield in the membrane fraction compared to the cytosol fraction. Therefore, we loaded 1.8 μg of membrane fraction proteins and 10 μg of cytosol fraction proteins on 10% Mini-PROTEAN TGX Precast Protein Gels (Cat. 4561035, Bio-Rad) to ensure that the ratio of membrane to cytosol proteins loaded on the gel reflected the protein yield from each fractionation. Separated proteins were transferred to a PVDF membrane (Cat. 170-4156, Bio-Rad). The enrichment of membrane proteins was assessed using an antibody against Na/K-ATPase (1:1000, Cat. 3010, Cell Signaling), followed by incubation with an anti-rabbit horseradish peroxidase-linked antibody (1:2000, Cat. 7074, Cell Signaling). Protein detection was performed using Chemi-Lumi One Super (Cat. 02230, Nacalai Tesque) on the FUSION-SOLO.7S.EDGE imaging system (Vilber Bio Imaging). The antibodies were stripped, and the localization of the myrVenus reporter was assessed using an anti-GFP antibody (1:2500, Cat. 598, MBL International Corp.). The remaining procedure followed the method described above. The antibodies were then stripped again, and the enrichment of cytosolic proteins was investigated using a primary antibody against GAPDH (1:3000, Cat. 2118, Cell Signaling). Band

intensities were determined using Fiji (Schindelin et al., 2012). The amounts of myrVenus in membrane and cytosol fractions were normalized by dividing the respective values by those of GAPDH and Na/K-ATPase. The ratio of normalized cytosolic myrVenus to normalized membrane myrVenus was calculated to assess the effect of the *Nmt1* knockout.

To detect FAK-Y925 phosphorylation, mEpiSCs were maintained as described in the 'Cell Line and Cell Culture' section. Subsequently, the medium was replaced with fresh DMED/F12 supplemented with non-essential amino acids, sodium pyruvate, and 0.1 mM of 2-mercaptoethanol, along with different concentrations of CGP77675, but without KSR, bFGF, or activin A for 19 h. Then, mEpiSCs were dissociated, resuspended in the same medium, and plated onto a culture dish coated with 20 μg/ml fibronectin (Cat. 354008, Corning). After 1 h, mEpiSCs were rinsed with PBS and lysed in 1× Laemmli sample buffer. Ten μg of proteins were separated and transferred to a PVDF membrane as described above. The membranes were probed with the primary antibody against Phospho-FAK (Tyr925) (1:1000, Cat. 3284, Cell Signaling). The secondary antibody and the protein detection method were the same as described above. The antibodies were stripped, and the total amount of FAK was assessed using the primary antibody against FAK (D2R2E) (1:1000, Cat. 13009, Cell Signaling). The remaining procedure for band detection followed the method described above. The inhibitory effect of CGP77675 on FAK phosphorylation was determined by dividing the band intensity of phospho-FAK by the total FAK band intensity for normalization. The results were presented by setting the value of the control (without CGP77675) to 1.

Bulk RNA-seq

For bulk RNA-seq, total RNA was extracted using the RNeasy Plus Mini Kit (Cat. 74136, Qiagen). Library preparation was performed using the TruSeq Stranded mRNA Library Prep Kit (Cat. 20020595, Illumina) according to the manufacturer's instructions. Sequencing was performed on an Illumina NovaSeq 6000 platform in paired-end mode (2x 101 nt). Sequenced reads were mapped to the mouse (mm10) or human (hg19) reference genome sequences using TopHat2 version 2.1.1 (Trapnell et al., 2009). Raw counts were calculated using featureCounts v. 2.0.0 (Liao et al., 2014). The fragments per kilobase of exon per million mapped fragments (FPKMs) were calculated using Cuffdiff v. 2.2.1 (Trapnell et al., 2010).

In creating heatmaps for human genes specific to the epiblast, trophectoderm, and primitive endoderm, we utilized the top 25 lineage-specific genes as reported by Petropoulos et al. (Petropoulos et al., 2016). Initially, we excluded genes whose FPKM values were zero in any of the six samples (three each from control and inhibitor-treated hiPSCs) due to the low accuracy of quantitative evaluation. As a result, four genes were excluded for the primitive endoderm, while all 25 genes were retained for both the epiblast and trophectoderm. Next, for each gene across all samples, we calculated relative FPKM values against the average FPKM values of three control samples. Finally, these values were expressed on a log2 scale, and heatmaps were generated using the pheatmap package (v. 1.0.12.) in R software (v. 4.4.1).

scRNA-seq

The scRNA-Seq libraries were constructed using the Chromium Next GEM Single Cell 5' Library and Gel Bead Kit v2 (Cat. 1000263, 10x Genomics) in accordance with the manufacturer's instructions. Sequencing of the libraries was performed on NovaSeq 6000 (Illumina) using paired-end mode (26x90 nt), achieving over 20,000 reads per cell. The sequencing data were aligned and quantified using Cell Ranger Single-Cell Software Suite (v.7.1.0, 10x Genomics) against the Homo sapiens (human) genome assembly GRCh38 and Mus musculus (mouse) genome assembly mm10 in 2020-A 10x Genomics reference packages. The gene count data were analyzed with the R package Seurat

(v5.0, Hao et al., 2024). MEF cells were filtered with more than 1% of the counts aligned to the mm10 genome. The cell doublets and low-quality cells were filtered using a unique molecular count threshold greater than 7,500 or less than 1000. Low-quality cells with more than 7% mitochondrial counts were filtered out. Then the gene count data were normalized and scaled with the Seurat SCTransform (vars.to.regress = "percent.mt") function. The datasets of the hiPSC samples, with or without treatment with the NMT inhibitor, were integrated using the R package Harmony (v1.0, Korsunsky et al., 2019) with the RunHarmony function for batch correction. All cells were first clustered with the Seurat FindNeighbors (reduction = “harmony”, dims = 1:30) and FindClusters (resolution = 0.2) functions. Dimensionality reduction was performed by UMAP embedding using the Seurat RunUMAP (reduction = “harmony”, dims = 1:30) functions. Subclustering of cluster 6 was conducted with the Seurat FindNeighbors (reduction = “harmony”, dims = 1:30) and FindClusters (resolution = 0.5) functions. Plots of individual gene expression amounts were visualized with the Seurat FeaturePlot, FeatureScatter, and RidgePlot functions.

Blastoid formation assay

Blastoid formation from naive hiPSCs was conducted according to the published protocol (Heidari Khoei et al., 2023) with some modifications. Briefly, naive hiPSCs cultured with or without 100 μM NMT inhibitor DDD85646 were dissociated using Accutase and plated onto gelatin-coated plates in the presence of 2.5 μM Y-27632 (Cat. 036-24023, FUJIFILM Wako Chemicals) for 1 h to remove MEF feeders. Unattached cells were centrifuged, washed with Ndiff227, and plated into each well of an Elplasia 96-well plate (Cat. 4442, Corning) in 150 μl aggregation medium (Heidari Khoei *et al.*, 2023) at a density of 6,000 cells per well. Next day (day 0), we added 150 μl 2× PALLY medium (Heidari Khoei *et al.*, 2023), consisting of 1 μM PD0325901, 1 μM A83-01 (ALK4/5/7 inhibitor; Cat. 2939, Tocris), 1 μM 1-oleoyl lysophosphatidic acid (LPA, Cat. 3854, Tocris), 20 ng/mL human LIF and 20 μM Y-27632 to each well. The efficiency of blastoid formation was assessed on day 4. We also used CEPT cocktail (Cat. 033-26071, FUJIFILM Wako Chemicals) in place of Y-27632 because the CEPT cocktail was reported to increase blastoid formation efficiency compared with Y-27632 (Yu et al., 2023).

Differentiation induction of hiPSCs toward trophectoderm lineage

Trophectoderm differentiation was performed following the published protocol (Io et al., 2021a; Io et al., 2021b). Briefly, naive hiPSCs cultured with or without 100 μM NMT inhibitor DDD85646 were dissociated using Accutase and plated onto gelatin-coated plates with 2.5 μM Y-27632 to remove MEF feeders. After two hours, unattached cells were replated onto 24-well plates coated with iMatrix-511 silk (Cat. T311, Takara Bio) at a density of 2×10^4 cells/cm^2 in differentiation induction medium (day 0). The differentiation induction medium consisted of Ndiff227 supplemented with 2 μM A83-1, 2 μM PD0325901 and 2.5 μM Y-27632. The NMT inhibitor was omitted during differentiation induction. On day 1, the medium was replaced with the same medium supplemented with 1 μM JAK inhibitor (Cat. 420099, Calbiochem). On day 3, cells were dissociated, stained with an APC-conjugated anti-human TROP2 antibody (1:100, Cat. 130-115-098, Miltenyi Biotech) and a PE-conjugated anti-human CD249 (ENPEP) antibody (1:100, Cat. 564533, BD Biosciences), and analyzed using a cell sorter (SH800, SONY Corp.).

REFERENCES

Bredenkamp, N., Yang, J., Clarke, J., Stirparo, G.G., von Meyenn, F., Dietmann, S., Baker, D., Drummond, R., Ren, Y., Li, D., et al. (2019). Wnt Inhibition Facilitates RNA-Mediated Reprogramming of Human Somatic Cells to Naive Pluripotency. Stem Cell Reports *13*, 1083-1098.

10.1016/j.stemcr.2019.10.009.

Cadinanos, J., and Bradley, A. (2007). Generation of an inducible and optimized piggyBac transposon system. Nucleic Acids Res *35*, e87. 10.1093/nar/gkm446.

Frearson, J.A., Brand, S., McElroy, S.P., Cleghorn, L.A., Smid, O., Stojanovski, L., Price, H.P., Guther, M.L., Torrie, L.S., Robinson, D.A., et al. (2010). N-myristoyltransferase inhibitors as new leads to treat sleeping sickness. Nature *464*, 728-732. 10.1038/nature08893.

Guo, G., von Meyenn, F., Rostovskaya, M., Clarke, J., Dietmann, S., Baker, D., Sahakyan, A., Myers, S., Bertone, P., Reik, W., et al. (2017). Epigenetic resetting of human pluripotency. Development *144*, 2748-2763. 10.1242/dev.146811.

Guo, G., Yang, J., Nichols, J., Hall, J.S., Eyres, I., Mansfield, W., and Smith, A. (2009). Klf4 reverts developmentally programmed restriction of ground state pluripotency. Development *136*, 1063-1069. 10.1242/dev.030957.

Heidari Khoei, H., Javali, A., Kagawa, H., Sommer, T.M., Sestini, G., David, L., Slovakova, J., Novatchkova, M., Scholte Op Reimer, Y., and Rivron, N. (2023). Generating human blastoids modeling blastocyst-stage embryos and implantation. Nat Protoc *18*, 1584-1620. 10.1038/s41596-023-00802-1.

Horie, K., Kokubu, C., Yoshida, J., Akagi, K., Isotani, A., Oshitani, A., Yusa, K., Ikeda, R., Huang, Y., Bradley, A., and Takeda, J. (2011). A homozygous mutant embryonic stem cell bank applicable for phenotype-driven genetic screening. Nat Methods *8*, 1071-1077. 10.1038/nmeth.1739.

Hotta, A., Cheung, A.Y., Farra, N., Vijayaragavan, K., Séguin, C.A., Draper, J.S., Pasceri, P., Maksakova, I.A., Mager, D.L., Rossant, J., et al. (2009). Isolation of human iPS cells using EOS lentiviral vectors to select for pluripotency. Nat Methods *6*, 370-376. 10.1038/nmeth.1325.

Io, S., Iemura, Y., and Takashima, Y. (2021a). Optimized protocol for naive human pluripotent stem cell-derived trophoblast induction. STAR Protoc *2*, 100921. 10.1016/j.xpro.2021.100921.

Io, S., Kabata, M., Iemura, Y., Semi, K., Morone, N., Minagawa, A., Wang, B., Okamoto, I., Nakamura, T., Kojima, Y., et al. (2021b). Capturing human trophoblast development with naive pluripotent stem cells in vitro. Cell Stem Cell *28*, 1023-1039 e1013. 10.1016/j.stem.2021.03.013.

Jacobs, J.P., Jones, C.M., and Baille, J.P. (1970). Characteristics of a human diploid cell designated MRC-5. Nature *227*, 168-170. 10.1038/227168a0.

Kranz, A., Fu, J., Duerschke, K., Weidlich, S., Naumann, R., Stewart, A.F., and Anastassiadis, K. (2010). An improved Flp deleter mouse in C57Bl/6 based on Flpo recombinase. Genesis *48*, 512-520. 10.1002/dvg.20641.

Laemmli, U.K. (1970). Cleavage of structural proteins during the assembly of the head of bacteriophage T4. Nature *227*, 680-685. 10.1038/227680a0.

Liao, Y., Smyth, G.K., and Shi, W. (2014). featureCounts: an efficient general purpose program for assigning sequence reads to genomic features. Bioinformatics (Oxford, England) *30*, 923-930. 10.1093/bioinformatics/btt656.

Niwa, H., Yamamura, K., and Miyazaki, J. (1991). Efficient selection for high-expression transfectants with a novel eukaryotic vector. Gene *108*, 193-199. 10.1016/0378-1119(91)90434-d.

Petropoulos, S., Edsgärd, D., Reinius, B., Deng, Q., Panula, Sarita P., Codeluppi, S., Plaza Reyes, A.,

Linnarsson, S., Sandberg, R., and Lanner, F. (2016). Single-Cell RNA-Seq Reveals Lineage and X Chromosome Dynamics in Human Preimplantation Embryos. Cell *165*, 1012-1026. 10.1016/j.cell.2016.03.023.

Rhee, J.M., Pirity, M.K., Lackan, C.S., Long, J.Z., Kondoh, G., Takeda, J., and Hadjantonakis, A.K. (2006). In vivo imaging and differential localization of lipid-modified GFP-variant fusions in embryonic stem cells and mice. Genesis *44*, 202-218. 10.1002/dvg.20203.

Schindelin, J., Arganda-Carreras, I., Frise, E., Kaynig, V., Longair, M., Pietzsch, T., Preibisch, S., Rueden, C., Saalfeld, S., Schmid, B., et al. (2012). Fiji: an open-source platform for biological-image analysis. Nature Methods *9*, 676-682. 10.1038/nmeth.2019.

Suemori, H., Yasuchika, K., Hasegawa, K., Fujioka, T., Tsuneyoshi, N., and Nakatsuji, N. (2006). Efficient establishment of human embryonic stem cell lines and long-term maintenance with stable karyotype by enzymatic bulk passage. Biochem Biophys Res Commun *345*, 926-932. 10.1016/j.bbrc.2006.04.135.

Takahashi, K., Tanabe, K., Ohnuki, M., Narita, M., Ichisaka, T., Tomoda, K., and Yamanaka, S. (2007). Induction of pluripotent stem cells from adult human fibroblasts by defined factors. Cell *131*, 861-872. 10.1016/j.cell.2007.11.019.

Takashima, Y., Guo, G., Loos, R., Nichols, J., Ficz, G., Krueger, F., Oxley, D., Santos, F., Clarke, J., Mansfield, W., et al. (2014). Resetting transcription factor control circuitry toward ground-state pluripotency in human. Cell *158*, 1254-1269. 10.1016/j.cell.2014.08.029.

Trapnell, C., Pachter, L., and Salzberg, S.L. (2009). TopHat: discovering splice junctions with RNA-Seq. Bioinformatics (Oxford, England) *25*, 1105-1111. 10.1093/bioinformatics/btp120.

Trapnell, C., Williams, B.A., Pertea, G., Mortazavi, A., Kwan, G., van Baren, M.J., Salzberg, S.L., Wold, B.J., and Pachter, L. (2010). Transcript assembly and quantification by RNA-Seq reveals unannotated transcripts and isoform switching during cell differentiation. Nat Biotechnol *28*, 511-515. 10.1038/nbt.1621.

Yagita, K., Horie, K., Koinuma, S., Nakamura, W., Yamanaka, I., Urasaki, A., Shigeyoshi, Y., Kawakami, K., Shimada, S., Takeda, J., and Uchiyama, Y. (2010). Development of the circadian oscillator during differentiation of mouse embryonic stem cells in vitro. Proc Natl Acad Sci U S A *107*, 3846-3851. 10.1073/pnas.0913256107.

Yu, L., Logsdon, D., Pinzon-Arteaga, C.A., Duan, J., Ezashi, T., Wei, Y., Ribeiro Orsi, A.E., Oura, S., Liu, L., Wang, L., et al. (2023). Large-scale production of human blastoids amenable to modeling blastocyst development and maternal-fetal cross talk. Cell Stem Cell *30*, 1246-1261 e1249. 10.1016/j.stem.2023.08.002.
